# Comparative metabolomic profiling and chemometric correlation of *Salvia rosmarinus* Spenn. and *Origanum vulgare* L. with antibacterial, antioxidant and anti-inflammatory activities

**DOI:** 10.1038/s41598-025-30607-z

**Published:** 2025-12-03

**Authors:** Alaa Y. Zakarya, Dalia M. Rasheed, Ayat M. Emad, Mohamed A. Farag, Zeinab M. Goda, Omnia Karem M. Riad, Sally T. K. Tohamy, Ahmed H. Elbanna

**Affiliations:** 1 Pharmacognosy Department, Faculty of Pharmacy, October 6 University, Sixth of October City, 12585 Egypt; 2 Pharmacognosy Department, Faculty of Pharmacy, Cairo University, Cairo, 11562 Egypt; 3 Healthcare Faculty, Saxony Egypt University, Badr City, Egypt; 4 Pharmaceutical Analytical Chemistry Department, Faculty of Pharmacy, Cairo University, Cairo, 11562 Egypt; 5 Department of Microbiology and Immunology, Faculty of Pharmacy (Girls), Al-Azhar University, Cairo, 11651 Egypt; 6 Department of Pharmaceutical Sciences, College of Pharmacy, Gulf Medical University, Ajman, 4184 United Arab Emirates

**Keywords:** Antibacterial, Anti-inflammatory, Antioxidant, Lamiaceae (mint family), Metabolite profiling, Partial least squares analysis (PLS), Biochemistry, Biological techniques, Chemical biology, Chemistry, Drug discovery, Microbiology, Plant sciences

## Abstract

The Lamiaceae plants are recognized in folk medicine for their antibacterial and anti-inflammatory properties. This study reports the first MS-based metabolomics analysis, integrating with chemometrics to explore metabolome heterogeneity in *Salvia rosmarinus* Spenn. (rosemary) and *Origanum vulgare* L. (oregano), and to pinpoint the key metabolites driving their antibacterial, antioxidant, and anti-inflammatory activities. UPLC–QTOF–MS/MS facilitated the identification of 164 metabolites, including flavonoids and hydroxycinnamic acids, which were reported for the first time in these species. For instance, salvianolic acid D and quercetin coumaroylhexoside were detected in rosemary, while salvianolic acid K, cleroden J, and flavonoids like nepitrin were newly reported in oregano. In biological evaluation, rosemary strongly inhibited methicillin-resistant *Staphylococcus aureus* and *Escherichia coli*, exhibited the highest radical scavenging capacity in DPPH assay, and showed superior anti-inflammatory effects through COX-II inhibition and TNF-α and NF-κB suppression. In contrast, oregano displayed the strongest reducing power in FRAP assay. Chemometric analyses revealed that flavonoids, hydroxycinnamic acids, and terpenes were the principal discriminating classes. Partial least squares analysis correlated rosemary’s antibacterial and radical scavenging activity with hydroxycinnamic acids, flavonoids, and terpenes, while its anti-inflammatory effects were linked to flavonoids and diterpenes. In oregano, FRAP reducing power correlated with benzyl derivatives, organic acids, and hydroxycinnamic acids.

**Supplementary Information:**

The online version contains supplementary material available at 10.1038/s41598-025-30607-z.

## Introduction

The Lamiaceae family (mint family) encompasses a broad range of aromatic plants that are widely distributed but particularly prevalent in the Mediterranean region^1^. Lamiaceae plants have been used extensively as home remedies and to enhance the taste and aroma of foods since ancient times^2^. The mint family has long been used for decades in cosmetics and conventional medicine as antiseptics, carminatives, expectorants, and sedatives^2^, in addition to its potential antioxidant and anti-inflammatory properties^3,4^. Herbs within Lamiaceae are recognized for their diverse chemical composition, particularly their essential oils, which contribute to their aroma and biological activities^5^. In addition to essential oils, Lamiaceae plants are rich in non-volatile secondary metabolites *vis.* hydroxycinnamic acids (e.g., rosmarinic acid, salvianolic acid isomers, caffeic acid), flavonoids (e.g., naringenin, apigenin, luteolin, quercetin), phenolic abietane diterpenoids (e.g., carnosol, carnosic acid, rosmanol), and other phenolics^6,7^. Owing to their rich and diverse phytochemical composition, Lamiaceae plants exhibit many biological activities, including antioxidant, antibacterial, antiviral, and antifungal effects^8,9^. In particular, the antimicrobial activity is especially pronounced in their essential oils^10^. This generally highlights the broad biological potential of plants, as evidenced by continuous reporting of various biological activities of different medicinal plants and/or their derived metabolites including, but not limited to, antioxidant, anti-inflammatory, enzyme inhibitory, and cytotoxic effects^11,12^.

Rosemary (*Salvia rosmarinus* Spenn., previously known as *Rosmarinus officinalis* L.) and oregano (*Origanum vulgare* L.) are mint family plants widely grown in many diverse regions worldwide^13^. Traditionally, these plants have long been used in relieving colds and coughs, and as immunomodulators owing to their pronounced antimicrobial and anti-inflammatory activities^14^. These traditional uses highlight their therapeutic importance, which has motivated the exploration of sustainable sources of their active constituents. In this regard, our earlier work underscored the potential of the different distillation by-products as promising antibacterial, antioxidant, and anti-inflammatory resources^15^.

This study aims to compare the MS-based phytochemical profiles of these Lamiaceae species in correlation to their antimicrobial, antioxidant, and anti-inflammatory activities, given the interconnected relationship between oxidative stress, inflammation, and microbial infection, which collectively play a crucial role in the development of many diseases and often overlap in both cause and consequences^16^. Furthermore, this study seeks to underpin chemical constituents responsible for well-characterized biological effects using chemometric tools. To achieve such a goal, multivariate data analysis (MVA) represented by partial least squares analysis (PLS) was employed to identify relationships between metabolite profiles and associated biological activities for the first time in Lamiaceae and discern the metabolites contributing the most and the least to the studied biological activities. Given that *S. rosmarinus* Spenn. and *O. vulgare* L. are also widely used in traditional hygienic practices, mainly in aqueous preparations in the form of infusions or decoctions, antibacterial activity was additionally evaluated for the aqueous extracts to better represent their ethnopharmacological applications.

## Materials and methods

### Plant material

The aerial parts of *S. rosmarinus* Spenn. and *O. vulgare* L. were acquired from the Medicinal, Aromatic, and Poisonous Plants Experimental Station (MAPPES), Faculty of Pharmacy, Cairo University (Giza, Egypt) in June 2022 (early summer), before the flowering stage. Plants were authenticated supervisors and managers of the MAPPES. Voucher specimens were deposited at the Herbarium of the Pharmacognosy Department, Faculty of Pharmacy, Cairo University (specimens’ numbers: *S. rosmarinus* Spenn.; 18.4.24-F, and *O. vulgare* L.; 17.4.24-F).

### Preparation of plant extracts for biological evaluation

Shade-dried, pulverized plants (100 g, each) were extracted separately by maceration till exhaustion using 80% methanol in water to yield alcoholic extracts of *S. rosmarinus* Spenn. (RO) and *O. vulgare* L. (OV). After extraction, the solutions were filtered through Whatman No.1 filter paper and then concentrated under reduced pressure at 40 °C using a rotary evaporator (R-210 evaporator, Büchi, Switzerland) to yield (19.5 g RO, 17.5 g OV). The samples were stored in airtight dark containers and kept in a refrigerator at 4 °C till further assays.

Aqueous extracts of *S. rosmarinus* Spenn. (Aq.RO) and *O. vulgare* L. (Aq.OV) were prepared following the same procedure, using distilled water instead of methanol. The resulting yields were 7 g for Aq.RO and 10.5 g for Aq.OV. These aqueous extracts were used exclusively for evaluating antimicrobial activity.

### Preparation of extracts and UPLC-ESI–QTOF-MS analysis conditions

Dried pulverized plants (30 mg of each powder) were mixed separately in 2 mL 80% HPLC grade methanol with 10 µg/mL umbelliferone (an internal standard), using a Turrax mixer (11000 RPM) for five 20-sec periods, then centrifuged at 3000*g* (4 °C, 15 min) to exclude plant debris, followed by filtration using a 22 μm pore-size filter (Agilent, USA).

An ACQUITY UPLC system (Waters, Milford, MA, USA) was used for UPLC-ESI-QTOF-MS analysis. Chromatographic separation was carried out by injection of alcoholic extracts (3.1 µL) on HSS T3 column (100 × 1.0 mm, particle size 1.8 μm; Waters) at a temperature of 40 °C, where mobile phase A was 0.1% formic acid in water, and mobile phase B was acetonitrile. The flow rate was maintained at 0.15 mL min⁻¹, with the following gradient program: 0–1 min, 5% B; 1–11 min, linear increase from 5% to 100% B; 11–19 min, 100% B; 19–20 min, decrease from 100% to 5% B; and finally, 20–25 min, 5% B. The analytical parameters of the instrument used were previously detailed^17^. The system was coupled to 6540 Agilent Ultra-High-Definition Accurate Mass Q-TOF LC/MS (Palo Alto, CA, USA) using an electrospray ionization (ESI) source in both positive and negative ion modes. The operating conditions were applied as described by Baky et al.^17^, with a fragmentation voltage of 100 V. The Mass Hunter Workstation software (Agilent Technologies) was used for handling data acquisition. Compounds were assigned by comparing retention times (R_t_), exact masses, and characteristic fragmentation patterns (MS2), as well as the candidates’ molecular formula (with 10 ppm mass accuracy limit), and data previously reported in other works of literature.

### Antibacterial activity evaluation

#### Bacterial strains and culture conditions

Antibacterial activity of the aqueous and hydroalcoholic extracts of *S. rosmarinus* Spenn. and *O. vulgare* L. was carried out against two standard bacterial strains: Methicillin-resistant *Staphylococcus aureus* (MRSA) ATCC 43,300 as Gram-positive bacteria, and the Gram-negative bacteria *Escherichia coli* ATCC 25,922. The used bacterial strains were available in the stock culture in the Microbiology and Immunology Department, Faculty of Pharmacy (Girls), Al-Azhar University, Giza, Egypt. Nutrient agar, Mueller-Hinton agar, LB broth, and tryptic soy broth were purchased from Oxoid (Hampshire, UK). All reagents and chemicals for buffers were purchased from Sigma-Aldrich Co. (St. Louis, MO, USA).

#### Minimum inhibitory concentration (MIC) assessment using the broth microdilution method

For anti-microbial susceptibility testing, minimum inhibitory concentrations (MICs) were determined by the broth microdilution method according to the Clinical and Laboratory Standards Institute (CLSI) against standard strains of *E. coli* and MRSA^18^. The extracts were prepared in dimethyl sulfoxide (DMSO) at final concentrations ranging from 512 µg/mL to 16 µg/mL. MICs were carried out in triplicate (*n* = 3), and doxycycline was used as a reference drug control.

#### Biofilm Inhibition assay

The biofilm formation inhibition assay was conducted against MRSA isolates to evaluate the biofilm formation inhibition potential of the tested samples. The biofilm formation inhibition was determined by measuring the absorbance of the adherent biofilms following treatment and comparing these values with those obtained from the untreated controls. A 100 µL of bacterial suspension (1.5 × 10^8^ CFU) in trypticase soy broth supplemented with 1% glucose was added to each well of flat-bottom microtiter plates. Then 100 µL of ¼ MIC of each extract was added to the corresponding well. In each microtiter plate, 6 wells were assigned for positive and negative controls. After incubation at 37 °C for 24 h, the microtiter plates were decanted and washed three times with 250 µL of sterile phosphate-buffered saline (PBS) pH 7.2, fixed by drying for 1 h at 60 °C, then stained with 200 µL of 0.1% w/v crystal violet, and kept at room temperature for 15 min. Finally, the microtiter plates were washed with distilled water, dried, filled with 200 µL of 33% acetic acid, and transferred (150 µL) to a new plate. A microplate reader (Tecan Elx800, USA) was used to measure the optical densities at 630 nm, as performed by Badawy et al.^19^. The results were represented as a biofilm formation inhibition percentage, calculated using the following equation:

$$\: \% \:Inhibition\:of\:biofilm\:formation\: = \:1 - \frac{{Optical\:density\:of\:sample\:}}{{Optical\:density\:of\:control\:\left( {untreated} \right)}}\: \times \:100$$

The assay was performed in triplicate ± SD (*n* = 3).

#### RNA extraction and qRT-PCR-based relative gene expression analysis

The effectiveness of extracts (RO/Aq.OV) to inhibit expression of the *agrA*, *icaA* gene was assessed using quantitative real-time PCR.

MRSA was cultured overnight at 37 °C in LB broth with and without extracts at a concentration of ¼MIC. The total RNA of the cultured MRSA was extracted and converted to DNA using the First High Pure RNA Isolation Kit (Roche Diagnostics GmbH, Germany) and QuantiTects Reverse Transcription Kit (Qiagen, USA), respectively, following the procedure of Saleh et al.^20^.

*AgrA* and *icaA* virulence genes were amplified using qRT-PCR in accordance with the instructions provided by One-Step Kit (Bioline, UK). The StepOne RT-PCR thermal cycler (Applied Biosystem, USA) was used to set up the qRT-PCR analysis.

The forward and reverse gene-specific PCR primers for *AgrA*, and *icaA* were (5′-GGA GTG ATT TCA ATG GCA CA-3′; 5′-ATC CAT TTT ACT AAG TCA CCG ATT-3′), and (5′-CAATACTATTTCGGGTGTCTTCACTCT-3; 5′-CAAGAAACTGCAATATCTTCGGTAATCAT-3′), respectively^21^. The relative expression values of each gene were normalized to the value of the housekeeping gene 16 S rRNA. The forward and reverse primers used for 16 S rRNA were 5′-TGT CGT GAG ATG TTG GG-3′, and 5′-TGT CGT GAG ATG TTG GG-3′, respectively^21^. 2^−∆∆CT^ method was used to calculate the results^22^. The results were reported as means ± SD of triplicate measurements.

### Antioxidant activity evaluation

In vitro antioxidant activity of methanolic extracts of *S. rosmarinus* Spenn. and *O. vulgare* L. was evaluated using 2,2-diphenyl-1-picrylhydrazyl (DPPH) and ferric-reducing antioxidant power (FRAP) assays. Color change of reaction mixtures resulting from the antioxidant activity of the extracts was monitored using a UV spectrophotometer (UV-1601 PC, Shimadzu, Kyoto, Japan), as follows:

#### 2,2-Diphenyl-1-picrylhydrazyl assay (DPPH) assay

The free radical scavenging effect of the samples was evaluated as per the method previously described by Karaçelik et al.^23^. The reaction was performed by mixing the sample with DPPH at room temperature (25 °C) and incubating the mixture in the dark for 30 min. The absorbance was then recorded at 516 nm. Quenching of the color intensity of the DPPH indicates the scavenging activity of the extracts. The free radical scavenging activities were expressed as percent inhibition using the equation:

$$Inhibition\:\left( \% \right) = \frac{{Absorbance\:of\:control - Absorbance\:of\:sample}}{{Absorbance\:of\:control}} \times 100$$

The measurements were performed in triplicate, and the results are presented as mean ± SD.

#### FRAP assay

The ferric ion-reducing capacity of alcoholic extracts was measured kinetically following the method described by Benzie and Strain^24^. The FRAP reagent was prepared by mixing 300 mM sodium acetate trihydrate buffer (pH 3.6), 10 mM TPTZ (2,4,6-tripyridyl-*s*-triazine) dissolved in 40 mM hydrochloric acid, and 20 mM ferric chloride at a ratio 10:1:1 υ/υ/υ, respectively. Freshly prepared FRAP reagent (1.5 mL) was added to 50 µL of each extract (concentration = 0.1 mg/mL). Over three minutes, the absorbance change (resulting from the production of a ferrous ion blue color) was monitored at 593 nm. The results were expressed as µmol/L FeSO_4_.7H_2_O equivalent/mg of each extract.

### Anti-inflammatory activity evaluation

#### COX-II Inhibition assay

Cyclooxygenase II (COX-II) Inhibitor Assay Kit from Abcam (the USA): ab211097 was used to evaluate the samples’ inhibitory activity. The reaction conditions and method comply with the manufacturer’s guidelines, using 10-fold serial dilutions (100, 10, 1, 0.1, 0.01 µg/mL). The process is based on detecting prostaglandin G2, a metabolite from arachidonic acid developed by COX-II activity, fluorometrically. Spectrofluorometer Tecan Spark (Tecan Group Ltd., Switzerland) was used to measure the fluorescence of the samples (Ex/Em = 535/587 nm) kinetically for 5–10 min at 25 °C. The IC_50_ was determined by plotting % inhibition of enzyme activity against sample concentrations. All sample assays were performed in triplicate.

#### Tumor necrosis factor-alpha (TNF-α) and nuclear factor kappa-B (NFқb) quantification

The macrophage cell line RAW 264.7, used for the in vitro anti-inflammatory assay, was obtained from the American Type Culture Collection (Manassas, Virginia, USA). The cells were cultivated in Dulbecco’s Modified Eagle’s medium (DMEM) with 10 µg/mL insulin, 1% penicillin-streptomycin, and 10% fetal bovine serum at 37 °C. The reagents were all of molecular biology grade.

The levels of tumor necrosis factor-alpha (TNF-α) and nuclear factor kappa-B (NF-κB) were quantitatively assessed in lipopolysaccharide (LPS) stimulated RAW 264.7 macrophages using enzyme-linked immunosorbent assay (ELISA) kits (human TNF-α ELISA Kit, Abcam, USA: ab181421; human NF-κB p100/NFKB2 ELISA Kit, Abcam, USA: ab288581). All procedures were conducted according to the manufacturers’ protocols. For both assays, absorbance was measured at 450 nm using a microplate reader (BIOLINE ELISA Microplate Reader for TNF-α; ROBONIK P2000 ELISA Reader for NF-κB). The equivalent concentrations were determined from standard curves, and all measurements were carried out in triplicate.

### Statistical analysis

All experiments were performed in triplicate, and the results are expressed as mean ± standard deviation (SD). Data was analyzed using GraphPad Prism version 8.0.1 (GraphPad Software Inc., California, USA). Statistical differences between two independent groups were assessed using an unpaired *t*-test. For analyses involving more than two groups, one-way ANOVA was applied, followed by Tukey’s post hoc test for multiple comparisons. A *p*-value < 0.05 was considered statistically significant.

### Multivariate data analysis

The data processing software MZmine 3.3 (available at https://github.com/mzmine/mzmine3) was employed for peak detection, deconvolution, deisotoping, and alignment of the imported mzXML files^25^. This workflow produced an aligned peak list, which served as the basis of a detailed data matrix incorporating information from all samples (in triplicate). The negative ESI mode demonstrated greater sensitivity for a broader range of expected metabolite classes compared to the positive ESI mode^26^. The data matrix included columns detailing the scan number, retention time (t_R_), mass-to-charge ratio (*m/z*), and peak intensity of the eluted compounds. Subsequently, the dataset was exported to SIMCA-P (version 14.1, Umetrics, Ume, Sweden) for Pareto scaling before multivariate analysis (MVA).

Partial least squares (PLS) analysis was applied to establish associations between bioactivities and the UPLC-QTOF-MS/MS dataset of annotated metabolites. Significant metabolites contributing to bioactivity were identified using variable importance in projection (VIP) scores derived from the PLS model. Additionally, Pearson’s correlation coefficient (r) was calculated for correlation analysis, and a correlogram was generated to visually represent the strength of the correlations between metabolites and the evaluated bioactivity. This visualization was created using the MetaboAnalyst 5.0 platform (https://metaboanalyst.ca/). The thresholds for interpreting correlation coefficients were defined as follows: negligible correlation for *r* < 0.3, weak correlation for *r* = 0.3–0.5, moderate correlation for *r* = 0.5–0.7, strong correlation for *r* = 0.7–0.9, and very strong correlation for *r* = 0.9–1.0^27^.

## Results and discussion

### UPLC–QTOF-MS/MS metabolite profiling of *Salvia rosmarinus* Spenn. and *Origanum vulgare* L. extracts

**Fig. 1:**
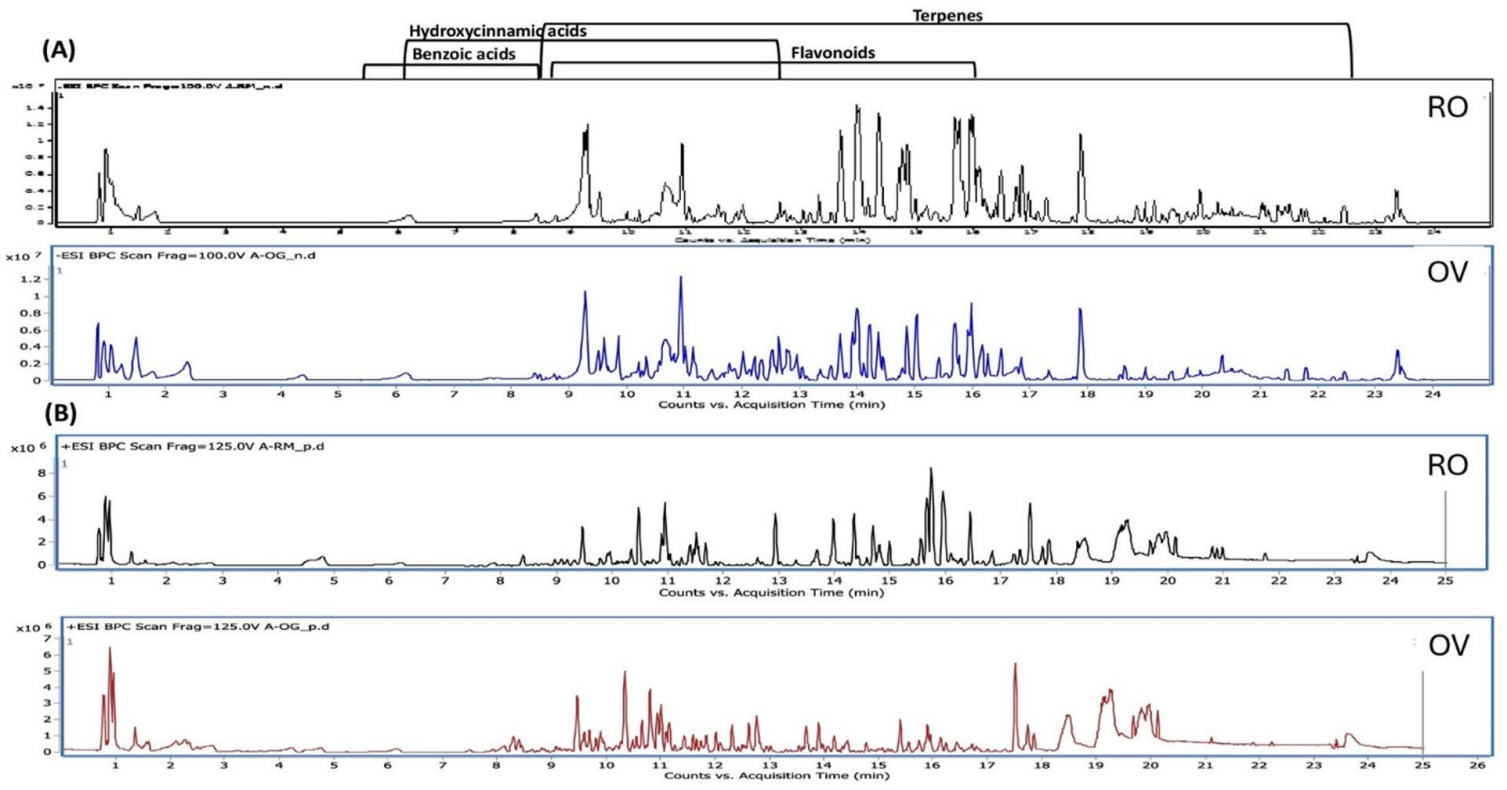
Base peak chromatogram (BPC) of rosemary (RO) and oregano (OV) extracts analyzed by UPLC–QTOF-MS/MS in both negative (**A**) and positive (**B**) ion modes.

**Fig. 2:**
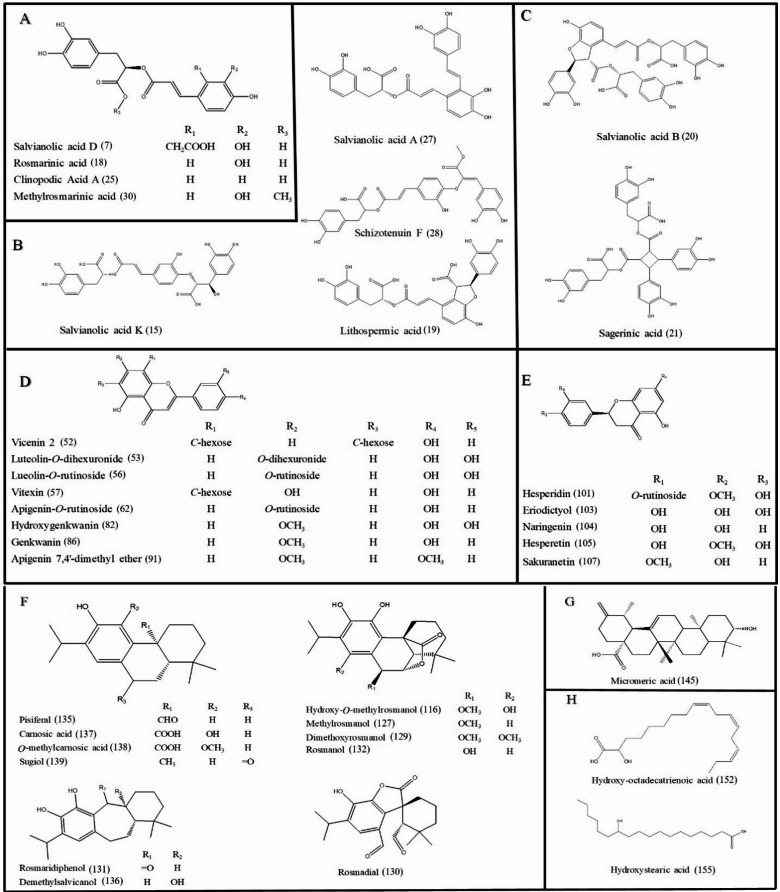
Chemical structures of the major classes of metabolites identified in the rosemary & oregano extracts. The numbers listed refer to the identified metabolites listed in Table 3. (**A**) Caffeic acid dimers, (**B**) caffeic acid trimers, (**C**) caffeic acid tetramer, (**D**) flavones, (**E**) flavanones, (**F**) abietane diterpenoids, (**G**) triterpenoids, (**H**) fatty acids.

To identify the metabolites likely to mediate the aforementioned antimicrobial, antioxidant, and anti-inflammatory effects, UPLC–QTOF–MS/MS was used in both ionization modes (positive and negative) to provide comprehensive detection of metabolites in *S. rosmarinus* Spenn. and *O. vulgare* L. extracts. Base peak chromatograms (BPC) of *S. rosmarinus* Spenn. and *O. vulgare* L. extracts are presented in Fig. 1. The analysis resulted in the detection of 164 compounds within 25 min. UPLC run, of which 92 were detected in both extracts, including hydroxycinnamic acid derivatives, flavonoid derivatives, benzoic acid derivatives, terpenes, and organic acids, as summarized in Table 1. The chemical structures of the major classes of metabolites are shown in Fig. 2. Details of the assigned metabolites are discussed in the following subsections.

**Table 1:** Identified metabolites in *S. rosmarinus* Spenn. And *O. vulgare* L. extracts via UPLC–QTOF–MS/MS in negative/positive ionization modes. All molecular formulae were assigned with a mass accuracy limit of ± 10 Ppm & the bolded fragments represent the base peak ions.

No.	*R*_t_. (min.)	Metabolite name	Mol. Ion m/z		Δ mass (ppm)	Molecular formula	MS^2^ ions m/z (-)/(+)	Extract
			M-H	M + H				
Short-chain carboxylic acids								
1	0.99	Malic acid	133.0140		1.84	C_4_H_6_O_5_	115, 89, 71, 59	RO, OV
2	1.33	Citric acid	191.0192		2.74	C_6_H_8_O_7_	111, 87, 85	RO, OV
3	1.72	Succinic acid	117.0910		2.81	C_4_H_6_O_4_	99, 73	RO, OV
4	8.68	2-Isopropylmalic acid	175.0608		2.26	C_7_H_12_O_5_	131, 115, 85	RO, OV
5	11.43	Oxoadipic acid	159.0296		1.86	C_6_H_8_O_5_	113, 73, 68	OV
Hydroxycinnamic acids								
6	6.10	Danshensu (Salvianic acid A)	197.0450		2.26	C_9_H_10_O_5_	179, 151, 135, 123	RO, OV
7	6.20	Salvianolic acid D	417.0791		8.66	C_20_H_18_O_10_	219, 197, 179, 173	RO, OV
8	8.32	Hydroxyphenyllactic acid	181.0502		2.37	C_9_H_10_O_4_	163, 135, 119, 72	RO, OV
9	8.59	Coumaric acid-O-hexoside	325.0921		2.43	C_15_H_18_O_8_	163, 119	RO, OV
10	8.63	Caffeic acid-O-hexoside	341.0869		2.65	C_15_H_18_O_9_	179, 161, 135	RO, OV
11	9.04	Caffeic acid	179.0345	181.0495	2.68 /0.2	C_9_H_8_O_4_	135, 134, 117, 107 /163, 145, 135, 117	RO, OV
12	9.14	Neochlorogenic acid (O-caffeoylquinic acid)	353.0868	355.0998	2.84 /7.23	C_16_H_18_O_9_	235, 191, 179, 13,173, 161, 135 /325, 309, 193, 163	RO, OV
13	9.62	O-coumaroylquinic acid	337.0921	339.1049	2.34 /7.52	C_16_H_18_O_8_	319, 293, 191, 163 /321, 221, 147, 175, 136	RO
14	9.77	Coumaryl alcohol dihexoside	519.1704		2.94	C_21_H_30_O_12_ (formate adduct)	473, 311, 149	RO
15	10.08	Salvianolic acid K	555.1129		2.72	C_27_H_24_O_13_	511, 357, 295, 197, 179, 135	RO, OV
16	10.21	Rosmarinic acid-O-hexoside (Salviaflaside)	521.1285		3	C_24_H_26_O_13_	359, 323, 197, 179, 161, 135	RO
17	10.23	Coumaric acid	163.0397	165.0546	2.24 /0.13	C_9_H_8_O_3_	119, 117, 103, 93 /147, 119, 91	RO, OV
18	10.41	Rosmarinic acid	359.0766	361.0919	1.78 /-0.29	C_18_H_16_O_8_	197, 179, 161, 135 /181, 163, 139, 135	RO, OV
19	10.51	Lithospermic acid	537.1021		3.25	C_27_H_22_O_12_	493, 359, 295, 179	OV
20	10.53	Salvianolic acid B	717.1437		3.35	C_36_H_30_O_16_	673, 519, 359, 321, 197	RO, OV
21	10.60	Sagerinic acid	719.1602		2.16	C_36_H_32_O_16_	539, 495, 359, 197, 179	RO, OV
22	10.80	Lithospermic acid derivative	897.1848		3.97	C_45_H_38_O_20_	537, 493, 471, 359	OV
23	11.31	Yunnaneic acid F	597.1231	599.1392	3.14 /0.56	C_29_H_26_O_14_	359, 197, 179, 161 /387, 359, 317, 181	RO
24	11.34	Ferulic acid	193.0501	195.0651	3.26 /0.44	C_10_H_10_O_4_	161, 135, 134, 133 /179, 161, 149, 133, 119, 117	RO, OV
25	11.49	Clinopodic acid A	343.0813		2.98	C_18_H_16_O_7_	197, 161, 145, 135	RO, OV
26	11.64	Schizotenuin C1	535.0865		3.17	C_27_H_20_O_12_	359, 197, 177, 161	OV
27	11.71	Salvianolic acid A	493.1127		2.67	C_26_H_22_O_10_	359, 295, 197, 179, 161	OV
28	11.99	Methyl melitrate A (Schizotenuin F)	551.1179		2.9	C_28_H_24_O_12_	519, 359, 179, 161, 135	OV
29	12.00	Coumaryl alcohol	149.0605		2.02	C_9_H_10_O_2_	131, 119, 108, 103	RO
30	12.09	Methylrosmarinic acid	373.0917		3.18	C_19_H_18_O_8_	193, 197, 179, 161, 135	RO, OV
31	12.68	Nepetoidin isomer	313.0709		2.74	C_17_H_14_O_6_	161, 151, 133	RO, OV
32	12.68	Vinyl caffeate	205.0503		1.61	C_11_H_10_O_4_	161, 133	RO, OV
33	12.97	Cleroden J	553.1339		2.26	C_28_H_26_O_12_	521, 477, 373, 179, 135	OV
Benzoic acid derivatives								
34	5.36	Protocatechuic acid hexoside	315.0712		3.02	C_13_H_16_O_9_	153, 109	RO, OV
35	7.98	Vanillic acid		169.0495	0.21	C_8_H_8_O_4_	151, 138, 125, 110, 65	RO, OV
36	7.99	Vanillic acid hexoside	329.0868		3.05	C_14_H_18_O_9_	167, 123, 121, 89	RO, OV
37	8.17	Hydroxytyrosol-O-hexoside	315.1077		2.66	C_14_H_20_O_8_	153, 137, 123	RO, OV
38	8.43	Syringic acid		199.0600	0.5	C_9_H_10_O_5_	181, 140, 125, 107	RO, OV
39	8.60	Protocatechuic acid		155.033	5.75	C_7_H_6_O_4_	137, 109, 81	RO, OV
40	8.79	Hydroxybenzoic acid-O-hexoside	299.0763		3.14	C_13_H_16_O_8_	137, 93	RO, OV
41	8.82	Hydroxybenzoic acid	137.0241	139.0390	2.3 /-0.21	C_7_H_6_O_3_	93 /121, 93, 65	RO, OV
Benzyl derivatives								
42	1.94	Hydroxyphenol hexoside	271.0814		3.4	C_12_H_16_O_7_	109, 108, 71	OV
43	2.26	Hydroxyphenol hexoside derivative	389.1075		3.68	C_16_H_22_O_11_	271, 161, 113, 101,73	OV
44	4.85	Calleryanin	301.0922		2.29	C_13_H_18_O_8_	225, 139, 121	OV
45	9.48	Benzyl alcohol-O-hexosyl-pentoside	401.1441	403.1575	3.04 /8.59	C_18_H_26_O_10_	269, 225, 161, 101, 73 /385, 333, 271	RO, OV
46	9.63	Origanine B or C	813.1858		3.15	C_35_H_35_O_20_	769, 615, 571, 303	OV
47	12.16	Origanoside	435.1287		2.23 /5.56	C_21_H_24_O_10_	389, 227, 136, 92	OV
48	12.87	Orthosiphoic acid A		523.1235	-0.02	C_27_H_22_O_11_	371, 325, 163, 153	OV
Jasmonic acid derivatives								
49	9.21	Tuberonic acid hexoside	387.1651		2.46	C_18_H_28_O_9_	225, 207, 163, 59	RO, OV
50	9.87	Epi hydroxyjasmonic acid (Tuberonic acid)	225.1128	227.1278	1.91 /-0.06	C_12_H_18_O_4_	181, 163, 97, 59 /209, 191, 163, 149	RO, OV
51	14.81	Methyl jasmonate		225.1485	0.09	C_13_H_20_O_3_	207, 151, 133, 109	RO
Flavones								
52	9.45	Apigenin 6,8-di-C-hexoside (Vicenin 2)	593.1491	595.1657	3.52 /0.08	C_27_H_30_O_15_	503, 473, 383, 353 /541, 511, 475, 457	RO, OV
53	9.69	Luteolin-O-dihexuronide	637.1026		3.19	C_27_H_26_O_18_	351, 285	OV
54	9.85	Hydroxyluteolin-O-hexoside	463.0870	465.1029	2.58 /-0.32	C_21_H_20_O_12_	301, 191, 161 /303, 257, 163	RO
55	9.97	Luteolin-O-hexosyl-hexoside		611.1604	0.43	C_27_H_30_O_16_	449, 287	OV
56	10.16	Luteolin-O-rutinoside	593.1495	595.1655	2.85 /0.42	C_27_H_30_O_15_	285, 267, 217, 197 /449, 287, 147, 129	RO, OV
57	10.23	Vitexin		433.1118	2.6	C_21_H_20_O_10_	415, 397, 379, 337, 313, 283	OV
58	10.26	Luteolin-O-hexuronide	461.0712	463.0876	2.92 /-1.08	C_21_H_18_O_12_	357, 285 /287, 153	RO, OV
59	10.29	Luteolin-O-hexoside		449.1078	0.08	C_21_H_20_O_11_	287, 269	RO, OV
60	10.49	Nepitrin (6-Methoxyluteolin-O-hexoside)	477.1027	479.1183	2.4 /0.21	C_22_H_22_O_12_	315, 197, 161, 153 /317, 302, 285, 165	RO, OV
61	10.53	Luteolin-O-pentosyl-hexoside		581.1498	0.15	C_26_H_28_O_15_	435, 419, 287, 271	OV
62	10.54	Apigenin-O-rutinoside		579.1693	2.65	C_27_H_30_O_14_	433, 271	RO
63	10.67	Hispidulin-O-rutinoside		609.1808	0.98	C_28_H_32_O_15_	463, 301, 286	RO, OV
64	10.78	Luteolin-O-pentosyl-acetyl-hexoside		623.1605	0.26	C_28_H_30_O_16_	583, 449, 287	OV
65	10.78	Apigenin-O-hexoside		433.1128	0.29	C_21_H_20_O_10_	271, 185, 153, 119, 109	RO, OV
66	10.81	Apigenin-O-hexuronide	445.0762	447.0921	3.22 /0.2	C_21_H_18_O_11_	427, 341, 269, 225 /271, 203, 153, 109	RO, OV
67	11.18	Luteolin caffeoylhexoside		611.1390	0.87	C_30_H_26_O_14_	449, 287, 163, 153	OV
68	11.20	Apigenin-O-pentosyl-acetyl-hexoside		607.1657	0.08	C_28_H_30_O_15_	463, 287, 271, 199, 163	OV
69	11.53	Feruloylnepitrin	653.1489	655.1655	3.51 /0.38	C_32_H_30_O_15_	477, 315 /479, 317, 177	RO
70	11.60	Luteolin-acetyl-O- hexuronide	503.0818	505.0978	2.61 /-0.26	C_23_H_20_O_13_	443, 399, 285, 133 /463, 287, 201	RO
71	11.60	Acacetin-O-rutinoside (Linarin)	637.1752		3.46	C_28_H_32_O_14_(formate adduct)	591, 283, 268	OV
72	11.86	Luteolin	285.0399	287.0549	1.96 /0.4	C_15_H_10_O_6_	267, 257, 241, 217, 199, 175, 151, 133 /269, 219, 153, 135	RO, OV
73	11.92	Diosmetin-O-pentosyl-acetyl-pentoside		607.1657	0.08	C_28_H_30_O_15_	565, 302, 301, 286, 229	OV
74	12.00	Acacetin-O-hexuronide	459.0920	461.1063	2.79 /3.34	C_22_H_20_O_11_	283, 175, 113 /285, 270, 255, 153	OV
75	12.08	Hispidulin-O-hexoside (Homoplantaginin)	461.1076	463.1235	2.89 /-0.03	C_22_H_22_O_11_	299, 284 /301, 286	RO
76	12.39	Thymusin		331.0813	-0.21	C_17_H_14_O_7_	316, 301, 273, 181, 119	OV
77	12.56	Apigenin	269.0451	271.0603	1.66 /-0.74	C_15_H_10_O_5_	225, 201, 151, 149, 117, 107 /243, 153, 119, 109	RO, OV
78	12.74	Diosmetin	299.0554		2.04	C_16_H_12_O_6_	284, 256, 179, 151, 107	RO, OV
79	12.85	Hydroxyluteolin 7,3’-dimethyl ether		331.0813	-0.21	C_17_H_14_O_7_	316, 298, 270, 136	OV
80	13.34	Majoranin (Thymonin)	359.0763	361.0919	2.61 /-0.29	C_18_H_16_O_8_	344, 329, 314 /346, 331, 313	OV
81	13.51	Hispidulin	299.0555	301.0706	2.04 /0.22	C_16_H_12_O_6_	284, 256, 136.9, 117 /286, 121, 112	RO, OV
82	13.53	Hydroxygenkwanin (7-methylluteoline)	299.0554		2.37	C_16_H_12_O_6_	284, 256, 227, 151, 133	OV
83	13.69	Cirsimaritin	313.0710	315.0864	2.43 /-0.27	C_17_H_14_O_6_	297, 283, 163, 135, 117 /300, 282	RO, OV
84	13.90	Cirsilineol	343.0816	345.0970	2.11 /-0.35	C_18_H_16_O_7_	328, 313, 283, 147 /330, 312, 284	OV
85	14.18	Xanthomicrol	343.0816	345.0970	2.11 /-0.35	C_18_H_16_O_7_	328, 313, 298, 117 /330, 315, 297, 119	OV
86	14.32	Genkwanin (7-Methylapigenin)	283.0605	285.0758	2.45 /-0.18	C_16_H_12_O_5_	268, 211, 135, 117 /270, 167, 119	RO, OV
87	14.42	Pebrellin	373.0919	375.1074	2.65 /0.12	C_19_H_18_O_8_	358, 343, 161, 147 /360, 345, 327, 169	OV
88	14.61	Ladanein	313.0709	315.0864	2.74 /-0.27	C_17_H_14_O_6_	298, 283, 255, 148, 132 /300, 229, 133	RO, OV
89	14.93	Gardenin B		359.1123	0.64	C_19_H_18_O_7_	344, 326, 298, 209	OV
90	15.52	Salvigenin		329.1019	0.2	C_18_H_16_O_6_	314, 296, 268	RO, OV
91	16.25	Apigenin 7,4’-dimethyl ether		299.0914	0	C_17_H_14_O_5_	284, 256, 167, 133	RO, OV
Flavonols								
92	9.83	Rutin		611.1602	0.76	C_27_H_30_O_16_	465, 303, 287, 145, 129	RO
93	9.88	Quercetin-O-hexuronide	477.0661	479.0819	2.85 /0.24	C_21_H_18_O_13_	433, 343, 301, 113 /303, 285	RO, OV
94	10.30	Quercetin-O-hexoside (Isoquercetin)	463.0866	465.1027	3.45 /0.11	C_21_H_20_O_12_	301, 287, 175, 151 /303, 251, 145, 127	OV
95	10.31	Isorhamnetin-O-rutinoside	623.1388	625.1764	20 /-0.14	C_28_H_32_O_16_	477, 315 /479, 317	RO
96	11.06	Quercetin coumaroylhexoside	609.1235	611.1391	2.42 /0.71	C_30_H_26_O_14_	463, 301, 285 /465, 303, 287, 147	RO
97	11.97	Isorhamnetin	315.0506	317.0655	1.35 /0.25	C_16_H_12_O_7_	300, 271, 243 /302, 285, 121	RO, OV
98	12.87	Dimethylquercetin (Ombuin)	329.0660	331.0813	2.05 /-0.21	C_17_H_14_O_7_	314, 299, 271, 241, 199 /316, 121	RO, OV
Flavanones								
99	8.77	Gallocatechin	305.0693		-8.57	C_15_H_14_O_7_	225, 97, 59	RO, OV
100	10.48	Dihydroquercetin (Taxifolin)	303.0499	305.0655	3.7 /0.26	C_15_H_12_O_7_	285, 125, 109 /287, 259, 231, 153	OV
101	10.81	Hesperidin (Hesperetin-O-rutinoside)	609.1807		2.94	C_28_H_34_O_15_	301, 285, 251	RO
102	11.10	Aromadendrin	287.0553	289.0706	2.82 /0.22	C_15_H_12_O_6_	259, 243, 201, 177, 151, 125 /271, 243, 215, 153, 149	OV
103	11.77	Eriodictyol	287.0553	289.0706	2.82 /0.22	C_15_H_12_O_6_	151, 135, 125, 107 /271, 179, 163, 153	OV
104	12.49	Naringenin	271.0607	273.0751	1.83 /2.39	C_15_H_12_O_5_	253, 151, 119, 107 /255, 179, 153	OV
105	12.78	Hesperetin	301.0711	303.0854	2.19 /3.03	C_16_H_14_O_6_	286, 164, 151, 136 /177, 153, 137, 117	RO, OV
106	13.84	5,4’-Dihydroxy-6,7,8-trimethoxyflavanone	345.0970	347.1127	2.82 /-0.49	C_18_H_18_O_7_	330, 315, 301, 283, 119 /332, 227, 197	OV
107	14.26	Sakuranetin	285.0760	287.0915	2.96 /-0.35	C_16_H_14_O_5_	270, 165, 119 /167, 147, 119	OV
Monoterpenes								
108	8.45	Thymoquinol-O-dihexoside	535.2014		3.41	C_22_H_34_O_12_. CH_2_O_2_	489, 327, 165, 161, 101	OV
109	9.62	Thymoquinol-O-hexoside	373.1495		2.42	C_16_H_24_O_7_. CH_2_O_2_	327, 165, 164, 113, 101	OV
110	11.96	(+)-Camphor		153.1274	-0.06	C_10_H_16_O	109, 95, 81	RO, OV
111	12.98	Thymol		151.1117	0.28	C_10_H_14_O	123, 109, 91, 81	RO, OV
Sesquiterpenoids								
112	13.74	Spathulenol		221.1899	0.42	C_15_H_24_O	203, 147, 133, 119	OV
113	14.11	Caryophyllene epoxide		221.1900	-0.04	C_15_H_24_O	203, 147, 95	RO
114	18.11	Cubebene		205.1952	-0.6	C_15_H_24_	165, 148, 121, 93	OV
Diterpenes								
115	13.51	Carnosic acid hexoside	493.2430		2.64	C_26_H_38_O_9_	331, 287	RO
116	13.53	Hydroxy-O-methylrosmanol	375.1803		2.69	C_21_H_28_O_6_	357, 345, 331, 316, 299	RO, OV
117	14.09	Rosmanol derivative	677.3676		2.81	C_40_H_54_O_9_	345, 331, 315, 301, 283	RO
118	14.14	Hydroxyrosmanol	361.1647		2.66	C_20_H_26_O_6_	317, 299	RO, OV
119	14.69	Rosmanol derivative	689.3312		2.78	C_40_H_50_O_10_	555, 345, 283	RO
120	14.73	Sageone	299.1646	301.1799	2.23 /-0.26	C_19_H_24_O_3_	243, 200 /283, 259, 241, 231	RO, OV
121	14.75	Hydroxy-O-methylrosmanol derivative	719.3424		1.79	C_41_H_52_O_11_	375, 360, 345, 313, 299	RO, OV
122	14.88	Hydroxyrosmadial	359.1489	361.1643	3.09 /0.74	C_20_H_24_O_6_	341, 331, 315, 287/333, 317, 289, 287	RO, OV
123	14.99	Carnosol	329.1750	331.1892	2.52 /3.59	C_20_H_26_O_4_	314, 285, 270, 201 /313, 289, 271, 247	RO, OV
124	15.44	Oridonin	363.1804		2.5	C_20_H_28_O_6_	345, 333, 315, 297	RO
125	15.60	Salvinorin F	373.1648		2.3	C_21_H_26_O_6_	358, 343, 329, 314, 299	RO, OV
126	15.63	Rosmic acid	389.1592	391.1752	3.53 /-0.18	C_21_H_26_O_7_	345, 313, 301, 285 /331, 303, 285	RO, OV
127	15.81	Methylrosmanol	359.1857	361.2012	1.94 /-0.69	C_21_H_28_O_5_	329, 315, 283 /329, 301, 273, 109	RO, OV
128	16.11	Sageone derivative	629.3466		2.82	C_39_H_50_O_7_	329, 299, 285	RO, OV
129	16.40	Dimethoxyrosmanol	389.1956		3.49	C_22_H_30_O_6_	374, 344, 330, 313, 298	RO, OV
130	16.74	Rosmadial	343.1544	345.1697	2.03 /-0.14	C_20_H_24_O_5_	328, 315, 299 /317, 299, 271, 231	RO, OV
131	16.77	Rosmaridiphenol	315.1960	317.2094	1.8 /5.44	C_20_H_28_O_3_	285, 270, 201, 179 /299, 281, 191	RO, OV
132	16.92	Rosmanol	345.1700	347.1854	2.16 /-0.29	C_20_H_26_O_5_	327, 301, 283, 268, 227 /301, 259, 241	RO, OV
133	16.94	Tetrahydro-hydroxyrosmariquinone	301.1802		2.38	C_19_H_26_O_3_	283, 273, 258	RO, OV
134	17.23	O-Methylcarnosol	343.1907		2.27	C_21_H_28_O_4_	299, 284	RO, OV
135	17.83	Pisiferal		301.2163	-0.31	C_20_H_28_O_2_	259, 245, 231, 219, 205, 163	RO, OV
136	17.91	Demethylsalvicanol	317.2114		2.57	C_20_H_30_O_3_	299, 243, 191, 179	RO, OV
137	18.05	Carnosic acid	331.1904		3.26	C_20_H_28_O_4_	313, 287, 244, 151	RO, OV
138	18.45	O-methylcarnosic acid	345.2063	347.2193	2.41 /6.89	C_21_H_30_O_4_	301, 286, 271 /305, 273, 121, 109	RO, OV
139	18.79	Sugiol	299.2010		2.18	C_20_H_28_O_2_	283, 243, 227, 215	RO, OV
140	20.34	Rosmadial derivative	631.3989		2.39	C_40_H_56_O_6_	567, 343, 299	RO
141	21.49	Hydroxyrosmadial derivative	631.3989		2.39	C_40_H_56_O_6_	359, 331, 315	RO, OV
142	22.42	Rosmadial derivative	615.4032		3.73	C_40_H_56_O_5_	572, 343, 299	RO, OV
Triterpenes								
143	14.85	Asiatic acid	487.3416		2.66	C_30_H_48_O_5_	469, 453, 441, 409	RO
144	17.09	Pomolic acid	471.3466	473.3622	2.93/0.71	C_30_H_48_O_4_	453, 407 /455, 409, 191	RO, OV
145	17.36	Micromeric acid	453.3363	455.3518	2.46 /0.38	C_30_H_46_O_3_	409, 407, 392 /437, 409, 205	RO, OV
146	19.22	Oleanolic acid	455.3517		3	C_30_H_48_O_3_	409, 391	RO, OV
147	19.48	Ursolic acid	455.3519		2.56	C_30_H_48_O_3_	407, 315	RO, OV
148	19.51	3-oxours-12-en-20,28-olide	451.3206		2.58	C_30_H_44_O_3_	407, 389	RO
149	21.25	Corosolic acid	471.3466		2.93	C_30_H_48_O_4_	453, 443, 427, 409, 393	RO
Fatty acids derivatives								
150	12.69	Corchorifatty acid F	327.2170		2.13	C_18_H_32_O_5_	291, 229, 211, 171	RO, OV
151	13.29	Dihydroxyhexadecanoic acid	287.2221		2.37	C_16_H_32_O_4_	269, 241, 141, 99	RO, OV
152	14.48	Hydroxy-octadecatrienoic acid		295.2266	0.58	C_18_H_30_O_3_	277, 259, 133, 69	RO, OV
153	14.78	hydroperoxy-octadecadienoic acid	311.2220		2.51	C_18_H_32_O_4_	293, 223, 195	RO, OV
154	16.81	Linolenic Acid	277.2164	279.2320	3.25 /-0.52	C_18_H_30_O_2_	259, 233, 59 /123, 109, 95, 67	RO, OV
155	19.82	Hydroxystearic acid	299.2585		2.23	C_18_H_36_O_3_	253, 59	RO
156	21.09	Camaryolic acid	581.3833		2.51	C_36_H_54_O_6_	549, 497, 285	RO, OV
Others								
157	0.99	Quinic acid	191.0558		1.62	C_7_H_12_O_6_	173, 171, 127, 85	RO, OV
158	1.00	Acetyl-maltose	383.1189		1.56	C_14_H_24_O_12_	341, 191	RO
159	9.44	Hydroxybenzaldehyde	121.0293	123.0440	1.66 /0.46	C_7_H_6_O_2_	92, 65 /95, 77, 51	OV
160	10.25	Amburosides A	421.1127		3.13	C_20_H_22_O_10_	259, 153, 109	OV
161	10.82	Loliolide		197.1172	0.11	C_11_H_16_O_3_	179, 161, 133, 107	RO, OV
162	11.62	Dihydroxyanthraquinone carboxylic acid	283.0240		2.86	C_15_H_8_O_6_	239, 211, 135	OV
163	13.93	6-Oxocamphor	165.0917		2.43	C_10_H_14_O_2_	150, 135	OV
164	14.45	Hotrienol		153.1274	-0.06	C_10_H_16_O	135, 107, 97	RO, OV

#### Hydroxycinnamic acid derivatives

**Fig. 3:**
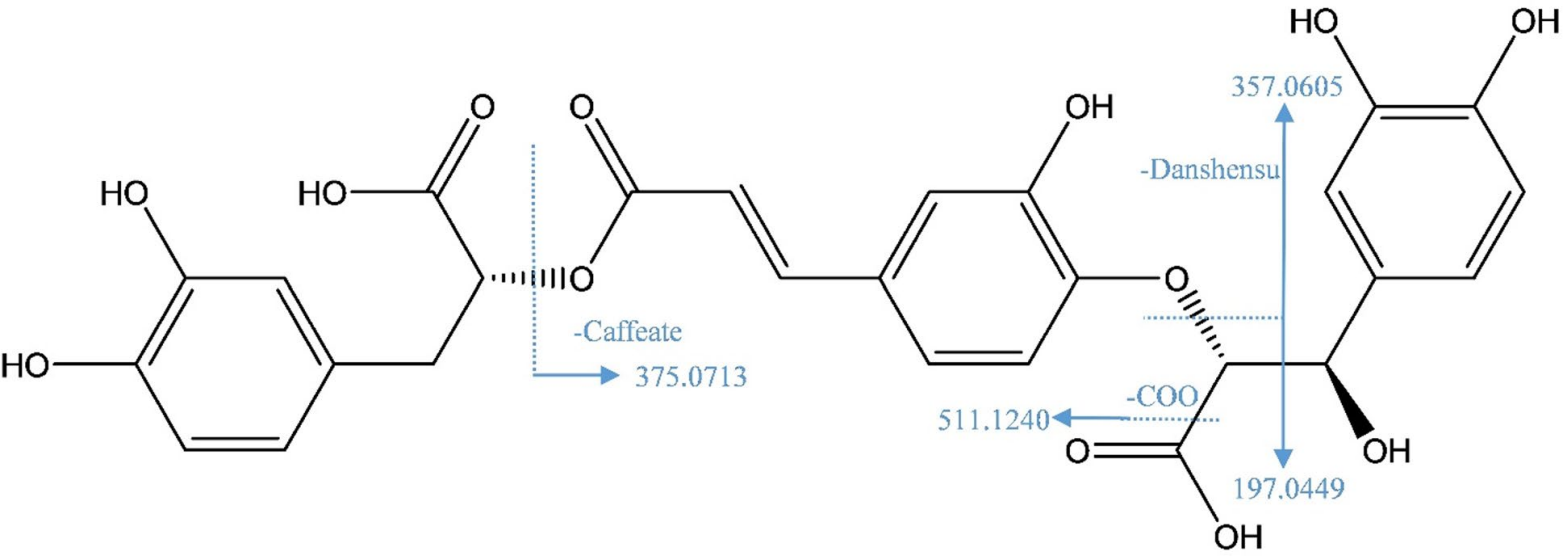
Fragmentation pattern of Salvianolic acid K.

Twenty-eight hydroxycinnamic acid derivatives of caffeic, coumaric, ferulic acids, and danshensu (salvianic acid A or α-hydroxy dihydrocaffeic acid C_9_H_10_O_5_) were detected. These derivatives showed common major fragment ions at *m/z* 197^−^ for deprotonated danshensu, *m/z* 179^−^ for caffeate, *m/z* 161^−^ for caffeoyl, and *m/z* 135^−^ for decarboxylated caffeate in MS^2^ spectra (Fig. 3).

Depsides, i.e., hydroxycinnamic acid dimers or oligomers linked *via* ester bonds, are characteristic chemomarkers of the Lamiaceae family^28^. They are recognized as bioactive phenolics for their antioxidant, antiallergic, immunomodulatory, and antimicrobial activities^29^. Twelve depsides were identified herein, with salvianolic acid D (cpd. **7**) detected for the first time in RO, and both salvianolic acid K (cpd. **15**) and cleroden J (cpd. **33**) in *O. vulgare*.

Salvianolic acid D and rosmarinic acid are caffeic acid dimers. Salvianolic acid D (cpd. **7**, *m/z* 417.0791 [M-H]^−^, [C_20_H_17_O_10_]^−^) showed a base peak in MS^2^ spectra at *m/z* 219 [M-H-198]^−^ signifying the loss of a danshensu moiety (Supplementary Fig. 1S)^30^. Rosmarinic acid-*O*-hexoside (cpd. **16**, *m/z* 521.1285 [M-H]^−^, [C_24_H_25_O_13_]^−^), rosmarinic acid (cpd. **18**, *m/z* 359.0766 [M-H]^−^, [C_18_H_15_O_8_]^−^) and methylrosmarinic acid (cpd. **30**, *m/z* 373.0917 [M-H]^−^, [C_19_H_17_O_8_]^−^) shared the same fragment ions at *m/z* 197 and 179 in MS^2^ spectra, corresponding to deprotonated danshensu and caffeate moieties, respectively (Supplementary Fig. 2–4 S)^31^.

Cleroden J (cpd. **33**, *m/z* 553.1339 [ M-H]^−^, [C_28_H_25_O_12_]^−^) was detected in *O. vulgare* L. for the first time and has previously been isolated from other species within the same family^32^. The spectrum was dominated by a base peak at *m/z* 135, corresponding to the decarboxylated caffeic acid fragment [caffeic acid–H–CO_2_]^−^. Additional product ions were observed at *m/z* 521 arising from the loss of a methoxy group [M–H–OCH_3_]^−^, and at *m/z* 477, corresponding to subsequent decarboxylation [M–H–OCH_3_–CO_2_]^−^. A further significant ion at *m/z* 179 [C_9_​H_7_​O_4_]^−^ confirms the presence of caffeic acid units within the structure (Supplementary Fig. 5S).

Caffeic acid trimers *vis.* salvianolic acids A and K were identified. Salvianolic acid K (cpd. **15**, *m/z* 555.1129 [M-H]^−^, [C_27_H_23_O_13_]^−^) revealed daughter ions at *m/z* 511 [M-H-44]^−^ and 357 [M-H-198]^−^ after the losses of carboxyl group and danshensu moieties (Supplementary Fig. 6S), respectively^33^. The fragmentation pattern of this compound is presented as a representative example of the.

Salvianolic acid B and sagerinic acid are caffeic acid tetramers (rosmarinic acid dimers). Salvianolic acid B1 is formed by oxidative cyclization of two rosmarinic acid molecules, giving a 1,2-dihydronaphthalene ring structure, while sagerinic acid is formed by dimerization of 2 rosmarinic acid molecules and cyclobutane ring formation^34^. Salvianolic acid B (cpd. **20**, *m/z* 717.1437 [M-H]^−^, [C_36_H_29_O_16_]^−^), produced a base peak ion at *m/z* 359 [C_18_H_15_O_8_]^−^ representing rosmarinic acid, and the less abundant product ions that indicate sequential losses of two danshensu: at *m/z* 519 [M-H-198]^−^, and *m/z* 321 [M-H-198-198]^−^ (Supplementary Fig. 8S)^35^.

Sagerinic acid (cpd. **21**, *m/z* 719.1602 [M-H]^−^, [C_36_H_31_O_16_]^−^ ) generated a base peak fragment ion at *m/z* 359 [M-H-360]^−^, resulting from molecular splitting [M/2]^−^, corresponding to rosmarinic acid and another ion with lower intensity at *m/z* 197 [C_9_H_9_O_5_]^−^ corresponding to the danshensu moiety (Supplementary Fig. 9S)^34^.

#### Flavonoids

Flavonoids comprised the major class of secondary metabolites in the alcoholic profiles of both *S. rosmarinus* Spenn. and *O. vulgare* L., where a total of 56 flavonoid derivatives were detected in this study. These included representatives of flavanones, flavonols, and flavones, among which five compounds in *O. vulgare* L. (nepitrin; cpd. **60**, hispidulin-*O*-rutinoside; cpd. **63**, luteolin-*O*-pentosyl-acetyl-hexoside; cpd. **64**, luteolin caffeoylhexoside; cpd. **67**, and acacetin-*O*-rutinoside; cpd. **71**) and one compound in *S. rosmarinus* Spenn. (quercetin coumaroylhexoside; cpd. **96**) were identified for the first time in these species. The elution of flavonoids appeared to follow a decreasing polarity sequence in chromatograms. So firstly, at the R_t_ range (9.44–11.92 min.), flavonoid diglycosides were detected. Apigenin-6,8-di-*C*-hexoside (cpd. **52**, *m/z* 593.1491 [M-H]^−^, [C_27_H_29_O_15_]^−^) was annotated after showing the losses of 120 amu (*m/z* 473) and 90 amu (*m/z* 503), indicative of a *C*-linked hexoside internal cleavage, and the losses of 240 (-2 × 120) amu (*m/z* 353) and 210 (-120-90) amu (*m/z* 383) that also indicate the internal cleavages that occur in both *C*-linked hexoside (Supplementary Fig. 10S)^36^. Rutin, quercetin-*O*-rutinoside, (cpd. **92**, *m/z* 611.1602 [M + H]^+^, [C_27_H_31_O_16_]^+^) was confirmed by the MS^2^ spectra, which displayed fragment ions at *m/z* 465 and *m/z* 303, denoting the loss of a deoxyhexose sugar [M + H-146]^+^ followed by the loss of a hexose sugar [M + H-146-162]^+^, respectively (Supplementary Fig. 11S)^37^. Luteolin-*O*-rutinoside (cpd. **56**, *m/z* 593.1495[M-H]^−^, [C_27_H_29_O_15_]^−^) yielded a characteristic base peak of *m/z* 285 for the luteolin fragment after the loss of 308 (-162-146) amu of the rutinoside moiety (Supplementary Fig. 12S) Luteolin-*O*-pentosyl-hexoside (cpd. **61**, *m/z* 581.1498 [M + H]^+^, [C_26_H_29_O_15_]^+^), showed a base peak ion in the MS^2^ spectra at *m/z* 287 [M + H-162-132]^+^, suggesting the respective losses of hexose and pentose (Supplementary Fig. 13S). Apigenin-*O*-rutinoside (cpd. **62**, *m/z* 579.1693 [M + H]^+^, [C_27_H_31_O_14_]^+^) represented major fragment ions at *m/z* 433 [M + H-146]^+^ and 271 [M + H-146-162]^+^, indicating the loss of a deoxyhexose sugar followed by a hexose sugar, respectively (Supplementary Fig. 14S)^38^.

Flavonoid monoglycosides were identified in the following section of the chromatographic elution at R_t_ range of 9.85–12.08 min. Nepitrin (6-methoxyluteolin-*O*-hexoside) (cpd. **60**, *m/z* 479.1183 [M + H]^+^, [C_22_H_23_O_12_]^+^) displayed a prominent product ion in MS^2^ at *m/z* 317 appeared after the loss of a hexose [M + H-162]^+^ moiety and another fragment at *m/z* 302 appeared after the losses of a hexose and methyl group [M + H-162-15]^+^ (Supplementary Fig. 15S). Apigenin-*O*-hexoside (cpd. **65**, *m/z* 433.1128 [M + H]^+^, [C_21_H_21_O_10_]^+^) and apigenin-*O*-hexuronide (cpd. **66**, *m/z* 447.0921 [M + H]^+^, [C_21_H_19_O_11_]^+^) are monoglycoside flavones, both demonstrated a similar base peak ion at *m/z* 271 [C_15_H_11_O_5_]^+^, corresponding to apigenin aglycone after the losses of hexose and hexuronide moieties, respectively, and minor daughter ions at *m/z* 153 and 109 resulting from Retro Diels Alder (RDA) reactions (Supplementary Fig. 16S, 17 S). Homoplantaginin (Hispidulin-*O*-hexoside) (cpd. **75**, *m/z* 463.1235 [M + H]^+^, [C_22_H_23_O_11_]^+^ ) showed a base peak ion at *m/z* 301 [M + H-162]^+^ after the loss of a hexose moiety and another fragment ion at *m/z* 286 [M + H-162-15]^+^ was produced due to the loss of a hexose followed by methyl moiety, that matched characteristic fragments of hispidulin (Supplementary Fig. 18S).

Flavonoid aglycones were detected in the chromatograms after R_t_ (10.48 min.). Eriodictyol (cpd. **103**, *m/z* 287.0553 [M-H]^−^, [C_15_H_11_O_6_]^−^) gave fragment ions resulting from aglycone C-ring RDA cleavages, showing a base peak ion at *m/z* 135 (^1,3^B^−^), a major fragment ion at *m/z* 151 (^1,3^A^−^) and minor fragment ions at *m/z* 107 and *m/z* 125 for (^1,3^A^−^-CO_2_) and (^1,4^A^−^), respectively (Supplementary Fig. 19S)^39^ Similarly, apigenin aglycone (cpd. **77**, *m/z* 269.0451 [M-H]^−^, [C_15_H_9_O_5_]^−^), demonstrated the MS^2^ data that illustrated the RDA fragment ions at *m/z* 151 (^1,3^A^−^ ), *m/z* 149 (^1,4^B^−^+2H), *m/z* 117 (^1,3^B^−^) and 107 (^1,3^A^−^-CO_2_) due to C-ring cleavage in addition to other fragment ions resulting from small losses *vis. m/z* 225 and *m/z* 201 consecutive to CO_2_ and C_3_O_2_ losses (Supplementary Fig. 20S)^38,39^. Isorhamnetin; *O*-methyl-quercetin, (cpd. **97**, *m/z* 315.0506 [M-H]^−^, [C_16_H_11_O_7_]^−^) was identified based on the distinctive MS^2^, where the loss of a methyl group (30 amu) was noted to produce a base peak ion at *m/z* 300 and minor fragment ions at *m/z* 271 and *m/z* 243 corresponding to the loss of CO_2_ and CO_2_ + CO groups, respectively (Supplementary Fig. 21S)^40^. Hydroxygenkwanin (cpd. **82**, *m/z* 299.0554 [M-H]^−^, [C_16_H_11_O_6_]^−^) was suggested as on dissociation produce fragment ions at *m/z* 284 [M-H-15]^−^ denoting the loss of a methyl, *m/z* 256 [M-H-15-28]^−^ indicating the losses of methyl and CO groups, *m/z* 227 [M-H-15-28-29]^−^ inferring the losses of methyl, CO and CHO groups and *m/z* 151 (^1,3^A^−^-CH_3_) and *m/z* 133 (^1,3^B^−^) due to RDA cleavage of C-ring (Supplementary Fig. 22S)^41^. Sakuranetin (cpd. **107**, *m/z* 285.0760 [M-H]^−^, [C_16_H_13_O_5_]^−^) had a characteristic MS^2^ showing major fragment ions at *m/z* 165 and 119 resulting from RDA cleavage of the C-ring at (^1,3^A^−^) and (^1,3^B^−^), respectively (Supplementary Fig. 23S)^7^. Salvigenin (cpd. **90**, *m/z* 329.1019 [M + H]^+^, [C_18_H_17_O_6_]^+^) showed fragment ions at *m/z* 314 [M + H-15]^+^ corresponding to the loss of a methyl group, *m/z* 296 [M + H-15-18]^+^ indicating the a further loss of water molecule and a minor fragment ion at *m/z* 268 [M + H-15-18-28]^+^ due to the collective loss of methyl, water and CO groups (Supplementary Fig. 24S)^42,43^.

#### Terpenes

A total of 42 metabolites belonging to monoterpenes, diterpenes, sesquiterpenoids, and triterpenes were detected and mainly eluted at the middle and late sections of the chromatogram (R_t_ =8.4–22.4 min.). Abietenes are phenolic diterpenes of limited distribution in some species of the Lamiaceae family^44^. These compounds have various biological actions, including antioxidant, anti-inflammatory, and anti-microbial properties^45^. Hydroxy-*O*-methylrosmanol (cpd. **116**, *m/z* 375.1803 [M-H]^−^, [C_21_H_27_O_6_]^−^) generated a major fragment ion at *m/z* 299 [M-H-44-31-1]^−^ after the losses of CO_2_ and OCH_3_ and molecular rearrangement, besides the daughter ions at *m/z* 345 [M-H-30]^−^ [C_20_H_25_O_5_]^−^ for the rosmanol fragment, in addition to *m/z* 331 [M-H-44]^−^ and *m/z* 316 [M-H-44-15]^−^ attributed to the losses of CO_2_ and CH_3,_ respectively (Supplementary Fig. 25S)^46^. Rosmadial (cpd. **130**, *m/z* 343.1544 [M-H]^−^, [C_20_H_23_O_5_]^−^) gave fragments at *m/z* 315 [M-H-28]^−^ for a loss of CO, and *m/z* 299 [M-H-44]^−^ suggesting a cleavage of CO_2_ (Supplementary Fig. 26S)^47^. Tetrahydro-hydroxyrosmariquinone (cpd. **133**, *m/z* 301.1802 [M-H]^−^, [C_19_H_25_O_3_]^−^) was assigned after revealing product ions; *m/z* 283 [M-H-18]^−^, 273 [M-H-28]^−^, and 258 [M-H-43]^−^ following the losses of H_2_O, CO, and (CH(CH_3_)_2_), respectively (Supplementary Fig. 27S) and in accordance with previous reported data^47^. Carnosic acid (cpd. **137**, *m/z* 331.1904 [M-H]^−^, [C_20_H_27_O_4_]^−^) revealed a major fragment ion at *m/z* 287 [M-H-44]^−^ after decarboxylation that was followed by the loss of an isopropyl group (CH(CH_3_)_2_) yielding a fragment ion at *m/z* 244 [M-H-44-43]^−^ (Supplementary Fig. 28S). *O*-methylcarnosic acid (cpd. **138**, *m/z* 345.2063 [M-H]^−^, [C_21_H_29_O_4_]^−^) on fragmentation showed two major ions at *m/z* 301 and *m/z* 286, corresponding to the losses of CO_2_ [M-H-44]^−^ and a successive loss of methyl group [M-H-44-15]^−^, and fragment ion with low intensity at *m/z* 271 resulted from the further loss of a methyl group [M-H-44-15-15]^−^ (Supplementary Fig. 29S)^48^.

Six pentacyclic triterpenes were identified in alcoholic extracts of *S. rosmarinus* Spenn. and *O. vulgare* L., among them oleanolic acid, ursolic acid, and corosolic acid, which possess documented anti-inflammatory and antioxidant properties^49^. Asiatic acid (cpd. **143**, *m/z* 487.3416 [M-H]^−^, [C_30_H_47_O_5_]^−^) showed characteristic fragment ions in its MS^2^ spectra at *m/z* 469 [M-H-18]^−^ after dehydration, *m/z* 441 [M-H-46]^−^ corresponding to the loss of HCOOH, and *m/z* 409 [M-H-46-32]^−^ by the respective losses of HCOOH and CH_3_OH (Supplementary Fig. 30S).

### Antibacterial activity

#### Evaluation of minimum inhibitory concentrations (MIC) and biofilm formation Inhibition

The extracts exhibited variable antibacterial activities against MRSA and *E. coli* Table 2. The methanolic extract of *S. rosmarinus* Spenn. and the aqueous extract of *O. vulgare* L. exhibited the strongest antibacterial activities (MIC = 64 µg/mL against both strains).

**Table 2:** MIC inhibitory test of methanolic And aqueous extracts of *S. rosmarinus* Spenn. And *O. vulgare* L. against MRSA And *E. coli*.

Extract	MRSA (µg/mL)	*E. coli *(µg/mL)
RO	64	64
Aq.RO	256	128
OV	256	128
Aq.OV	64	64
Doxycycline	1	32

*MIC* minimum inhibitory concentration, *MRSA* methicillin-resistant *Staphylococcus aureus*,* E. coli*; *Escherichia coli.* MICs were assayed in triplicate.

Biofilm development presents a significant challenge in microbial infections because of its role in increasing resistance. Therefore, the most active extracts were tested for their ability to inhibit biofilm formation in MRSA. In our study, RO and Aq.OV, which showed the strongest antibacterial activity, were further examined for their antibiofilm formation activity. RO and Aq.OV extracts showed significant inhibition at ¼ MIC, reducing biofilm formation by 54.9 ± 2.3% and 61.4 ± 0.6%, respectively, and an unpaired t-test confirmed a statistically significant difference between them (*p* = 0.0091). Based on established criteria^50^, a plant extract achieving a biofilm inhibition level greater than 50% is generally regarded as indicative of good antibiofilm activity. Accordingly, the inhibition levels observed in our study indicate that these extracts may represent promising alternatives to conventional antibiotics for managing MRSA infections, especially in biofilm-associated cases where standard therapies often fail due to the protective biofilm^51^.

#### Reduction of *IcaA* and *AgrA* gene expression levels

**Fig. 4:**
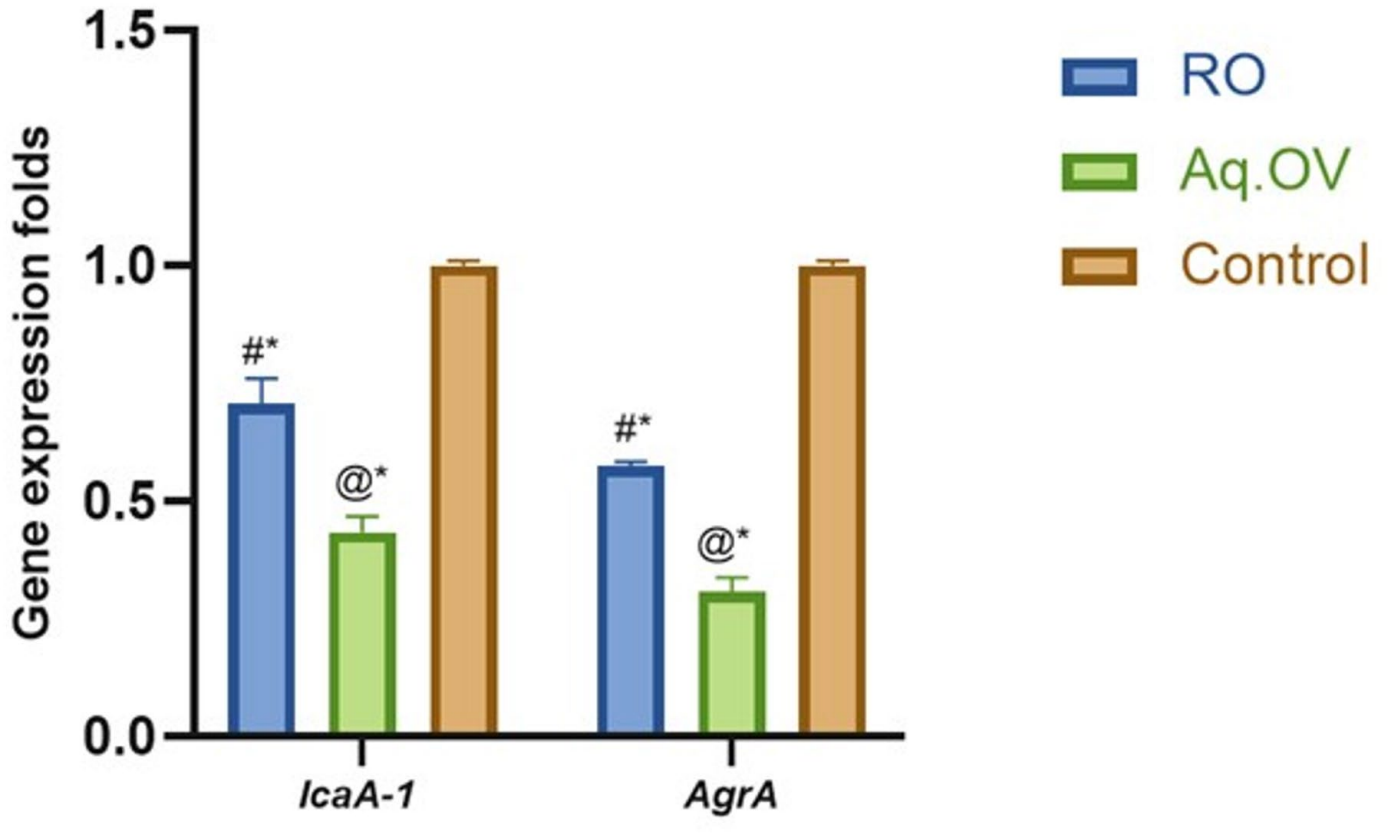
Inhibition of *icaA-1* gene responsible for biofilm formation and *agrA* gene accountable for quorum sensing in MRSA. RO; *Salvia* r*osmarinus*, Aq.OV; Aqueous extract of *Origanum vulgare*. Data are represented as the mean ± SD of three independent assays. Statistical analysis was performed using one-way ANOVA, followed by Tukey’s post hoc test was performed to calculate the statistical significance (*p* < 0.0001). As compared to RO (@), Aq.OV (#), and control (*).

The *icaA* and *agrA* are important genes that play crucial roles in virulence and biofilm development in MRSA; the *icaA* gene is responsible for biofilm formation and the *agrA* gene is responsible for quorum sensing of MRSA^52^. The *icaA* was downregulated by 30% and 60% after treatment of MRSA with ¼ MIC of RO and Aq.OV extracts, respectively. Similarly, the *agrA* gene was also downregulated by 43% and 70% after treating MRSA with ¼ MIC of RO and Aq.OV extracts, respectively (Fig. 4). For both *icaA-1* and *agrA* genes, Tukey’s multiple comparisons test demonstrated statistically significant differences among all groups (RO extract, Aq.OV extract, and control) (*p* < 0.0001). These results are consistent with the observed antibiofilm activity, indicating that the extracts interfere with critical genetic regulators essential for biofilm formation.

### Antioxidant activity

The antioxidant activity was assessed using complementary in vitro assays based on different mechanisms, including the DPPH radical scavenging assay and the FRAP reducing power assay.

The results revealed that the methanolic extract of *S. rosmarinus* Spenn. exhibited a significantly stronger radical scavenging capacity in the DPPH assay (IC_50_ = 6.56 ± 0.035 µg/mL) compared to the methanolic extract of *O. vulgare* L. (IC_50_ = 17.60 ± 0.333 µg/mL (Unpaired Student’s *t*-test, *p* < 0.0001) (Table 3). In contrast, *O. vulgare* L. exhibited a significantly higher reducing capacity in the FRAP assay (188.61 ± 24.06 µmol/L) compared to *S. rosmarinus* Spenn. (122.78 ± 2.09 µmol/L) (Unpaired Student’s *t*-test, *p* = 0.0004) (Table 3).

**Table 3:** Antioxidant activity of methanolic extracts of *S. rosmarinus* Spenn. And *O. vulgare* L. using 2,2-diphenyl-1-picrylhydrazyl (DPPH) And ferric-reducing antioxidant power (FRAP) assays.

Antioxidant activity		
	RO	OV
DPPH “IC_50_” (µg/mL)	6.56 ± 0.035^*^	17.60 ± 0.333
FRAP (µmol/L)	122.78 ± 2.092	188.60 ± 10.058^#^

The results are presented as means ± SD of three measurements (*n* = 3). Statistical analysis was performed using the Unpaired Student’s *t*-test. * (*p* < 0.0001) and # (*p* = 0.0004).

### Anti-inflammatory activity

Inflammation is a complex biological response that serves as both an indicator of pathological disturbances and a contributor to disease progression as a result of sustained disruption of inflammatory regulation^53^. Pro-inflammatory cytokines, such as TNF-α, are released in response to inflammatory stimuli, which activate NF-κB signaling and subsequently induce the expression of COX-II^53^. In this study, the anti-inflammatory activity of the methanolic extracts of *S. rosmarinus* Spenn. and *O. vulgare* L. was evaluated through COX-II inhibition, as well as the suppression of TNF-α and NF-κB, using ibuprofen as a standard. *S. rosmarinus* Spenn. extract demonstrated strong anti-inflammatory potential, as evidenced by its COX-II inhibitory effect (IC_50_ = 11.09 ± 0.46 µg/mL), which was comparable to ibuprofen (IC_50_ = 8.79 ± 0.37 µg/mL), with no statistically significant difference between them (Tukey’s post hoc test, *p* < 0.595). In parallel, *S. rosmarinus* Spenn. extract significantly suppressed key inflammatory mediators, reducing TNF-α and NF-κB levels to 0.385 and 0.31 fold, respectively, of the control values that were close to those achieved by ibuprofen, with significant differences observed for TNF-α (*p* = 0.0013) and NF-κB (*p* = 0.0102). Conversely, *O. vulgare* L. extract exhibited noticeably weaker anti-inflammatory effects across all tested markers, showing significantly lower efficacy than ibuprofen (*p* < 0.0001) (Table 4).

**Table 4:** Anti-inflammatory activity of *S. rosmarinus* Spenn. And *O. vulgare* L. methanolic extracts.

	COX-II IC_50_ (µg/mL)	TNF-α (pg/mL)	NFқb (pg/mL)
RO	11.09 ± 0.46 ^NS^	536.6 ± 13.4 ^**^	616.2 ± 26.9^*^
OV	34.73 ± 1.44 ^****^	874.1 ± 35^****^	1025 ± 40.4^****^
Ibuprofen	8.791 ± 0.37	409.4 ± 14.7	496.2 ± 24.7
Control	--	1392 ± 21.3	1992 ± 48.7

Results are presented as (mean ± SD) of three measurements (*n* = 3). Statistical analysis was performed using one-way ANOVA, followed by Tukey’s post hoc test, which was performed to calculate the statistical significance relative to the ibuprofen group indicated as follows: (*) at *p* < 0.05; (**) at *p* < 0.005; (****) at *p* < 0.0001; NS = not significant.

### Multivariate data analysis and Pearson’s correlation study

The PLS model, as a supervised approach, was adopted to determine the relationship between the identified metabolites (Table 1) and the results of the biological investigations of *S. rosmarinus* Spenn. and *O. vulgare* L. extracts, utilizing the UPLC–QTOF–MS/MS dataset as X variables and the Y variables were the antibacterial activity represented as 1/MIC values against *E. coli* and MRSA strains, the antioxidant activity represented as 1/IC_50_ values of DPPH radical scavenging and the values of the ferric ion reducing capacity of the extract (FRAP), and the anti-inflammatory activity represented by the down-regulation levels of the inflammation biomarkers calculated as 1/COX-II, 1/NF-*к*B, and 1/TNF-*α*.

**Fig. 5:**
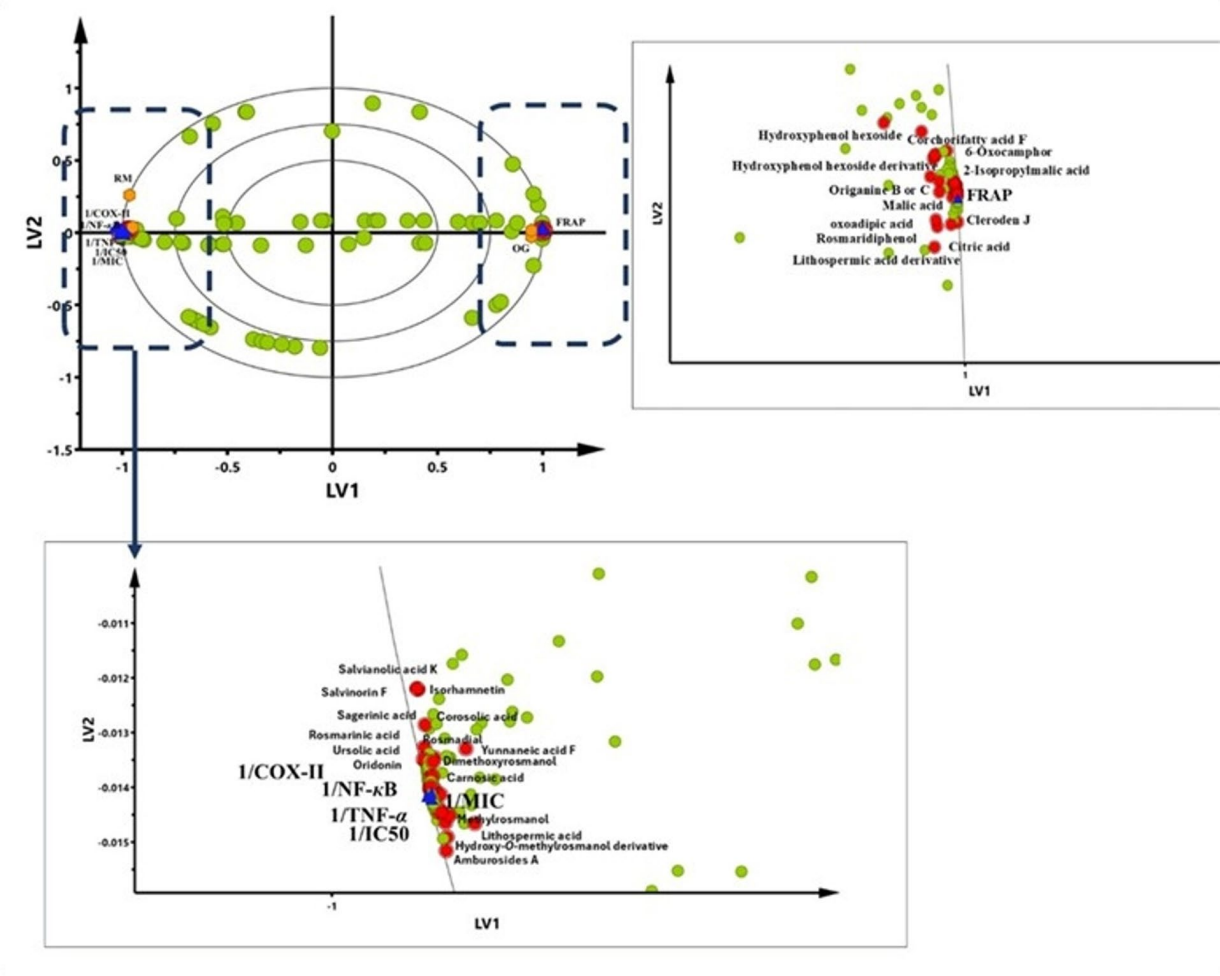
PLS scores-loadings biplot describing the correlations of the identified metabolites in rosemary; RO and oregano; OV extracts and their studied bioactivities. In zoom, the detected compounds are annotated as metabolites.

The PLS Model was validated by the quality of fitness and prediction of Y. The autofit of the PLS model demonstrated excellent fit (R^2^_Y_ cum = 0.996) and predictive capability (Q^2^ cum = 1), suggestive of a strong model with no overfit. The PLS biplot, which combines score and loading charts, was used to visually represent the relation between the samples and variables contributing to differentiation across the extracts (Fig. 5). The proximity of the X and Y variables to the sample clusters signifies their degree of contribution to the defining traits of each cluster. Figure 5 illustrates that the *S. rosmarinus* Spenn. extract was segregated from the *O. vulgare* L. extract by the first latent variable (LV1). This may be attributed to differences in metabolite abundances.

Moreover, the bacterial inhibition activity and the DPPH free radical scavenging activity were positioned alongside the *S. rosmarinus* Spenn. extract, signifying their superior activities relative to the *O. vulgare* L. extract. Notably, diterpenes (i.e., oridonin, carnosic acid, hydroxyrosmanol, rosmic acid, dimethoxyrosmanol, rosmadial, and tetrahydro-7-hydroxyrosmariquinone), triterpenes (i.e., asiatic acid, corosolic acid, and 3-oxours-12-en-20,28-olide), hydroxycinnamic acids (i.e., yunnaneic acid F, salvianolic acid K, and rosmarinic acid), and flavonoid derivatives (i.e., nepitrin, isorhamnetin, and hesperetin) were found to be enriched in the *S. rosmarinus* Spenn. extract. This demonstrates their beneficial effects on the DPPH free radical scavenging and bacterial inhibitory activities in *S. rosmarinus* Spenn. extract. The strong antibacterial and DPPH free radical scavenging actions of these metabolites can be attributed to the existence and quantity of phenolic hydroxyl groups^54^. In contrast, the *O. vulgare* L. extract had minimal projection towards those Y variables, positioned on the positive side of the latent variable (LV1). This observation indicated a lesser correlation of the *O. vulgare* L. extract with the examined bioactivities. These findings are aligned with results from the evaluation of the antibacterial activity study and evaluation of the free radical scavenging activity (DPPH assay), where *S. rosmarinus* Spenn. extract showed stronger bacterial inhibition than *O. vulgare* L. extract (Table 2). Also, *S. rosmarinus* Spenn. extract exhibited the highest scavenging capacity of DPPH^**·**^ radical with the lowest IC_50_ value, followed by *O. vulgare* L. extract, indicating strong antioxidant activity of the extract (Table 3).

However, antioxidant activity investigated as ferric ion reducing capacity (FRAP) was positioned alongside the *O. vulgare* L. extract, signifying their superior activities relative to *S. rosmarinus* Spenn. extract. These findings indicated that *O. vulgare* L. extract exhibited an antioxidant activity due to their enrichment in benzyl derivatives (i.e., hydroxyphenol hexoside, hydroxyphenol hexoside derivative, and origanine B or C), hydroxycinnamic acids (i.e., lithospermic acid derivative), and organic acids and esters (i.e., oxoadipic acid, corchorifatty acid F, malic acid, and citric acid) were found to be enriched in the *O. vulgare* L. extract. The enrichment of these compounds may account for their notable ferric ion-reducing capacity and overall antioxidant activity. These constituents are known for their redox properties, free radical scavenging ability, and metal ion chelation, which collectively contribute to the observed antioxidant potential^8^. Regarding the anti-inflammatory activity of both extracts assessed by inhibiting COX-II activity and modulating NF-*к*B, and TNF-*α* pathways, those Y variables showed proximity to the extract of *S. rosmarinus* Spenn., indicating a direct correlation. *O. vulgare* L. exhibited less projection, suggesting less correlation. *S. rosmarinus* Spenn. extract is richer in flavonoid derivatives (i.e., luteolin-acetyl-*O*-hexuronide, apigenin, dimethylquercetin, and cirsimaritin), hydroxycinnamic acids (i.e., salvianolic acid B and lithospermic acid), and diterpenes (i.e., rosmanol, hydroxy-*O*-methylrosmanol, hydroxyrosmadial, and carnosol), which are well recognized for their anti-inflammatory effects^55^. These findings are consistent with the anti-inflammatory activity of the extracts, where the *S. rosmarinus* Spenn. extract showed stronger anti-inflammatory activity than the *O. vulgare* L. extract (Table 4). Previous reports highlighted the importance of flavonoid derivatives and diterpenes as anti-inflammatory metabolites. For instance, carnosic acid and carnosol exert anti-inflammatory effects primarily by inhibiting the NF-κB, MAPK, STAT3, and NLRP3 inflammasome pathways, leading to reduced expression of pro-inflammatory cytokines such as TNF-α, IL-1β, and IL-6. They also activate SIRT1, which further suppresses inflammation by downregulating these signaling cascades^56^. Additionally, flavonoids such as luteolin and apigenin have been proven to exhibit anti-inflammatory effects mainly by inhibiting the transcriptional activity of NF-κB without affecting its upstream signaling. They also slightly reduce JNK activation and suppress the expression of pro-inflammatory chemokines, contributing to their overall anti-inflammatory action^57^.

**Fig. 6:**
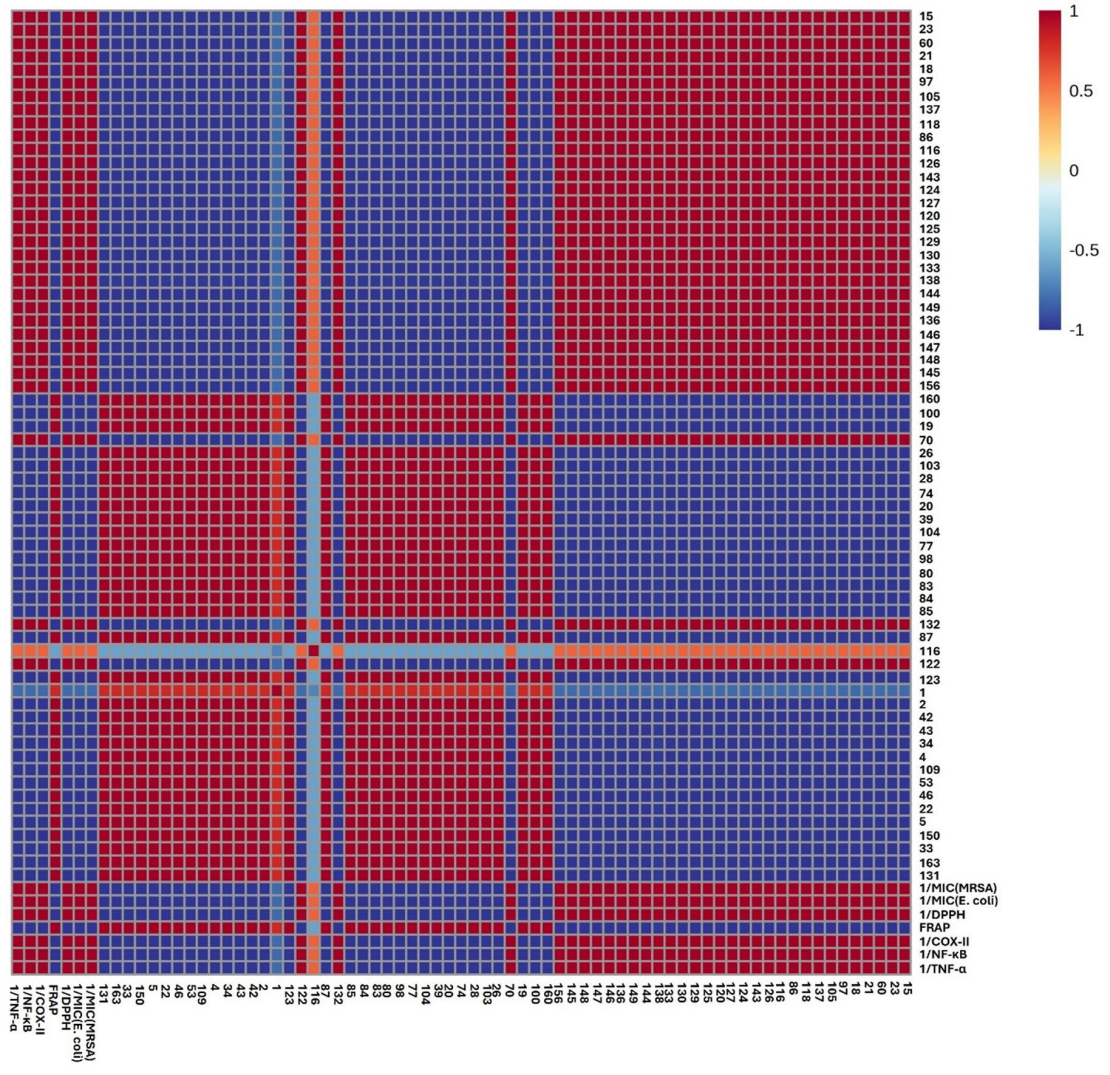
Pearson’s correlation between the metabolites and the antibacterial, antioxidant, and anti-inflammatory activities. Intensity of colors (blue and red) indicates correlation coefficients. The numbers listed on both axes refer to the identified metabolites listed in Table 1.

**Fig. 7:**
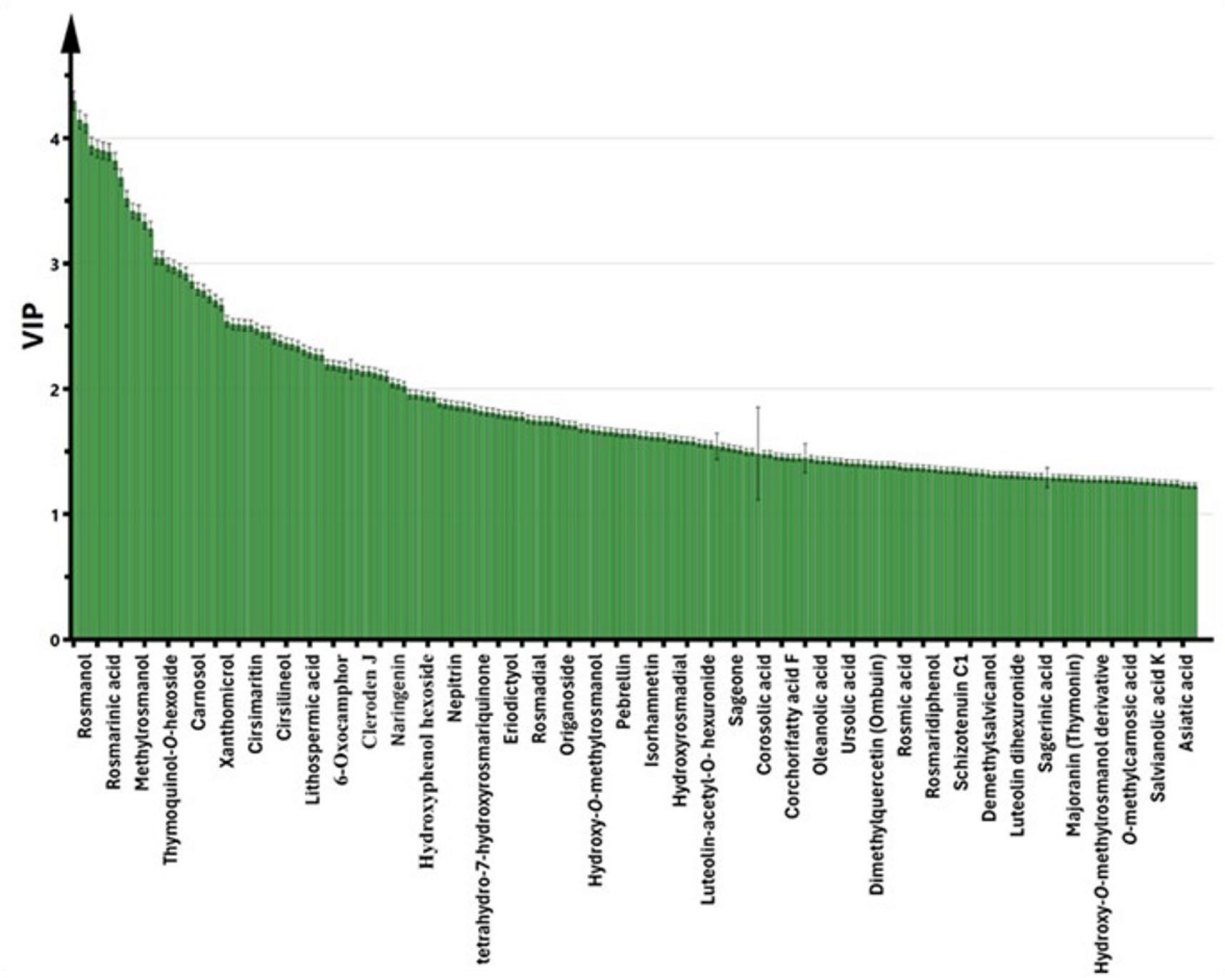
Variable Importance in the Projection (VIP) plot of the PLS model for the top contributing metabolites to bioactivities (VIP ≥ 1).

The correlation analysis was confirmed by calculating Pearson’s correlation coefficient (*r* ≥ 0.7), with statistical significance set at *p* < 0.05 and a false discovery rate (FDR) < 0.08. A correlogram (Fig. 6) was generated to visualize the strength of correlations among the variables. Notably, the analysis focused on metabolites with a PLS variable importance in projection (VIP) score of 1 or higher (Fig. 7), leading to the selection of 66 annotated metabolites (Fig. 6). The correlogram revealed strong positive correlations between the examined anti-microbial activity, DPPH radical scavenging activity, and hydroxycinnamic acid derivatives, diterpenes, triterpenes, and flavonoid derivatives, mainly present in *S. rosmarinus* Spenn. extract, namely, oridonin, carnosic acid, hydroxyrosmanol, rosmic acid, dimethoxyrosmanol, rosmadial, tetrahydro-7-hydroxyrosmariquinone, asiatic acid, corosolic acid, 3-oxours-12-en-20,28-olide, Yunnaneic acid F, salvianolic acid K, rosmarinic acid, nepitrin, isorhamnetin, and hesperetin. The antimicrobial activities of carnosic acid and rosmarinic acid are well-documented in the literature and appear to involve multiple complementary mechanisms. Carnosic acid has been reported to act as a potential quorum-sensing inhibitor, thereby suppressing bacterial virulence and biofilm formation. Additionally, its lipophilic nature allows it to incorporate into bacterial membranes, leading to membrane destabilization, increased permeability, and potential cell lysis^58^. Similarly, rosmarinic acid exerts its antibacterial effects primarily through membrane disruption, inhibition of efflux pumps, interference with essential bacterial enzymes, and suppression of biofilm formation, with particularly pronounced activity against Gram-positive bacteria. These mechanisms collectively support the strong antimicrobial potential of these phenolic compounds, particularly in targeting persistent and resistant bacterial strains^59^. Additionally, benzyl derivatives, organic acids & esters, and hydroxycinnamic acids are mainly present in *O. vulgare* L. extract exhibited strong positive correlations with the antioxidant activity investigated as FRAP, including hydroxyphenol hexoside, hydroxyphenol hexoside derivative, origanine B or C, lithospermic acid derivative, cleroden J, oxoadipic acid, and corchorifatty acid F. Lithospermic acid has been proven to possess significant antioxidant activity, primarily through its ability to scavenge free radicals. By neutralizing reactive oxygen species (ROS), it plays a crucial role in protecting cellular components from oxidative damage. These documented mechanisms highlight its potential as a therapeutic agent against oxidative stress-related diseases, reinforcing its value as a key natural antioxidant in plant-based systems^60^. Also, the investigated anti-inflammatory activities were strongly positively correlated with flavonoid derivatives, diterpenes, and hydroxycinnamic acids. Pearson’s correlation coefficient analysis strongly supported the PLS analysis findings.

## Conclusion

This study provides the first evidence linking specific metabolites to some of the most important pharmacological effects of two Lamiaceae species, *Salvia rosmarinus* and *Origanum vulgare*. While *S. rosmarinus* Spenn. exhibited superior anti-inflammatory and radical scavenging activities, *O. vulgare* L. showed a stronger reducing power. PLS and Pearson’s correlation coefficients confirmed the strong positive correlation between different hydroxycinnamic acids derivatives (i.e., Yunnaneic acid F, salvianolic acid K, and rosmarinic acid), diterpenes (i.e., oridonin, carnosic acid, hydroxyrosmanol, rosmic acid, dimethoxyrosmanol, rosmadial, and tetrahydro-7-hydroxyrosmariquinone), triterpenes (i.e., asiatic acid, corosolic acid, and 3-oxours-12-en-20,28-olide), and flavonoid derivatives (i.e., nepitrin, isorhamnetin, and hesperetin) mainly present in *S. rosmarinus* Spenn. extract with strong antibacterial and DPPH radical scavenging activities. Moreover, the anti-inflammatory activity was positively correlated with flavonoid derivatives (i.e., luteolin-acetyl-*O*-hexuronide, apigenin, dimethylquercetin, and cirsimaritin), diterpenes (i.e., rosmanol, hydroxy-*O*-methylrosmanol, hydroxyrosmadial, and carnosol), and hydroxycinnamic acids (i.e., salvianolic acid B and lithospermic acids). The antioxidant activity evaluated as FRAP reducing power was positively correlated with benzyl derivatives (i.e., hydroxyphenol hexoside, hydroxyphenol hexoside derivative, and origanine B or C), organic acids and esters (i.e., oxoadipic acid, corchorifatty acid F, malic acid, and citric acid), and hydroxycinnamic acids (i.e., lithospermic acid derivative and cleroden J). This study can be applied to a wide range of medicinal plants, driving a deeper exploration of the correlation between bioactivities and metabolite composition. In turn, this paves the way for a profound understanding of mechanisms of action, pharmacokinetics, and structure-activity relationships, and advances the development of herbal medicine practice. Bioinformatic tools also enable this approach to be expanded toward other therapeutic targets.

## Supplementary Information

Below is the link to the electronic supplementary material.

Supplementary Material 1

## Data Availability

All data generated or analyzed during this study are included in this published article and its supplementary information file.

## References

[1] Saral, Ö., Baltaş, N. & Karaköse, M. An Inhibition potential on some metabolic enzymes (urease and Xanthine oxidase), essential oil contents and antioxidant effect of *Sideritis Lanata* L. *Chem. Pap*. **78**, 8211–8217 (2024).

[2] Çelik, G. et al. Biological activity, and volatile and phenolic compounds from five lamiaceae species. *Flavour. Fragr. J.* **36**, 223–232 (2021).

[3] Şen, G., Akbulut, S. & Karaköse, M. Ethnopharmacological study of medicinal plants in Kastamonu Province (Türkiye). *Open. Chem.* **20**, 873–911 (2022).

[4] Karaköse, M., Akbulut, S. & Özkan, Z. C. Ethnobotanical study of medicinal plants in Torul district, Turkey. *Bangladesh J. Plant. Taxon*. **26**, 29–37 (2019).

[5] Ramos da Silva, L. R. et al. Lamiaceae essential oils, phytochemical profile, antioxidant, and biological activities. *Evidence-Based Complement. Altern. Med.* **2021**, 1–18 (2021). 10.1155/2021/6748052 PMC8692021 34950215

[6] Mahendran, G., Rahman, L. & Ethnomedicinal Phytochemical and Pharmacological updates on peppermint (*Mentha × Piperita* L.)—A review. *Phyther Res.* **34**, 2088–2139 (2020). 10.1002/ptr.6664 32173933

[7] Taamalli, A. et al. LC-MS‐based metabolite profiling of methanolic extracts from the medicinal and aromatic species *Mentha pulegium* and *Origanum Majorana*. *Phytochem Anal.* **26**, 320–330 (2015). 25982347 10.1002/pca.2566

[8] Tzima, K., Brunton, N. & Rai, D. Qualitative and quantitative analysis of polyphenols in lamiaceae plants—A review. *Plants* **7**, 25 (2018). 29587434 10.3390/plants7020025 PMC6027318

[9] Bekut, M. et al. Potential of selected lamiaceae plants in anti(retro)viral therapy. *Pharmacol. Res.* **133**, 301–314 (2018). 29258916 10.1016/j.phrs.2017.12.016 PMC7129285

[10] Gheisary, B., Ashrafi-Saeidlou, S., Hassani, A. & Fattahi, M. Enhancing antioxidant and antibacterial activities of *Cuminum cyminum*, *Origanum vulgare*, and *Salvia officinalis* essential oils through a synergistic perspective. *Sci. Rep.* **15**, 26728 (2025). 40702010 10.1038/s41598-025-10814-4 PMC12287461

[11] Uba, A. I. et al. Antioxidant and enzyme inhibitory properties, and HPLC–MS/MS profiles of different extracts of *Arabis carduchorum* Boiss.: an endemic plant to Turkey. *Appl. Sci.* **12**, 6561 (2022).

[12] Kurt-Celep, İ. et al. Unraveling the chemical profile, antioxidant, enzyme inhibitory, cytotoxic potential of different extracts from *Astragalus Caraganae*. *Arch. Pharm. (Weinheim)*. **356**, 2300263 (2023). 10.1002/ardp.202300263 37434089

[13] Pezzani, R., Vitalini, S. & Iriti, M. Bioactivities of *Origanum vulgare* L.: an update. *Phytochem Rev.* **16**, 1253–1268 (2017).

[14] Stelter, K. et al. Effects of oregano on performance and immunmodulating factors in weaned piglets. *Arch. Anim. Nutr.* **67**, 461–476 (2013). 24228909 10.1080/1745039X.2013.858897

[15] Zakarya, A. Y., Rasheed, D. M. & Farag, M. A. Valorization of aromatic plant distillation by-products (solid biomass, wastewater, and aromatic water): case study on the lamiaceae family. *Waste Biomass Valoriz.* **1–23**10.1007/s12649-025-03173-8 (2025).

[16] Ramos-González, E. J., Bitzer-Quintero, O. K., Ortiz, G. & Hernández-Cruz, J. J. Ramírez-Jirano, L. J. Relationship between inflammation and oxidative stress and its effect on multiple sclerosis. *Neurología* **39**, 292–301 (2024). 38553104 10.1016/j.nrleng.2021.10.010

[17] Baky, M. H., Maamoun, A. A., Nicolescu, A., Mocan, A. & Farag, M. A. Multi-targeted MS-based metabolomics fingerprinting of black and white pepper coupled with molecular networking in relation to their *in vitro* antioxidant and antidiabetic effects. *RSC Adv.* **15**, 27606–27622 (2025). 40761908 10.1039/d5ra03714j PMC12320482

[18] CLSI. *Performance Standards for Antimicrobial Susceptibility TestingNo Title* (CLSI, 2020).

[19] Badawy, A. E., Rasheed, D. M., Gebaly, E., El-Shiekh, R. & El-Gayed, S. E. G. Comparative analysis of the antimicrobial and antibiofilm activities of *Terminalia Catalpa* Linn. fruit polyphenolic-enriched and lipoidal-enriched extracts. *Bull. Pharm. Sci. Assiut Univ.*. 10.21608/bfsa.2025.379398.2532 (2025).

[20] Saleh, M. M., Abbas, H. A. & Askoura, M. M. Repositioning secnidazole as a novel virulence factors attenuating agent in *Pseudomonas aeruginosa*. *Microb. Pathog*. **127**, 31–38 (2019). 30500409 10.1016/j.micpath.2018.11.042

[21] Kot, B., Sytykiewicz, H. & Sprawka, I. Expression of the biofilm-associated genes in methicillin-resistant *Staphylococcus aureus* in biofilm and planktonic conditions. *Int. J. Mol. Sci.* **19**, 3487 (2018). 30404183 10.3390/ijms19113487 PMC6274806

[22] Livak, K. J. & Schmittgen, T. D. Analysis of relative gene expression data using real-time quantitative PCR and the 2 – ∆∆CT method. *Methods* **25**, 402–408 (2001). 11846609 10.1006/meth.2001.1262

[23] Karaçelik, A. A., Türkuçar, S. A. & Karaköse, M. Phytochemical composition and biological activities of *Angelica sylvestris* L. var. *Stenoptera* Avé-Lall ex Boiss.: an endangered medicinal plant of Northeast Turkey. *Chem. Biodivers.* **19**, e202200552 (2022). 36085404 10.1002/cbdv.202200552

[24] Benzie, I. F. F. & Strain, J. J. The ferric reducing ability of plasma (FRAP) as a measure of antioxidant power: the FRAP assay. *Anal. Biochem.* **239**, 70–76 (1996). 8660627 10.1006/abio.1996.0292

[25] El-Sayed, H. M. et al. Metabolomics analysis of *Cucumis Melo* var. *Flexuosus* organs in correlation to its anti-inflammatory activity aided by chemometrics. *J. Pharm. Biomed. Anal.* **252**, 116512 (2025). 39405783 10.1016/j.jpba.2024.116512

[26] Younis, I. Y. et al. Exploring geographic variations in Quinoa grains: unveiling anti-Alzheimer activity via GC–MS, LC-QTOF-MS/MS, molecular networking, and chemometric analysis. *Food Chem.* **465**, 141918 (2025). 39541691 10.1016/j.foodchem.2024.141918

[27] Mukaka, M. M. A guide to appropriate use of correlation coefficient in medical research. *Malawi Med. J.* **24**, 69–71 (2012). 23638278 PMC3576830

[28] Jiang, R. W. et al. Chemistry and biological activities of caffeic acid derivatives from *Salvia miltiorrhiza*. *Curr. Med. Chem.* **12**, 237–246 (2005). 15638738 10.2174/0929867053363397

[29] Ruiz, A. et al. Isolation and structural Elucidation of Anthocyanidin 3,7-β-*O*-diglucosides and caffeoyl-glucaric acids from Calafate berries. *J. Agric. Food Chem.* **62**, 6918–6925 (2014). 24697704 10.1021/jf5012825

[30] Li, Z. et al. Nuciferine and Paeoniflorin can be quality markers of Tangzhiqing tablet, a Chinese traditional patent medicine, based on the qualitative, quantitative and dose-exposure-response analysis. *Phytomedicine* **44**, 155–163 (2018). 29519686 10.1016/j.phymed.2018.02.006

[31] Hossain, M. B., Rai, D. K., Brunton, N. P., Martin-Diana, A. B. & Barry-Ryan, C. Characterization of phenolic composition in lamiaceae spices by LC-ESI-MS/MS. *J. Agric. Food Chem.* **58**, 10576–10581 (2010). 20825192 10.1021/jf102042g

[32] Li, Q. et al. Clerodens E–J, antibacterial caffeic acid derivatives from the aerial part of *Clerodendranthus spicatus*. *Fitoterapia* **114**, 110–114 (2016). 27593446 10.1016/j.fitote.2016.08.021

[33] El-Gazar, A. A., Emad, A. M., Ragab, G. M. & Rasheed, D. M. *Mentha pulegium* L. (Pennyroyal, Lamiaceae) extracts impose abortion or fetal-mediated toxicity in pregnant rats; evidenced by the modulation of pregnancy hormones, MiR-520, MiR-146a, TIMP-1 and MMP-9 protein expressions, inflammatory state, certain. *Toxins (Basel).* **14**, 347 (2022). 10.3390/toxins14050347 PMC9147109 35622593

[34] Nuengchamnong, N., Krittasilp, K. & Ingkaninan, K. Characterisation of phenolic antioxidants in aqueous extract of *Orthosiphon grandiflorus* tea by LC–ESI-MS/MS coupled to DPPH assay. *Food Chem.* **127**, 1287–1293 (2011). 25214128 10.1016/j.foodchem.2011.01.085

[35] Chen, H., Zhang, Q., Wang, X., Yang, J. & Wang, Q. Qualitative analysis and simultaneous quantification of phenolic compounds in the aerial parts of *Salvia miltiorrhiza* by HPLC-DAD and ESI/MS n. *Phytochem Anal.* **22**, 247–257 (2011). 21046689 10.1002/pca.1272

[36] Dueñas, M., Sánchez-Acevedo, T., Alcalde-Eon, C. & Escribano-Bailón, M. T. Effects of different industrial processes on the phenolic composition of white and brown Teff (*Eragrostis tef* (Zucc.) Trotter). *Food Chem.* **335**, 127331 (2021). 32739802 10.1016/j.foodchem.2020.127331

[37] Ćirić, A., Prosen, H., Jelikić-Stankov, M. & Đurđević, P. Evaluation of matrix effect in determination of some bioflavonoids in food samples by LC–MS/MS method. *Talanta* **99**, 780–790 (2012). 22967624 10.1016/j.talanta.2012.07.025

[38] Justesen, U. Negative atmospheric pressure chemical ionisation low-energy collision activation mass spectrometry for the characterisation of flavonoids in extracts of fresh herbs. *J. Chromatogr. A*. **902**, 369–379 (2000). 11192169 10.1016/s0021-9673(00)00861-x

[39] Fabre, N., Rustan, I., de Hoffmann, E. & Quetin-Leclercq, J. Determination of flavone, flavonol, and Flavanone aglycones by negative ion liquid chromatography electrospray ion trap mass spectrometry. *J. Am. Soc. Mass. Spectrom.* **12**, 707–715 (2001). 11401161 10.1016/S1044-0305(01)00226-4

[40] McNab, H., Ferreira, E. S. B., Hulme, A. N. & Quye, A. Negative ion ESI–MS analysis of natural yellow dye flavonoids—An isotopic labelling study. *Int. J. Mass. Spectrom.* **284**, 57–65 (2009).

[41] Mi, H. et al. Identification of *Daphne Genkwa* and its vinegar-processed products by ultraperformance liquid chromatography–quadrupole time-of-flight mass spectrometry and chemometrics. *Molecules* **28**, 3990 (2023). 37241730 10.3390/molecules28103990 PMC10223954

[42] Grayer, R. J., Veitch, N. C., Kite, G. C., Price, A. M. & Kokubun, T. Distribution of 8-oxygenated leaf-surface flavones in the genus *Ocimum*. *Phytochemistry* **56**, 559–567 (2001). 11281133 10.1016/s0031-9422(00)00439-8

[43] Pandey, R. & Kumar, B. HPLC–QTOF–MS/MS-based rapid screening of phenolics and triterpenic acids in leaf extracts of *Ocimum* species and their interspecies variation. *J. Liq Chromatogr. Relat. Technol.* **39**, 225–238 (2016).

[44] Birtić, S., Dussort, P., Pierre, F. X., Bily, A. C. & Roller, M. Carnosic acid. *Phytochemistry* **115**, 9–19 (2015). 25639596 10.1016/j.phytochem.2014.12.026

[45] Uritu, C. M. et al. Medicinal plants of the family Lamiaceae in pain therapy: A review. *Pain Res. Manag.* **2018**, 1–44 (2018). 10.1155/2018/7801543 PMC5964621 29854039

[46] Takenaka, M. et al. New antimicrobial substances against *Streptomyces scabies* from Rosemary (*Rosmarinus officinalis* L). *Biosci. Biotechnol. Biochem.* **61**, 1440–1444 (1997).

[47] Zhang, Y. et al. Degradation study of carnosic acid, carnosol, Rosmarinic acid, and Rosemary extract (*Rosmarinus officinalis* L.) assessed using HPLC. *J. Agric. Food Chem.* **60**, 9305–9314 (2012). 22881034 10.1021/jf302179c

[48] Koutsoulas, A., Čarnecká, M., Slanina, J., Tóth, J. & Slaninová, I. Characterization of phenolic compounds and antiproliferative effects of *Salvia pomifera* and *Salvia fruticosa* extracts. *Molecules* **24**, 2921 (2019). 31408993 10.3390/molecules24162921 PMC6720736

[49] Stohs, S. J., Miller, H. & Kaats, G. R. A review of the efficacy and safety of Banaba (*Lagerstroemia speciosa* L.) and corosolic acid. *Phyther Res.* **26**, 317–324 (2012). 10.1002/ptr.3664 22095937

[50] Olawuwo, O. S., Famuyide, I. M. & McGaw, L. J. Antibacterial and antibiofilm activity of selected medicinal plant leaf extracts against pathogens implicated in poultry diseases. *Front. Vet. Sci.* **9**, 820304 (2022). 35310417 10.3389/fvets.2022.820304 PMC8926311

[51] Vestergaard, M., Frees, D. & Ingmer, H. Antibiotic resistance and the MRSA problem. *Microbiol. Spectr.* **7**, 10–1128 (2019). 10.1128/microbiolspec.gpp3-0057-2018 PMC11590431 30900543

[52] Patel, H. & Rawat, S. A genetic regulatory see-saw of biofilm and virulence in MRSA pathogenesis. *Front. Microbiol.* **14**, 1204428 (2023). 37434702 10.3389/fmicb.2023.1204428 PMC10332168

[53] Chen, L. et al. Inflammatory responses and inflammation-associated diseases in organs. *Oncotarget* **9**, 7204–7218 (2018). 29467962 10.18632/oncotarget.23208 PMC5805548

[54] Liu, J., Du, C., Beaman, H. T. & Monroe, M. B. B. Characterization of phenolic acid antimicrobial and antioxidant structure–property relationships. *Pharmaceutics* **12**, 419 (2020). 32370227 10.3390/pharmaceutics12050419 PMC7285200

[55] Mróz, M. & Kusznierewicz, B. Phytochemical screening and biological evaluation of Greek Sage (*Salvia fruticosa* Mill.) extracts. *Sci. Rep.* **13**, 22309 (2023). 38102229 10.1038/s41598-023-49695-w PMC10724190

[56] Habtemariam, S. Anti-inflammatory therapeutic mechanisms of natural products: insight from Rosemary diterpenes, carnosic acid and carnosol. *Biomedicines* **11**, 545 (2023). 36831081 10.3390/biomedicines11020545 PMC9953345

[57] Funakoshi-Tago, M., Nakamura, K., Tago, K., Mashino, T. & Kasahara, T. Anti-inflammatory activity of structurally related flavonoids, Apigenin, Luteolin and Fisetin. *Int. Immunopharmacol.* **11**, 1150–1159 (2011). 21443976 10.1016/j.intimp.2011.03.012

[58] Dessai, A., Shetty, N., Saralaya, V., Natarajan, S. & Mala, K. Carnosic acid as an intracanal medicament performs better than triple antibiotic paste and calcium hydroxide to eradicate *Enterococcus faecalis* from root canal: an *in vitro* confocal laser scanning microscopic study. *J. Conserv. Dent.* **25**, 20 (2022). 35722077 10.4103/jcd.jcd_317_21 PMC9200188

[59] Slobodníková, L., Fialová, S., Hupková, H. & Grančai, D. Rosmarinic acid interaction with planktonic and biofilm *Staphylococcus aureus*. *Nat. Prod. Commun.* **8**, 1934578X1300801223 (2013). 24555289

[60] Zhao, Y. et al. Lithospermic acid alleviates oxidative stress and inflammation in DSS-induced colitis through Nrf2. *Eur. J. Pharmacol.* **995**, 177390 (2025). 39956261 10.1016/j.ejphar.2025.177390

