# Supplementary material for: Comparative metabolomic profiling and chemometric correlation of Salvia rosmarinus Spenn. and Origanum vulgare L. with antibacterial, antioxidant and anti-inflammatory activities
Source: Sci Rep. 2025 Dec 3;16:990. doi: 10.1038/s41598-025-30607-z (PMC12783746; doi:10.1038/s41598-025-30607-z)
Supplement: Supplementary file 1 — Supplementary Material 1 [file 41598_2025_30607_MOESM1_ESM.docx]

**Supplementary file**

**Comparative Metabolomic Profiling and Chemometric Correlation of *Salvia rosmarinus* Spenn. and *Origanum vulgare* L. with Antibacterial, Antioxidant and Anti-Inflammatory Activities**


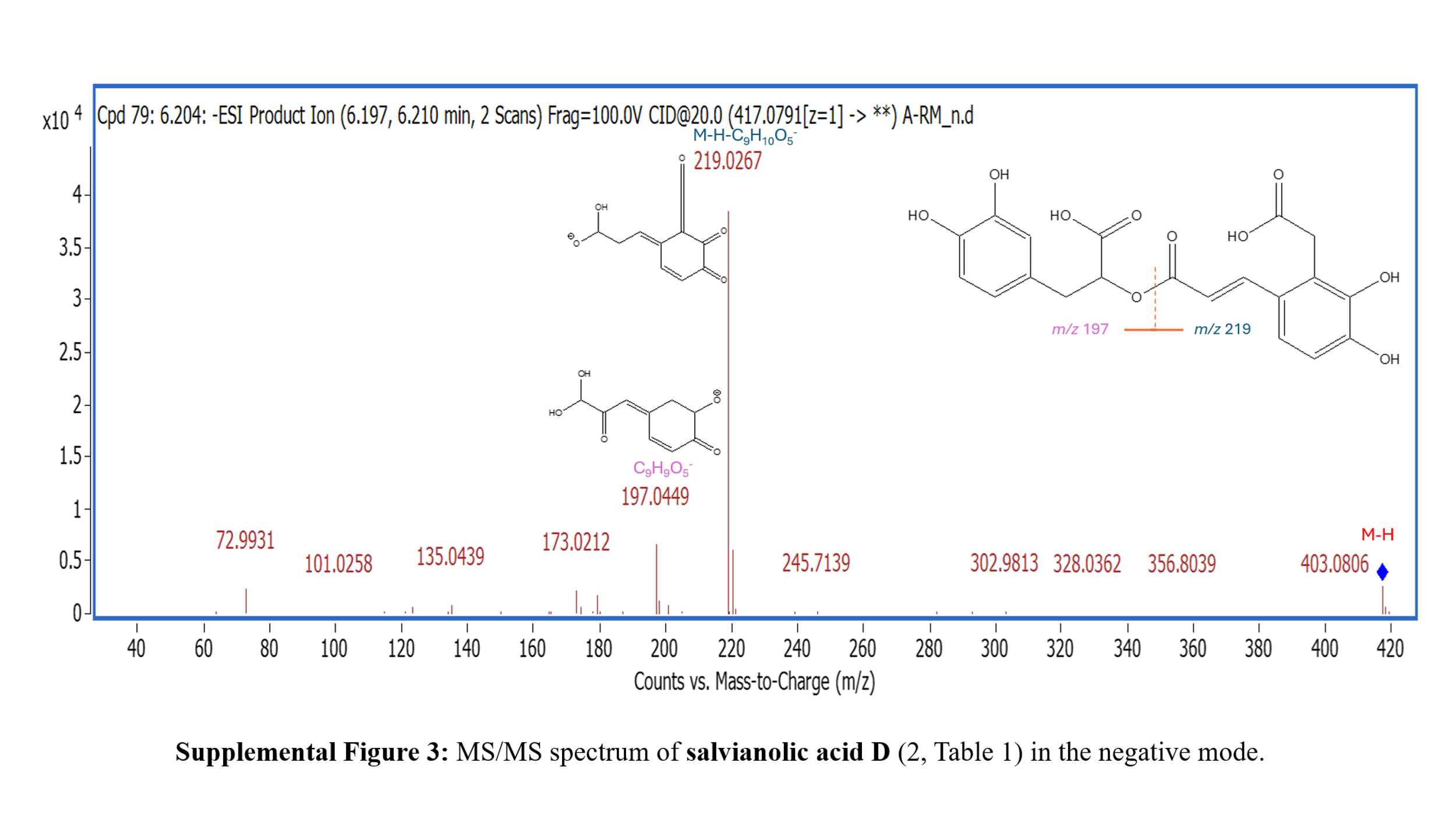


**Figure 1S:** MS/MS spectrum of salvianolic acid D (7, **Table 1**) in the negative mode.


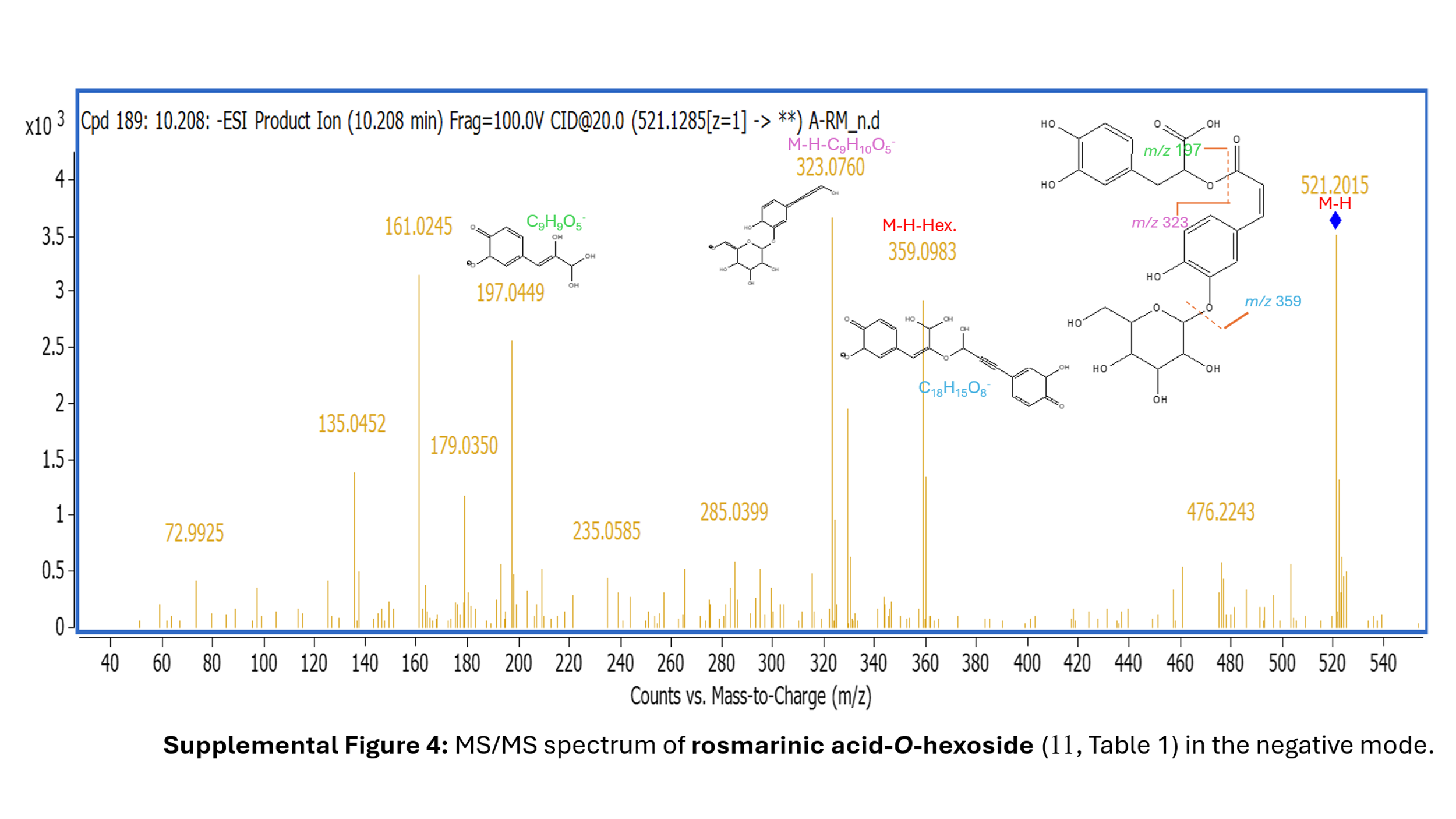


**Figure 2S:** MS/MS spectrum of rosmarinic acid-O-hexoside (16, **Table** **1**) in the negative mode.


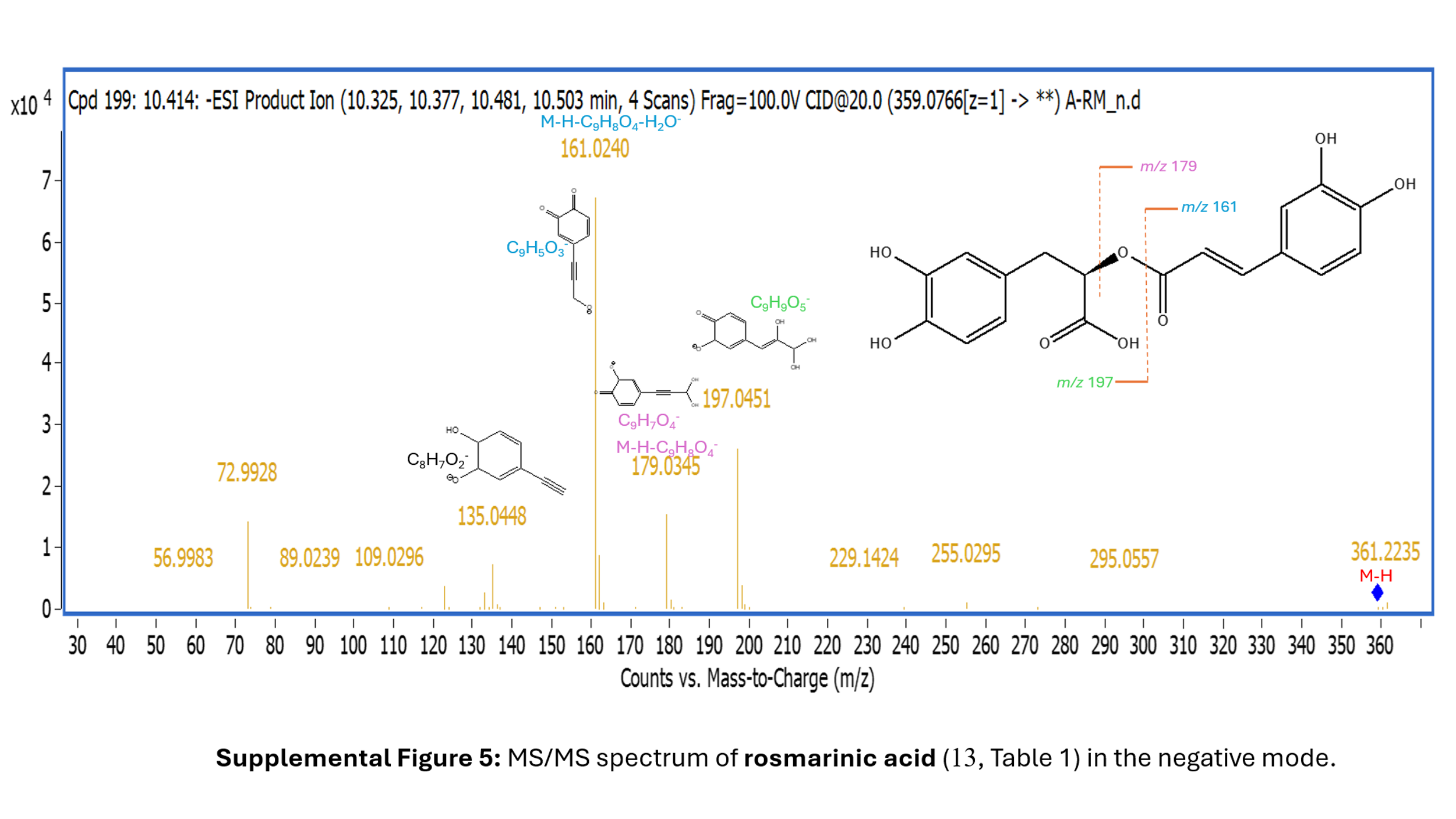


**Figure 3S:** MS/MS spectrum of rosmarinic acid (18, **Table 1**) in the negative mode.


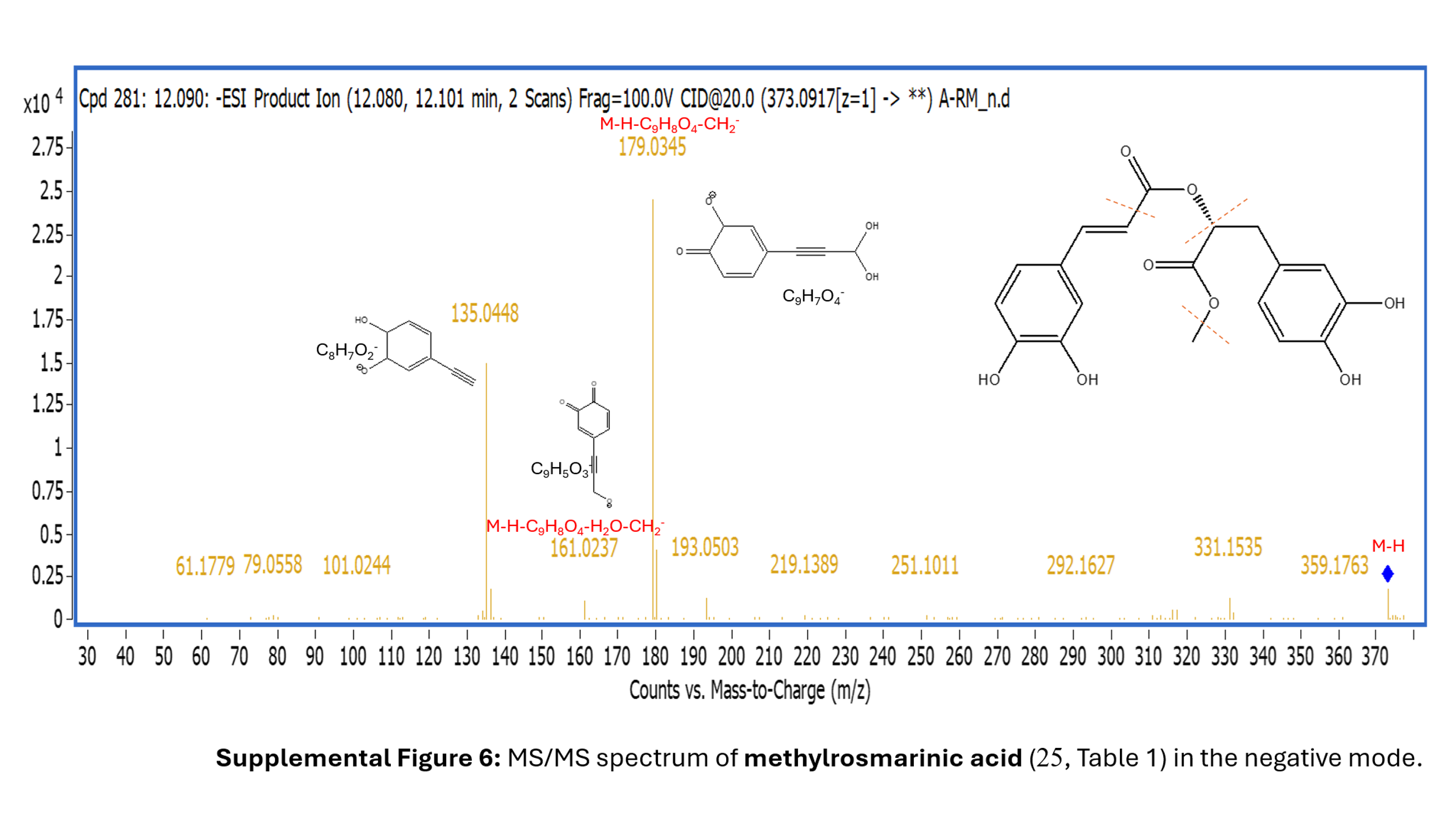


**Figure 4S:** MS/MS spectrum of methylrosmarinic acid (30, **Table 1**) in the negative mode.


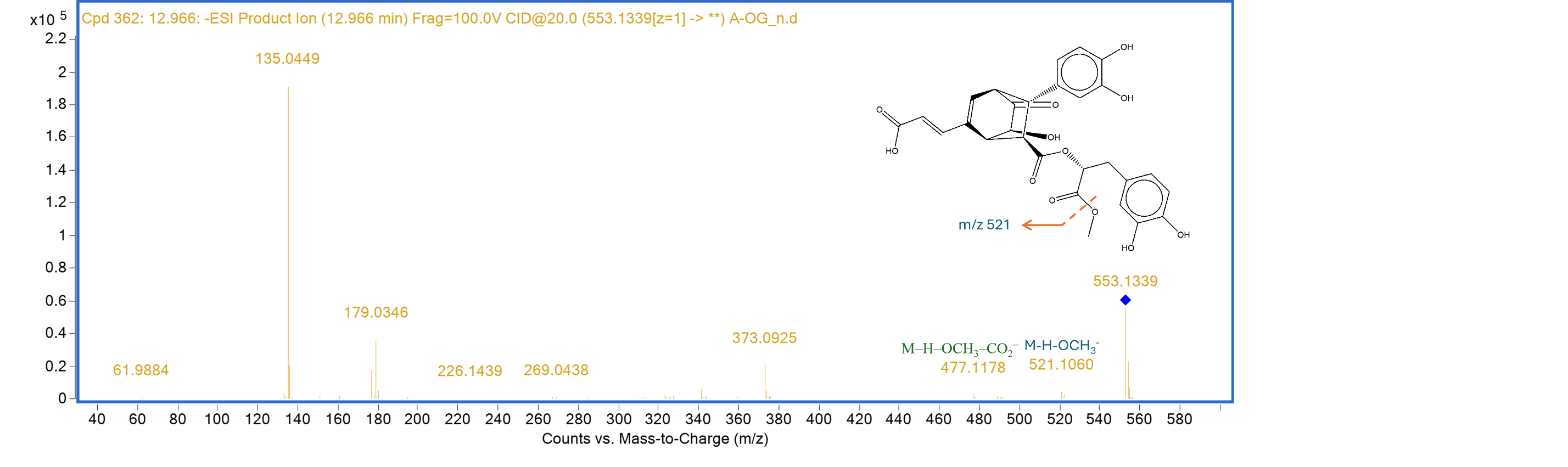


**Figure 5S:** MS/MS spectrum of Cleroden J (33, **Table 1**) in the negative mode.


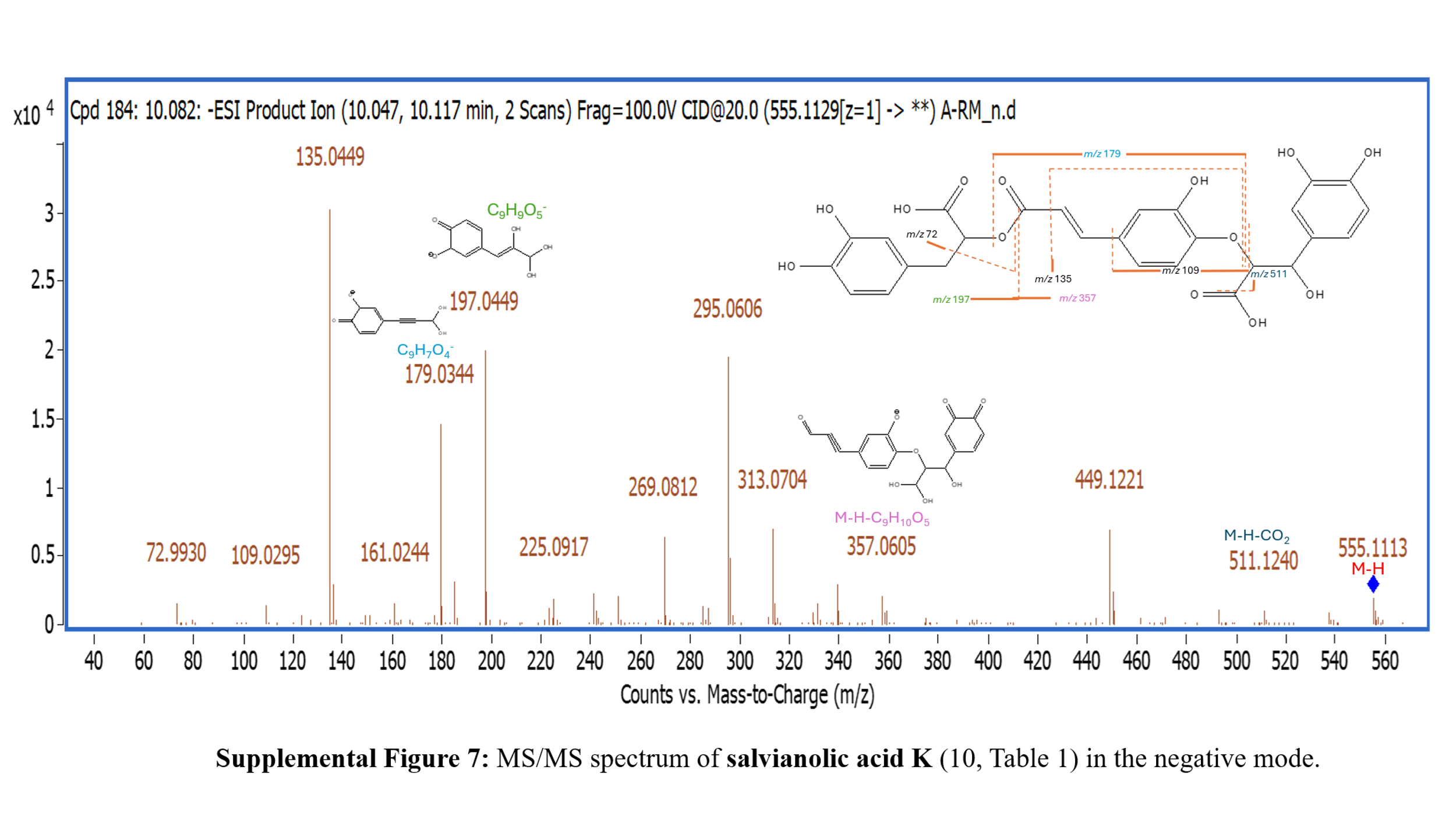


**Figure 6S**: MS/MS spectrum of salvianolic acid K (15, **Table 1**) in the negative mode.


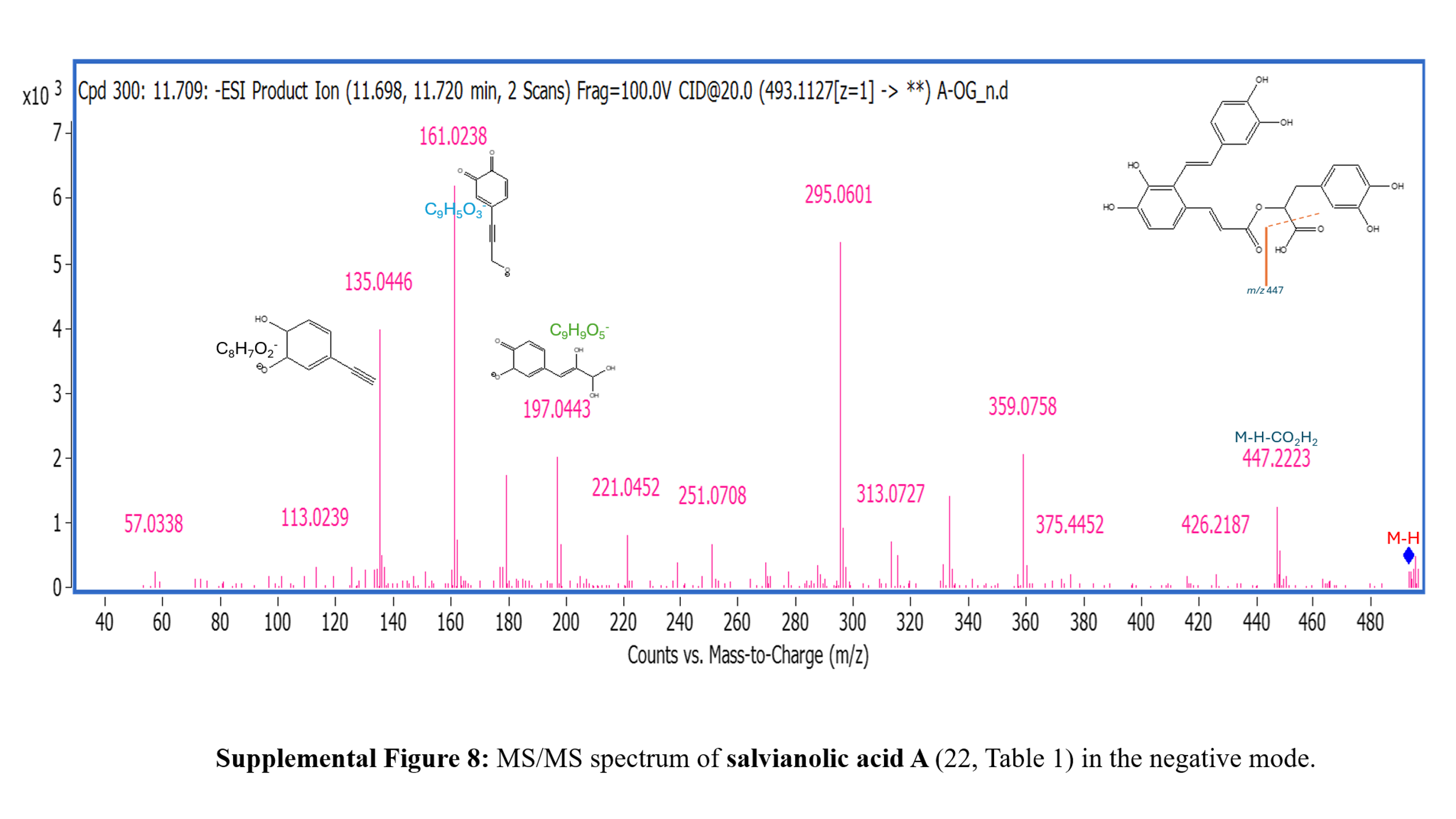


**Figure 7S:** MS/MS spectrum of salvianolic acid A (27, **Table** **1**) in the negative mode.


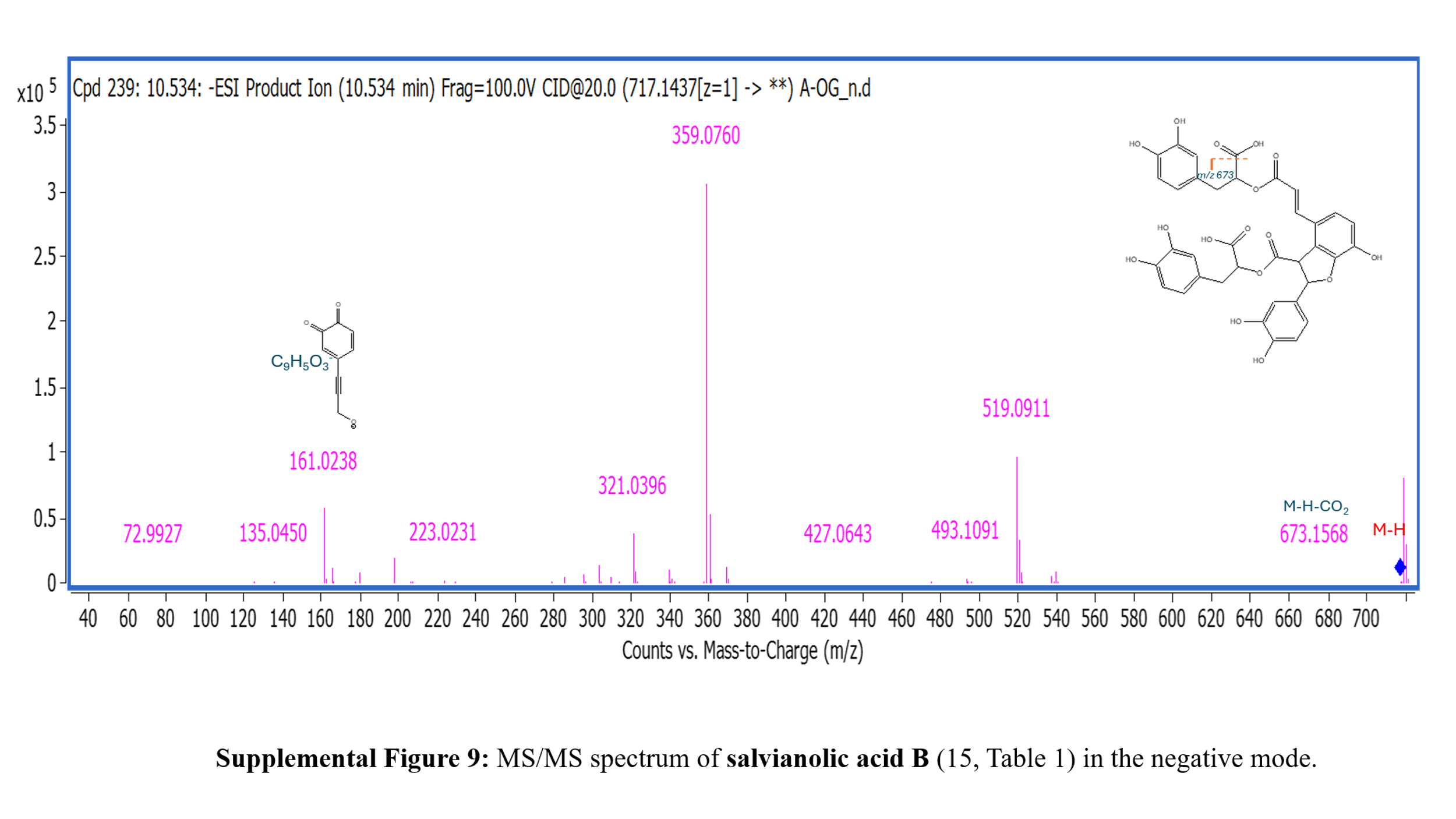


**Figure 8S:** MS/MS spectrum of salvianolic acid B (20, **Table 1**) in the negative mode.


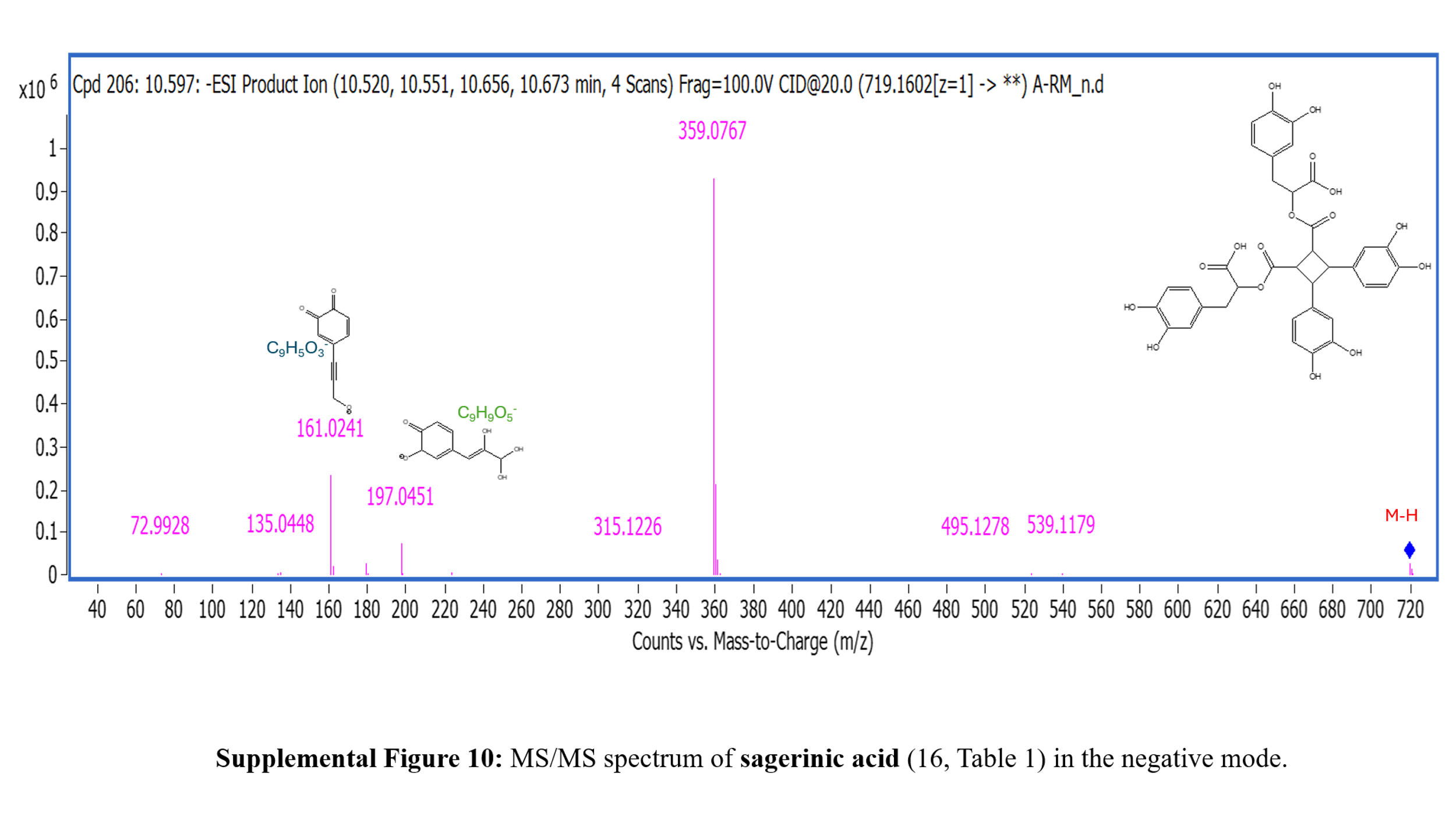


**Figure 9S:** MS/MS spectrum of sagerinic acid (21, **Table 1**) in the negative mode.


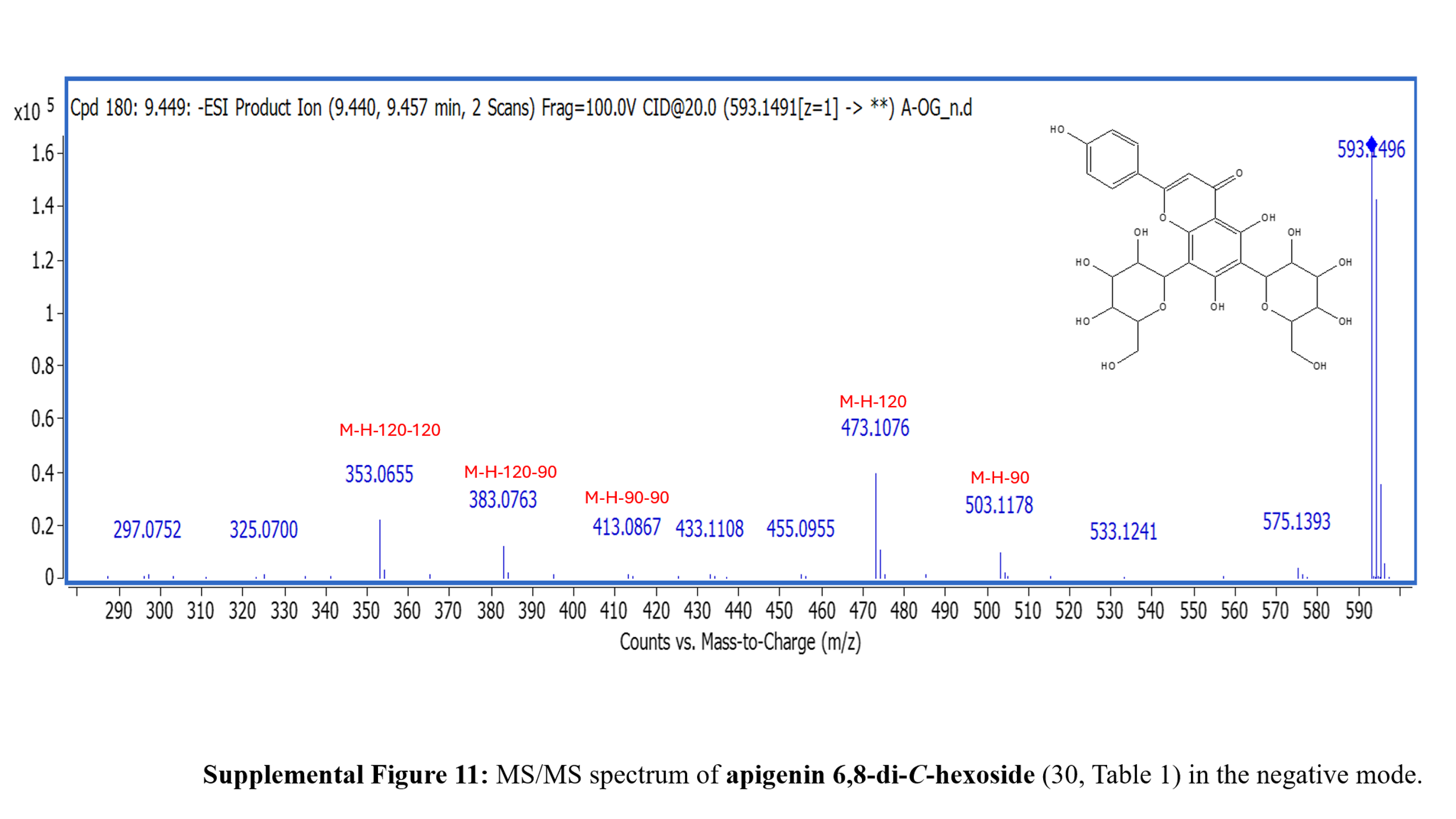


**Figure 10S:** MS/MS spectrum of apigenin 6,8-di-C-hexoside (52, **Table 1**) in the negative mode.


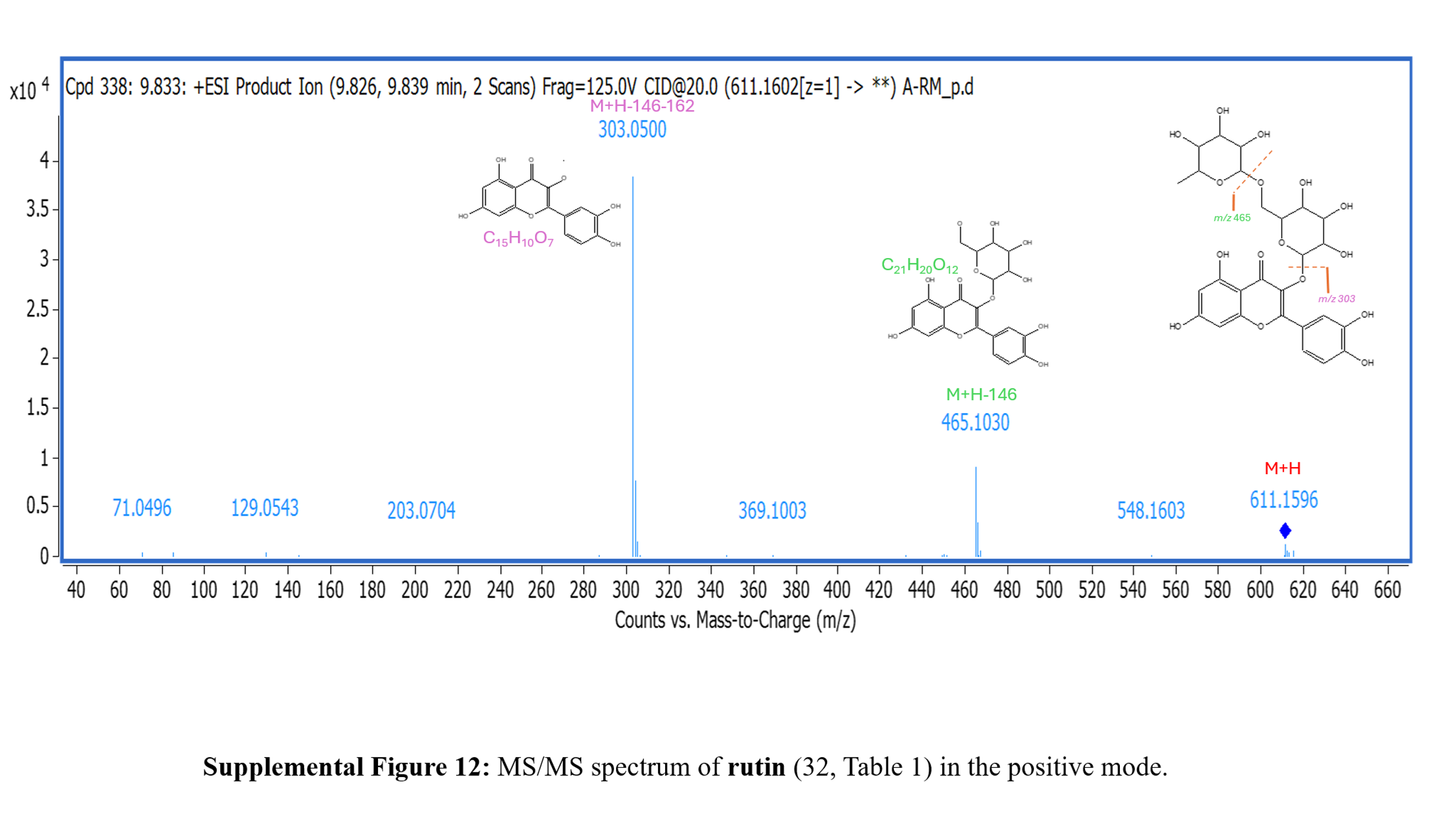


**Figure 11S:** MS/MS spectrum of rutin (92, **Table 1**) in the positive mode.


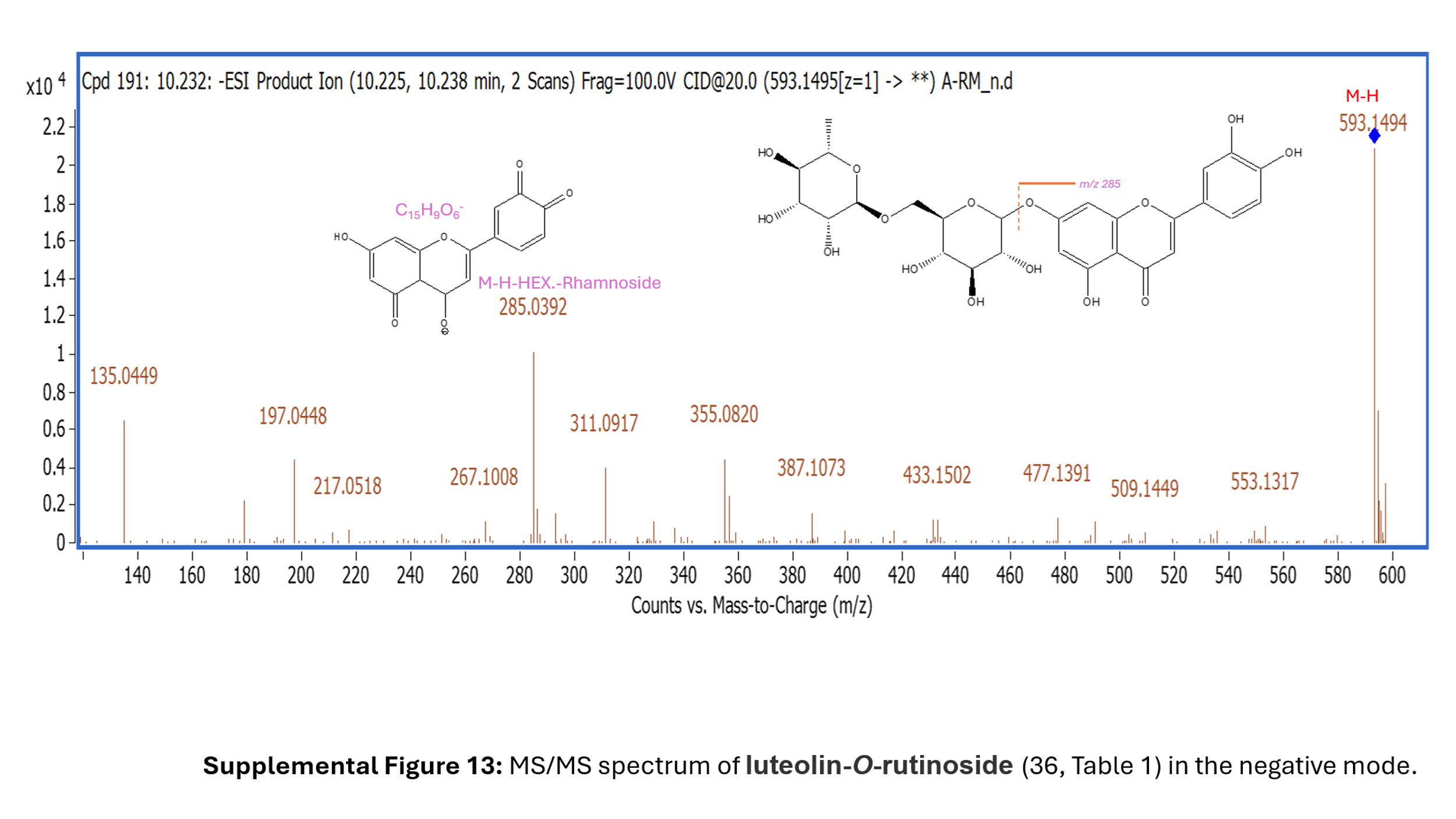


**Figure 12S:** MS/MS spectrum of luteolin-O-rutinoside (56, **Table 1**) in the negative mode.


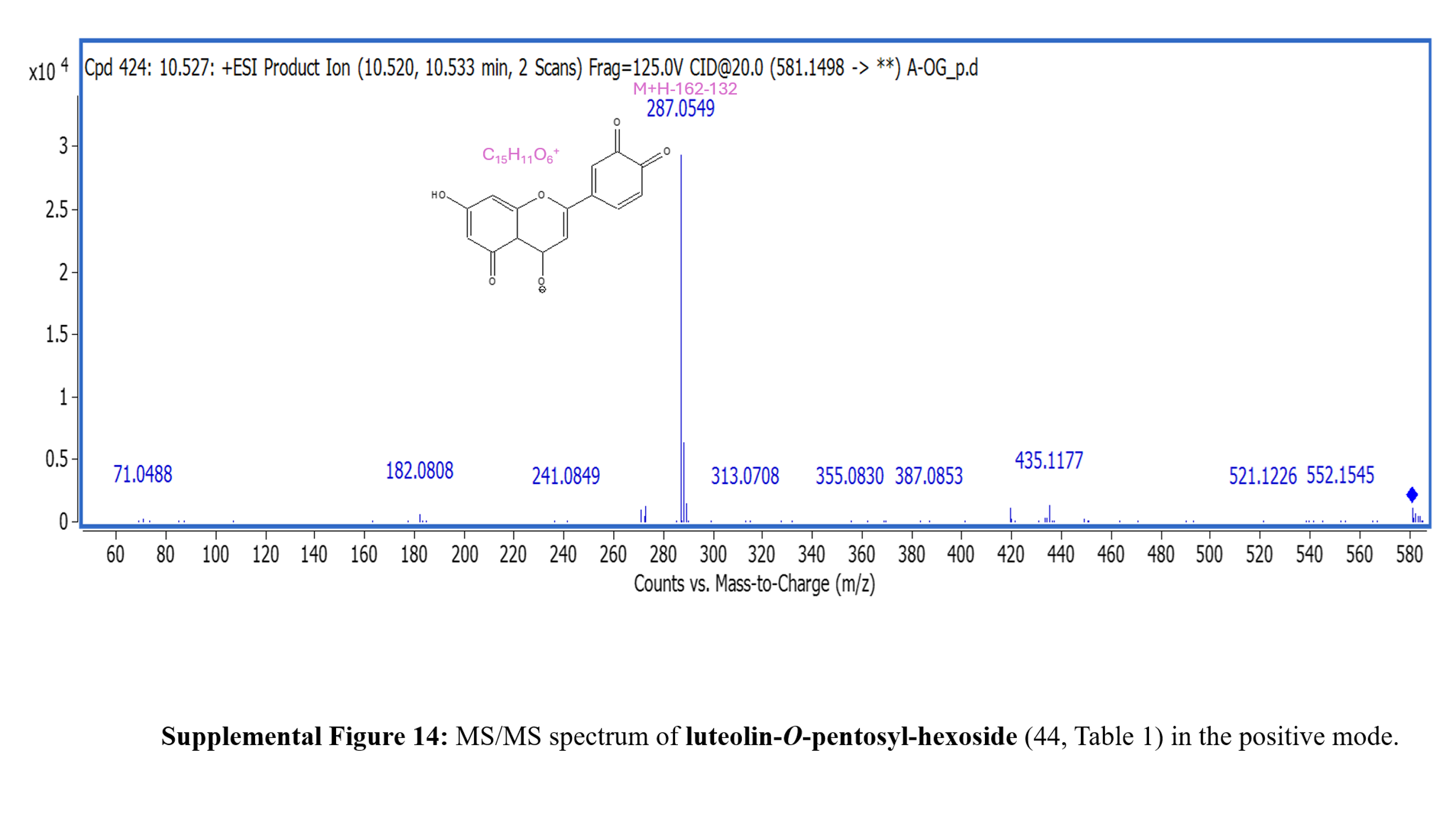


**Figure 13S:** MS/MS spectrum of luteolin-O-pentosyl-hexoside (61, **Table 1**) in the positive mode.


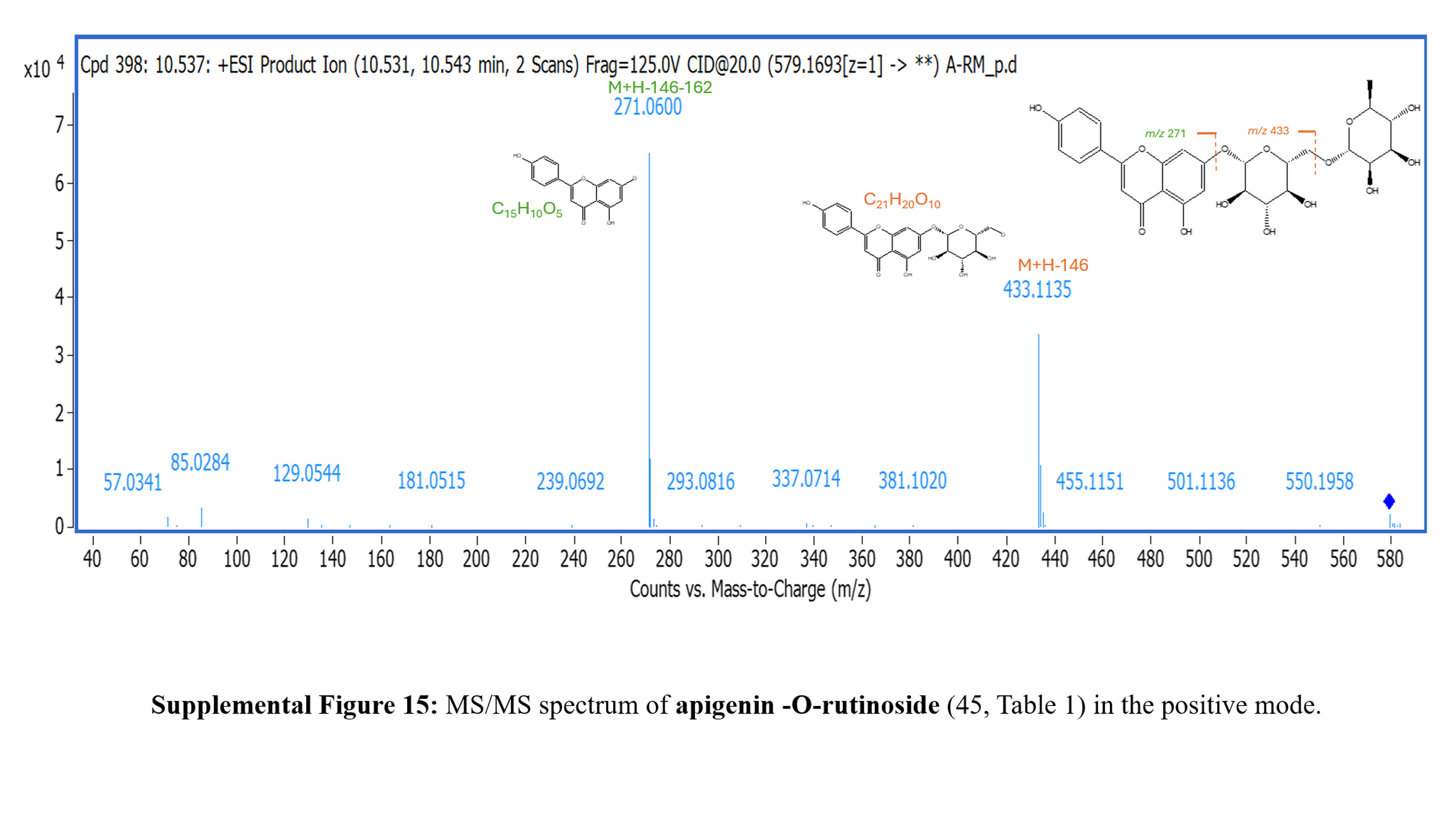


**Figure 14S:** MS/MS spectrum of apigenin-O-rutinoside (62, **Table** **1**) in the positive mode.


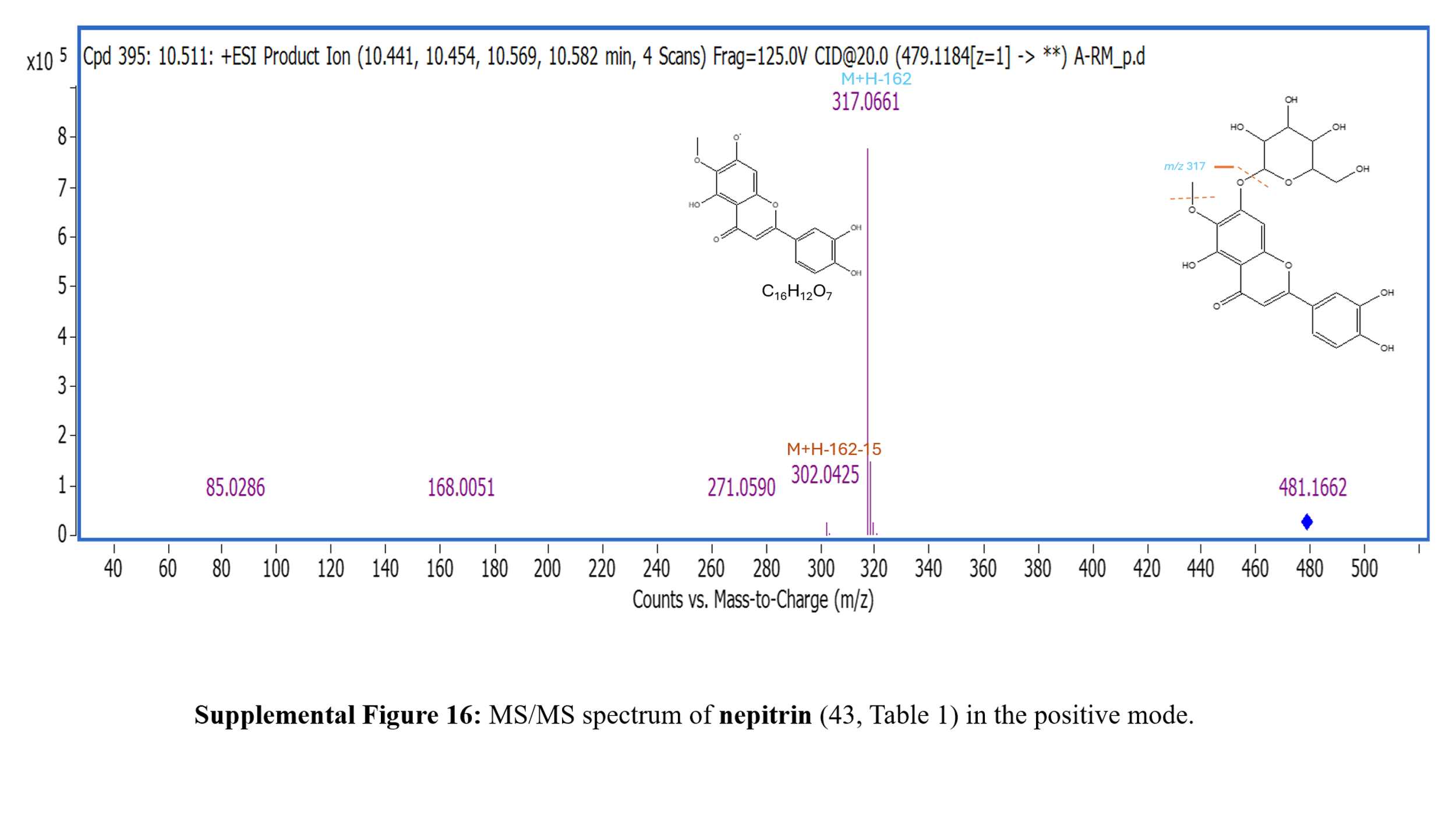


**Figure 15S:** MS/MS spectrum of nepitrin (60, **Table 1**) in the positive mode.


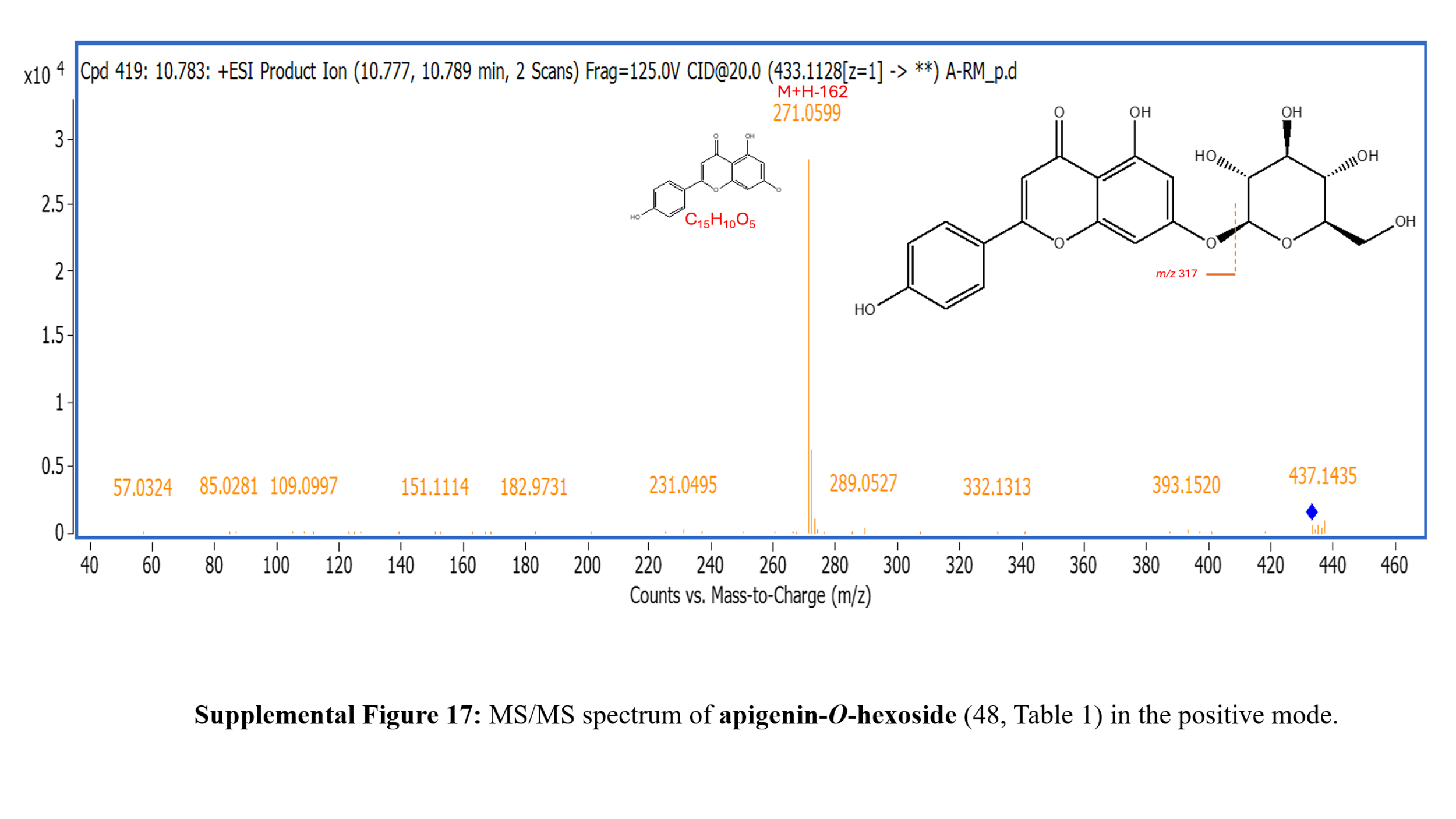


**Figure 16S:** MS/MS spectrum of apigenin-O-hexoside (65, **Table 1**) in the positive mode.


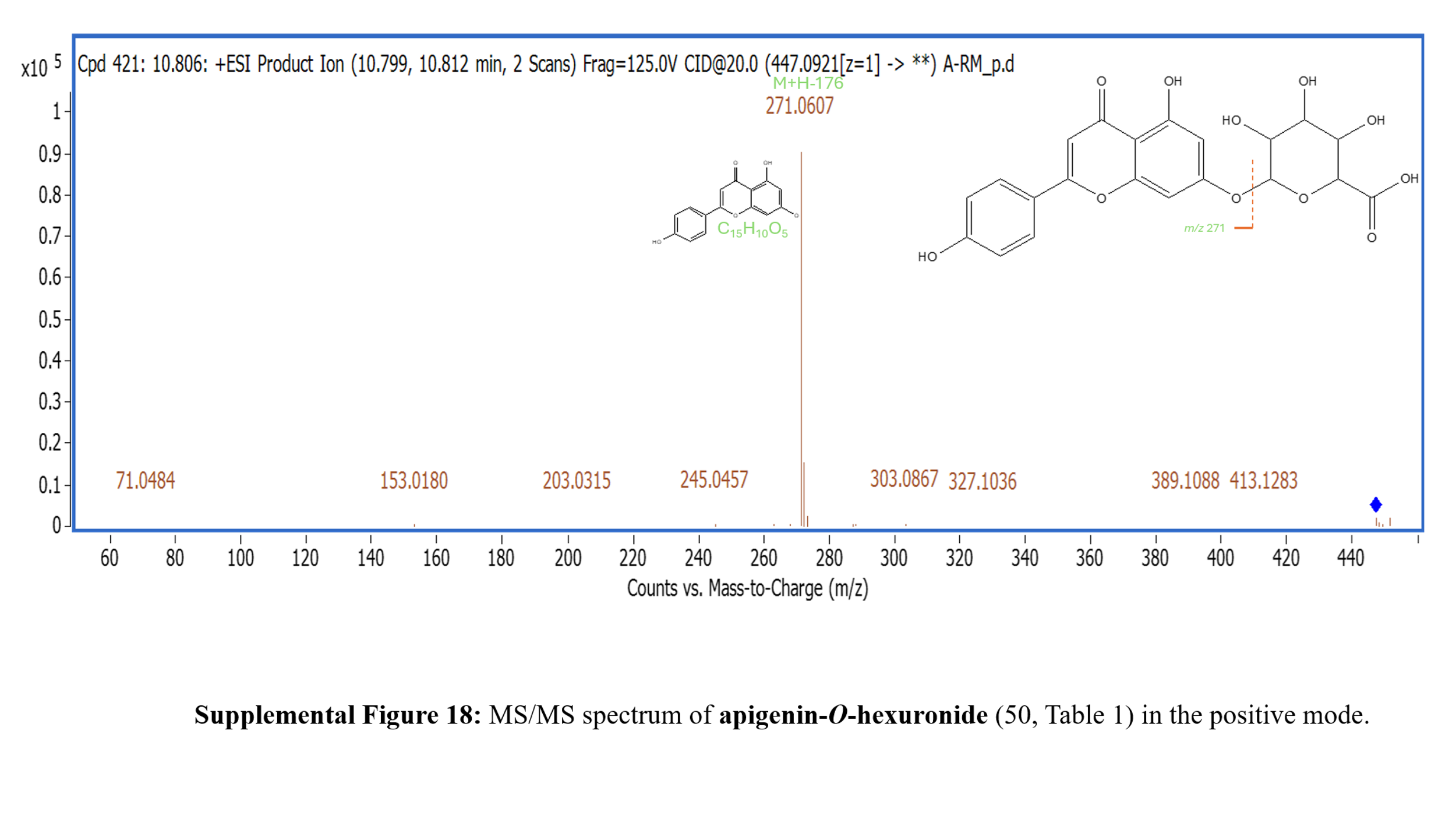


**Figure 17S:** MS/MS spectrum of apigenin-O-hexuronide (66, **Table 1**) in the positive mode.


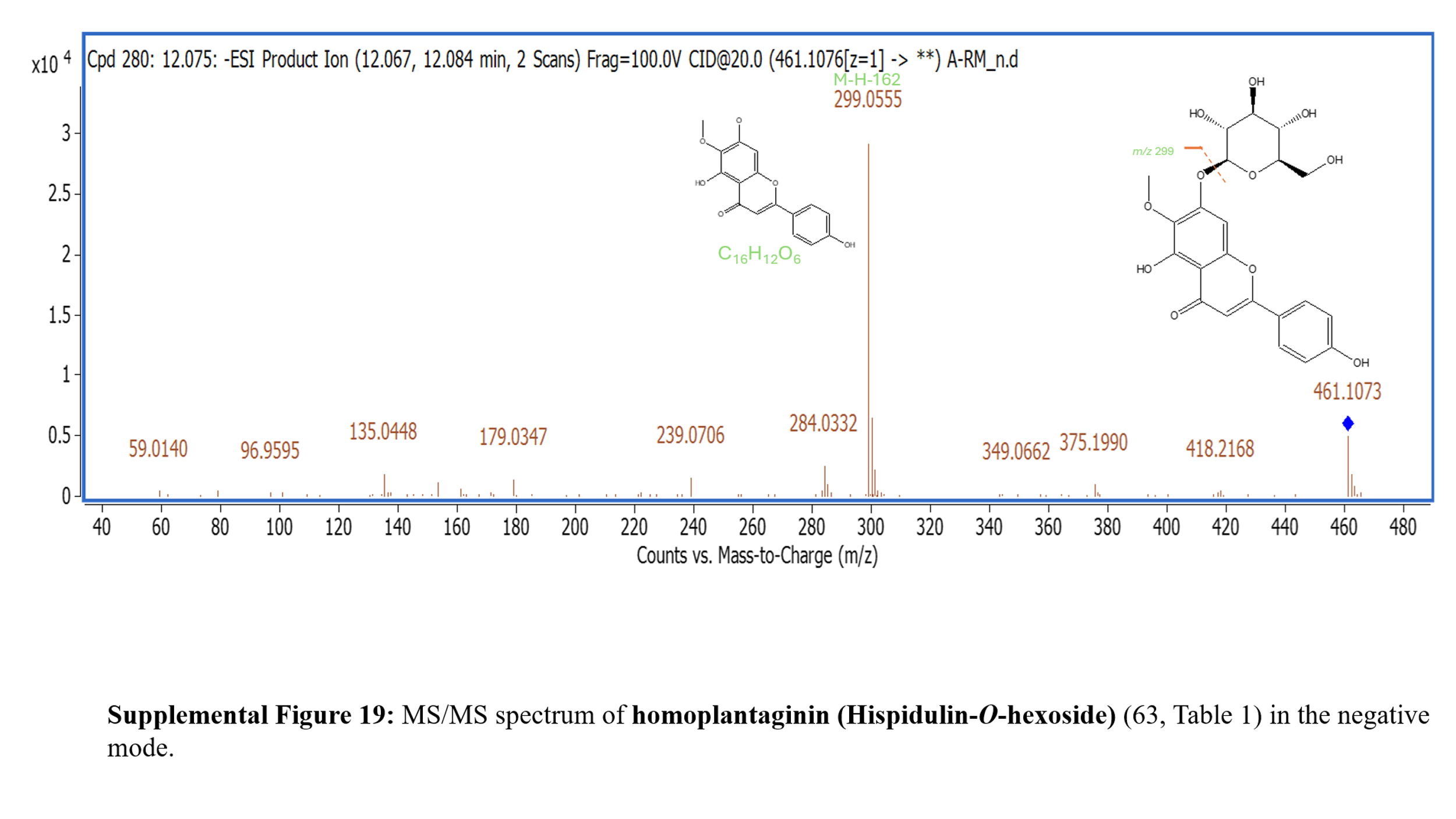


**Figure 18S:** MS/MS spectrum of homoplantaginin (Hispidulin-O-hexoside) (75, **Table 1**) in the negative mode.


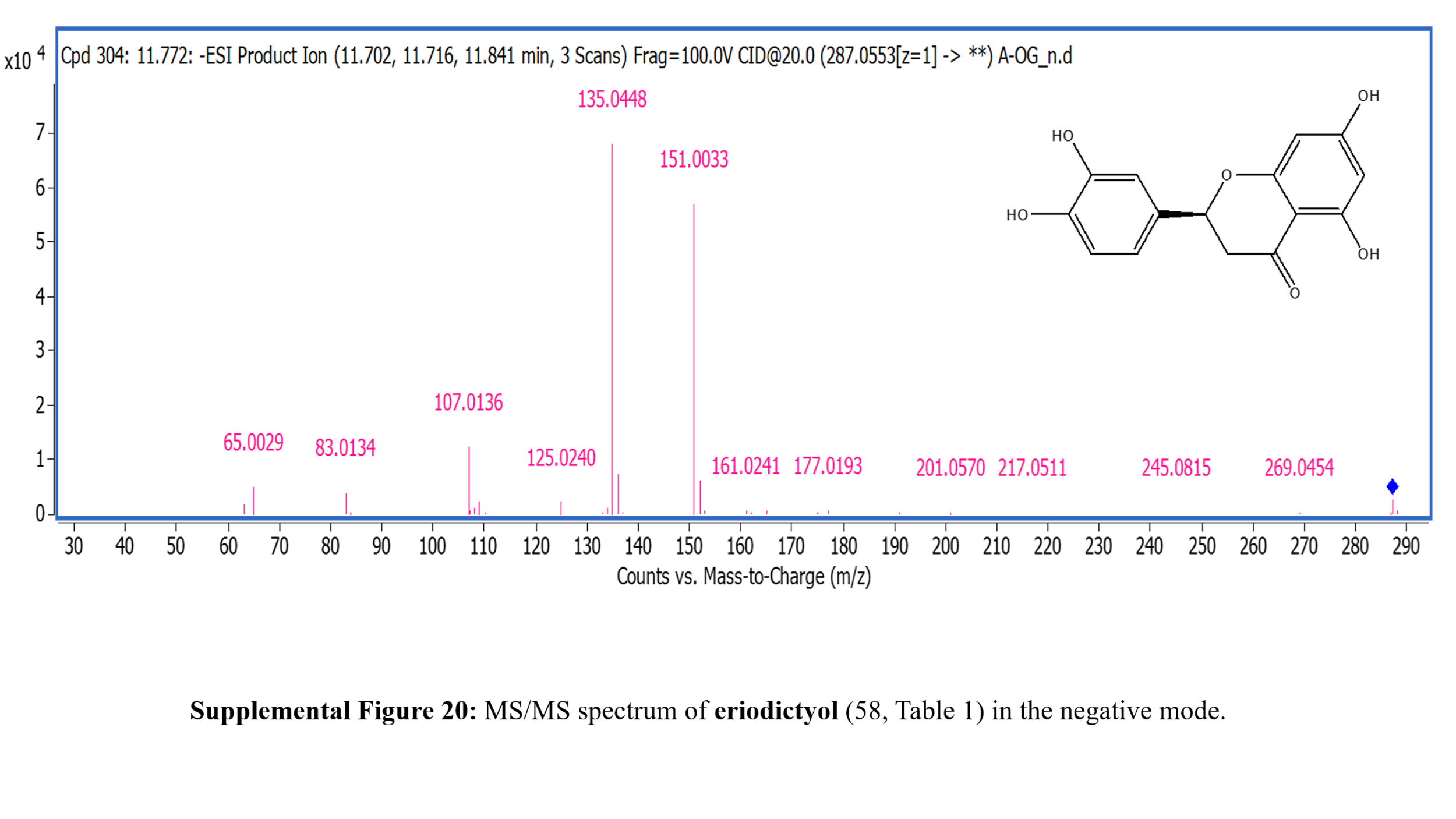


**Figure 19S:** MS/MS spectrum of eriodictyol (103, **Table** **1**) in the negative mode.


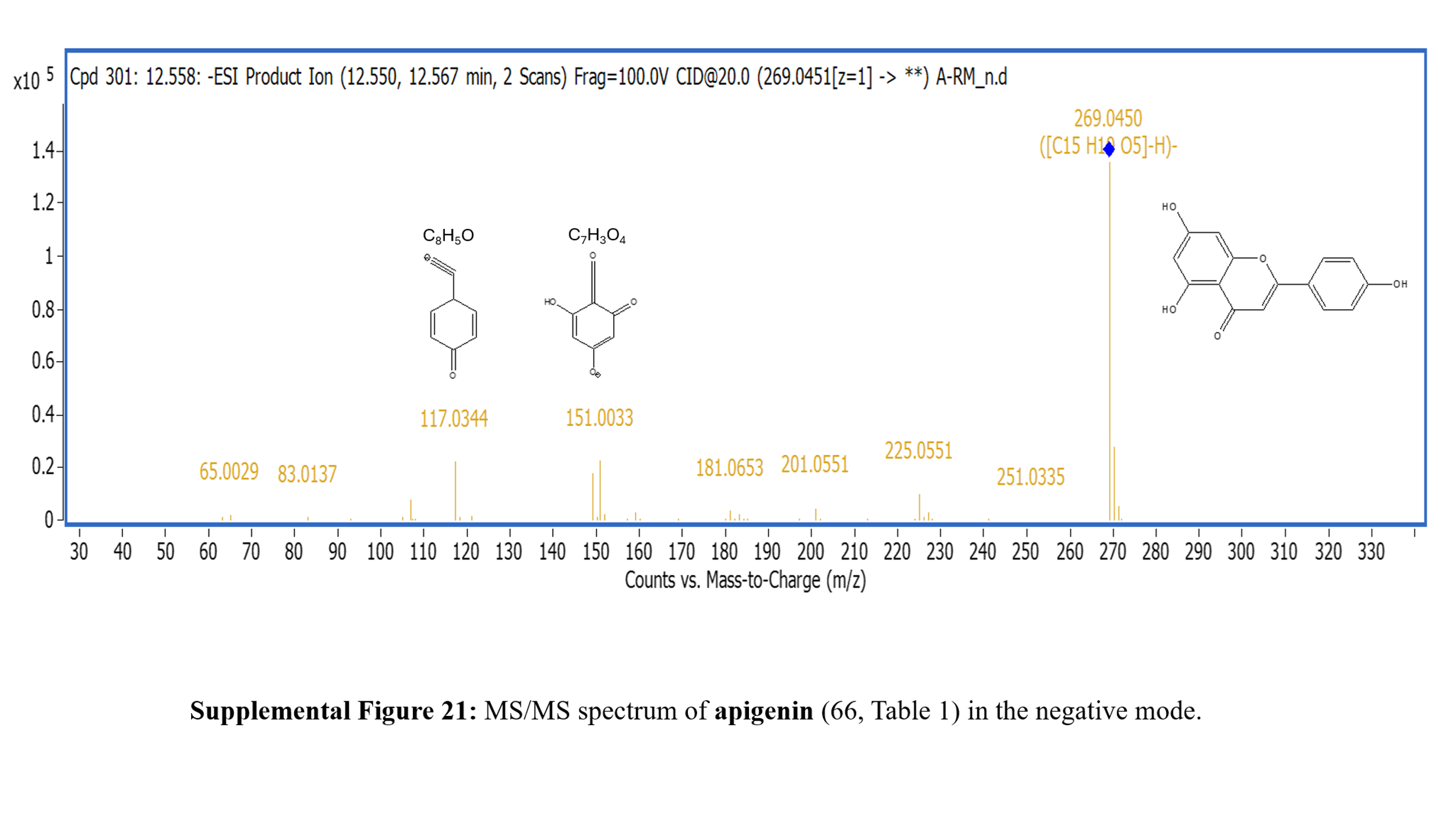


**Figure 20S:** MS/MS spectrum of apigenin (77, **Table 1**) in the negative mode.


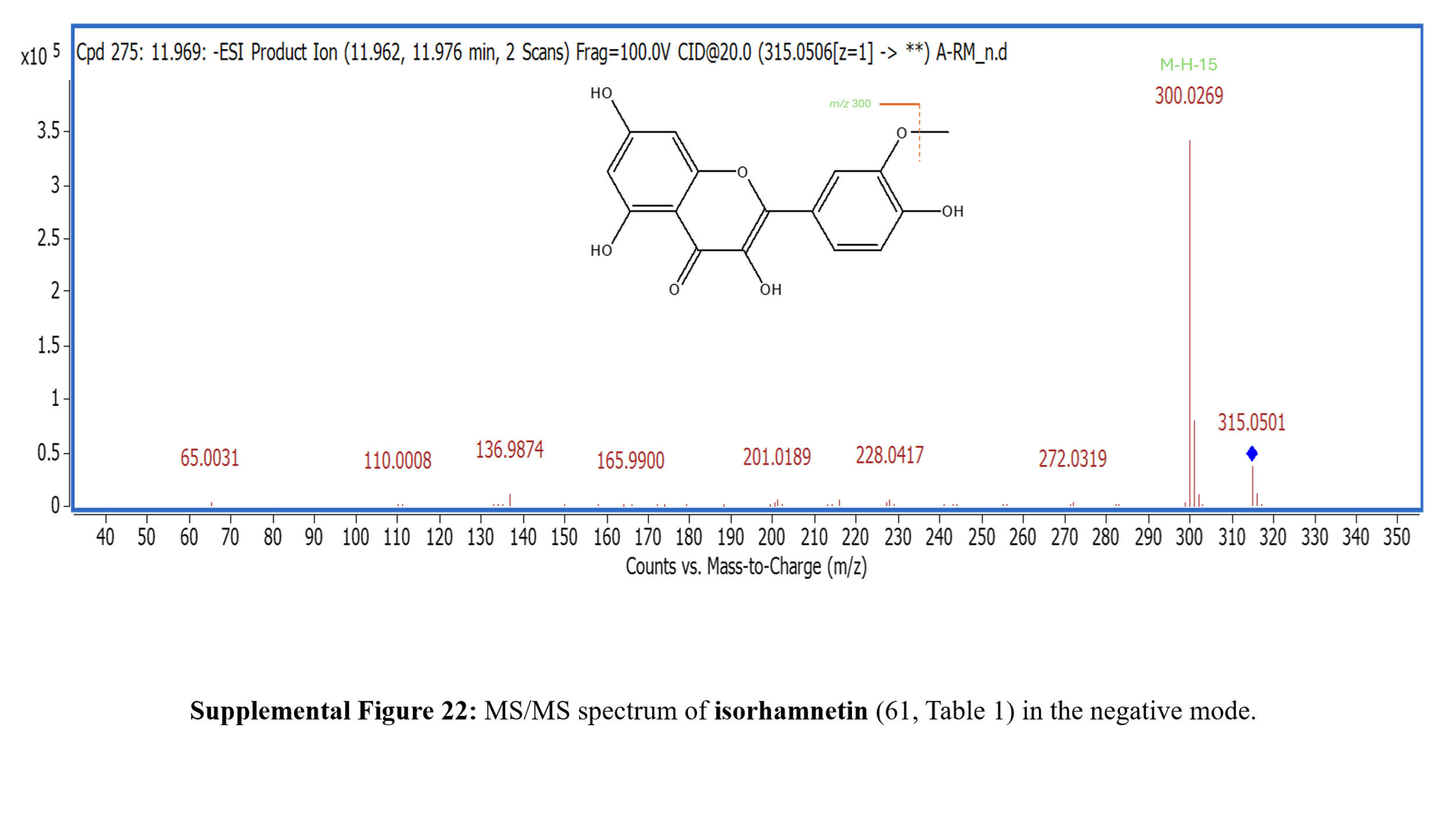


**Figure 21S:** MS/MS spectrum of isorhamnetin (97, **Table 1**) in the negative mode.


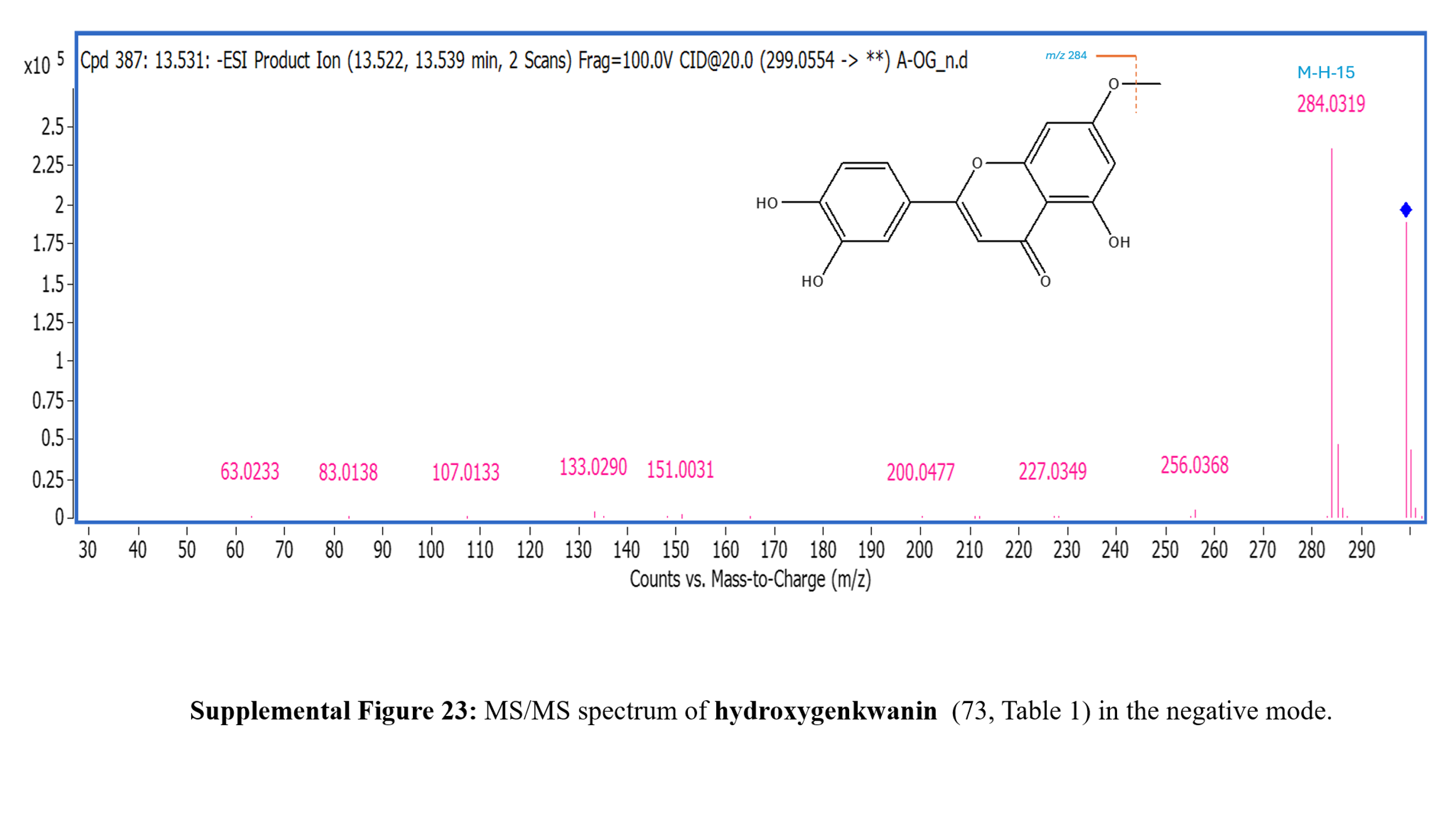


**Figure 22S:** MS/MS spectrum of hydroxygenkwanin (82, **Table 1**) in the negative mode.


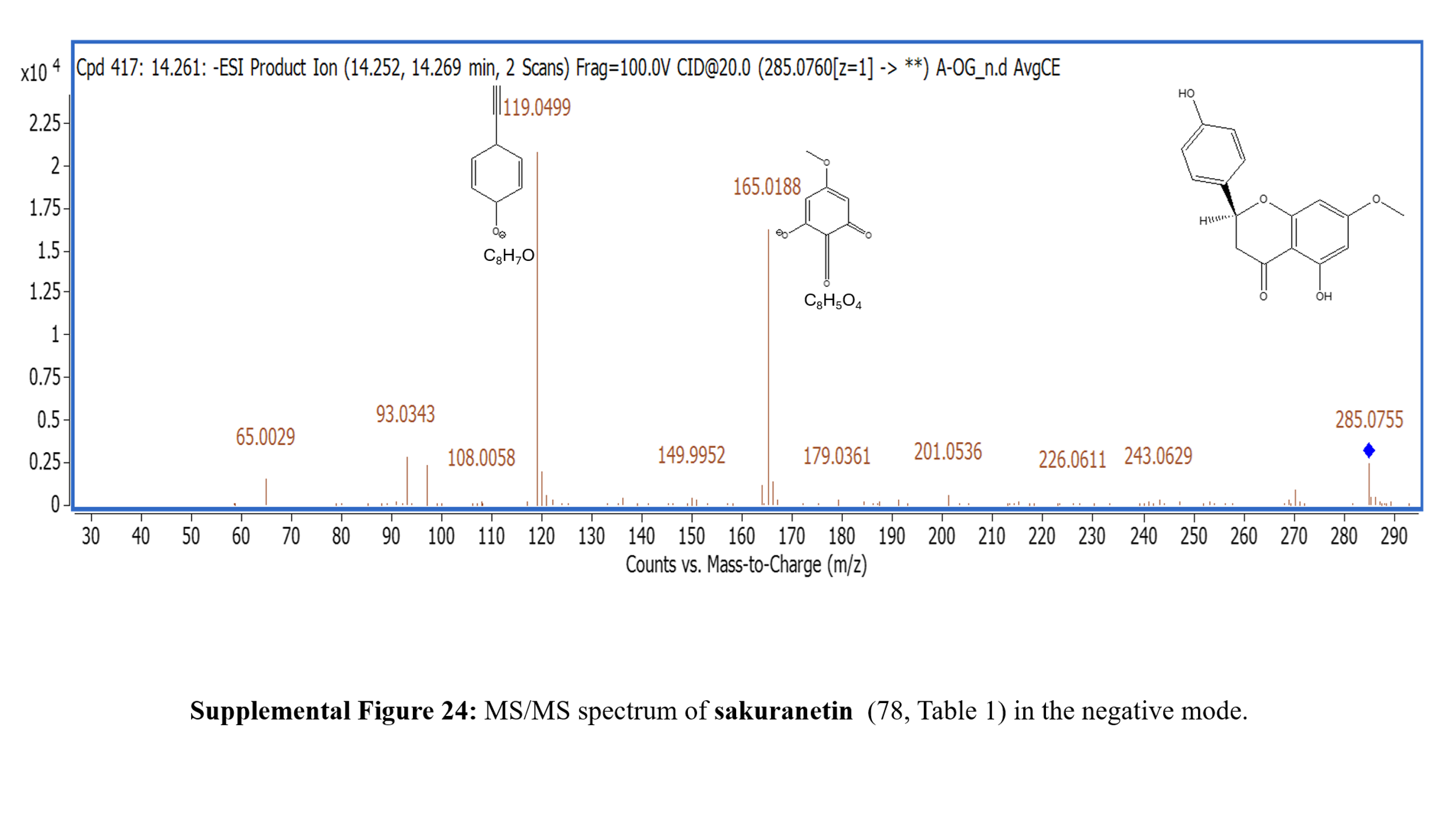


**Figure 23S:** MS/MS spectrum of sakuranetin (107, **Table 1**) in the negative mode.


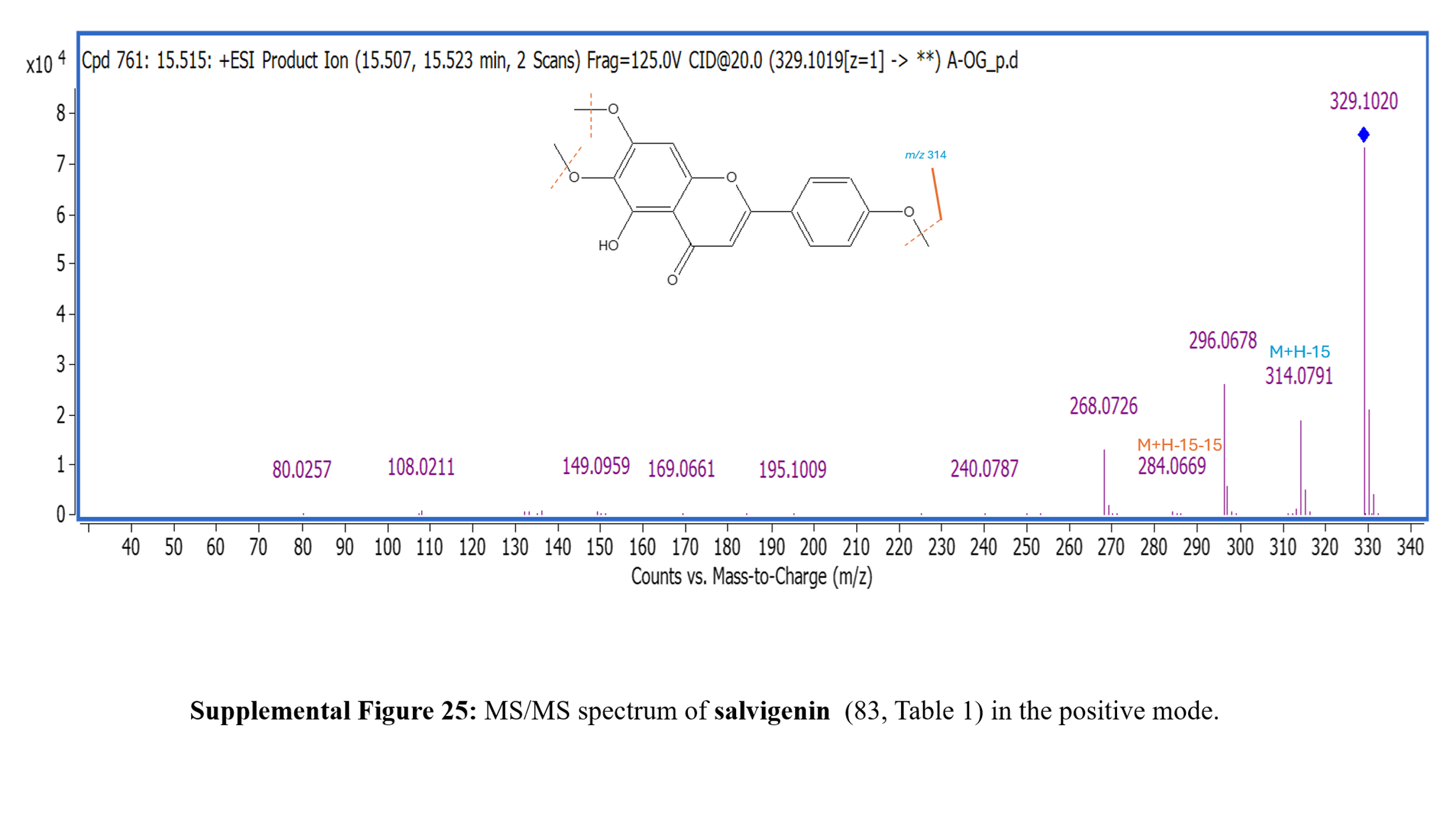


**Figure 24S:** MS/MS spectrum of salvigenin (90, **Table** **1**) in the positive mode.


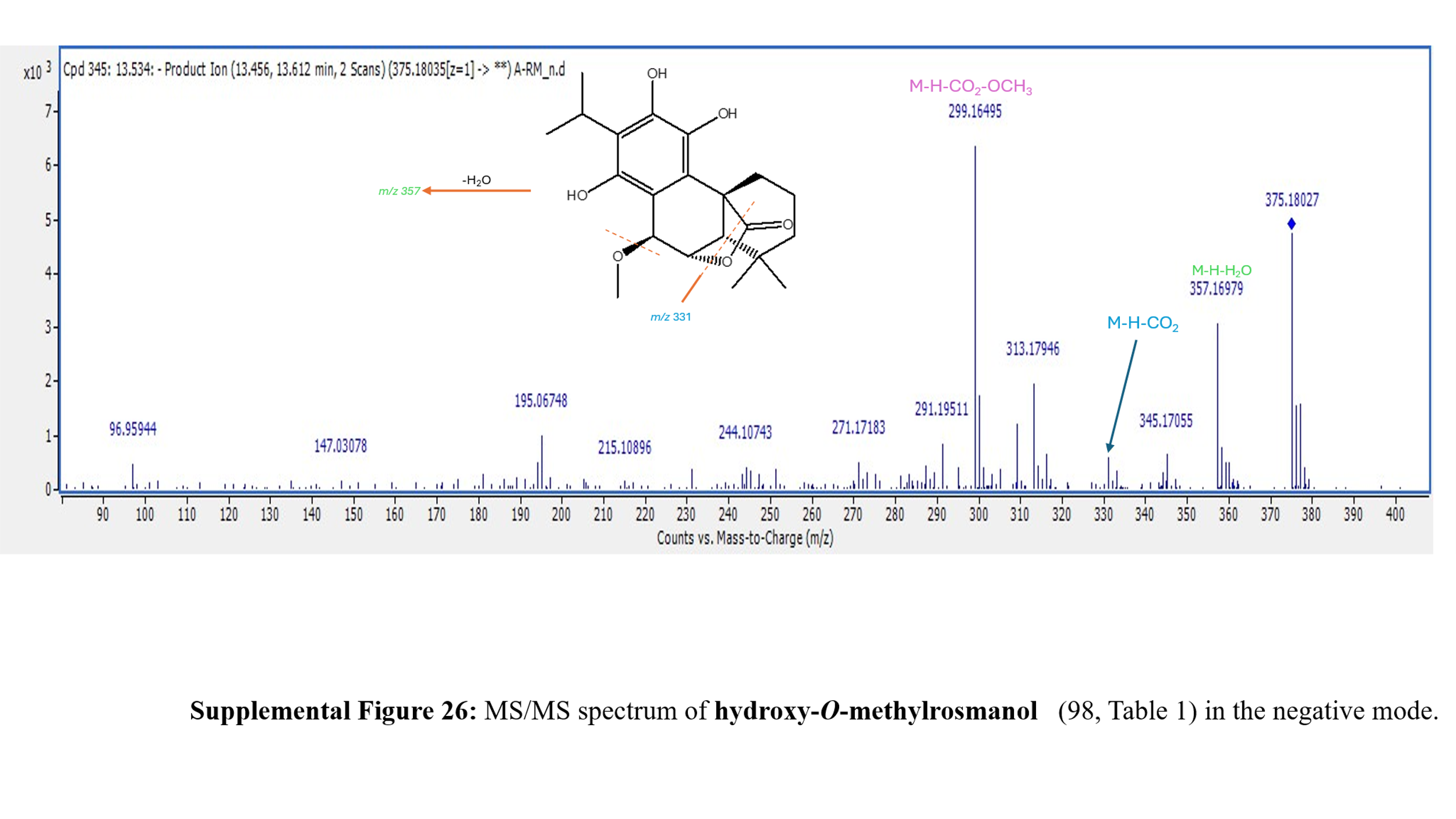


**Figure 25S:** MS/MS spectrum of hydroxy-O-methylrosmanol (116, **Table 1**) in the negative mode.


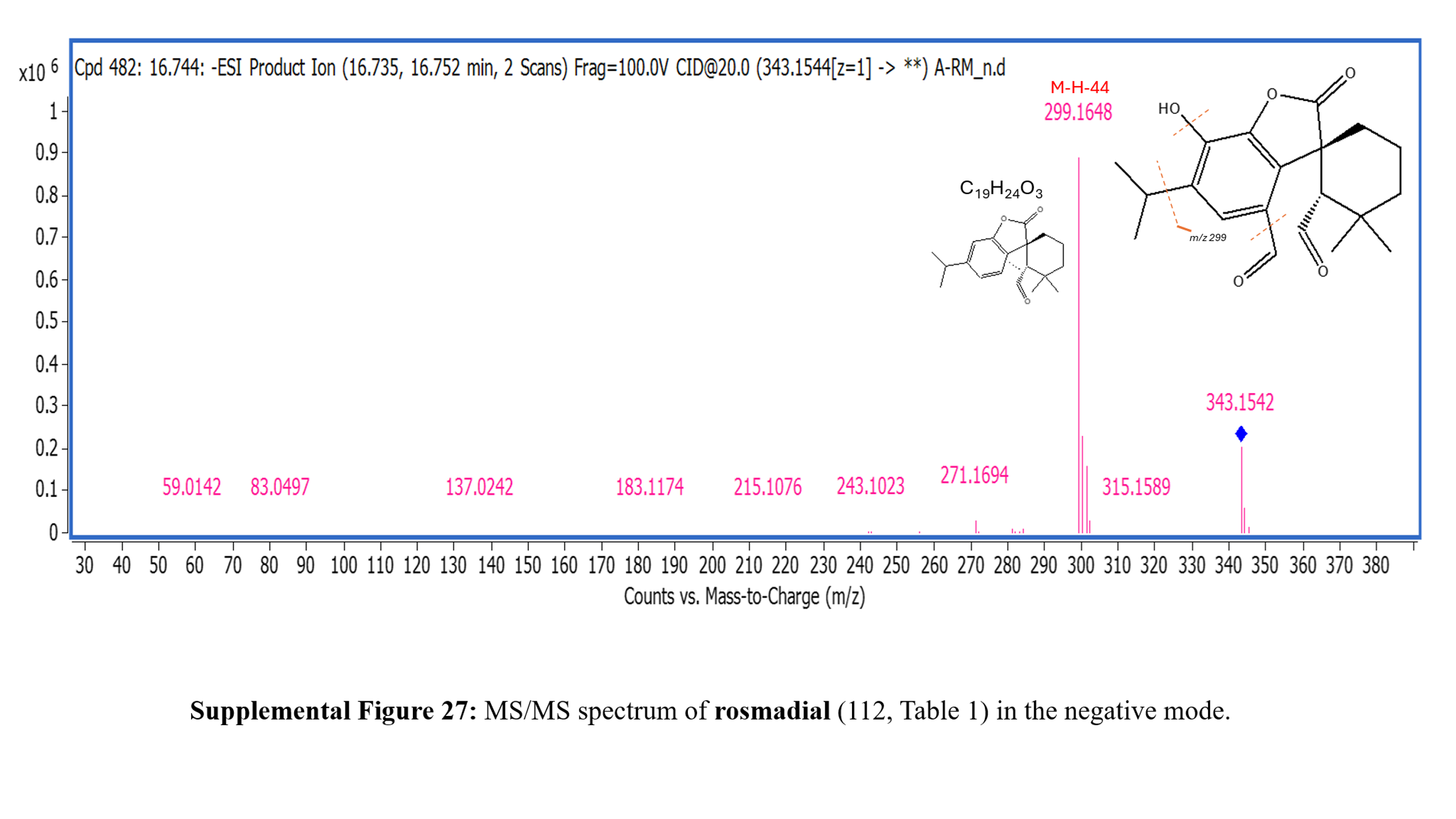


**Figure 26S:** MS/MS spectrum of rosmadial (130, **Table 1**) in the negative mode.


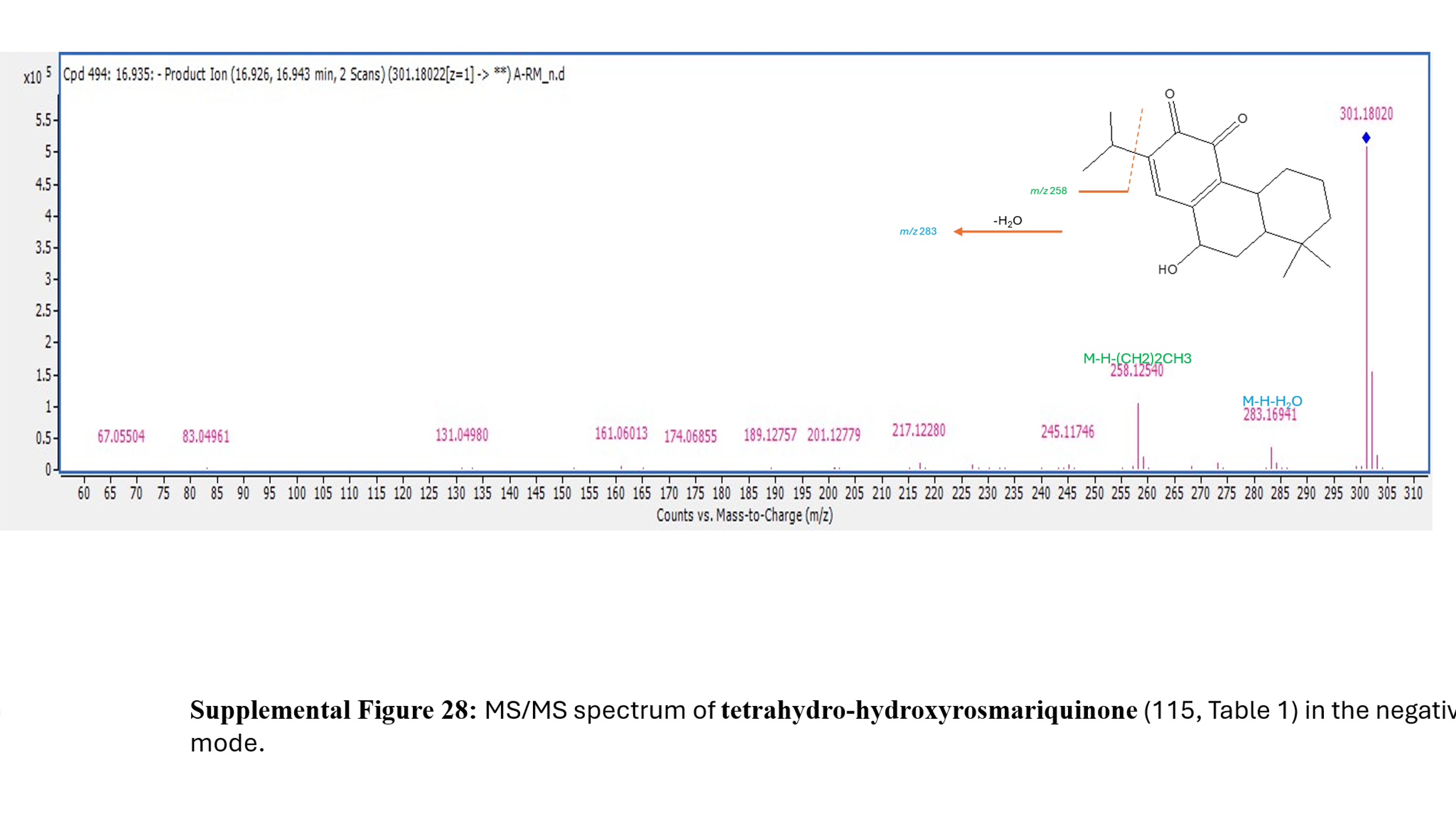


**Figure 27S:** MS/MS spectrum of tetrahydro-hydroxyrosmariquinone (133, **Table 1**) in the negative mode.


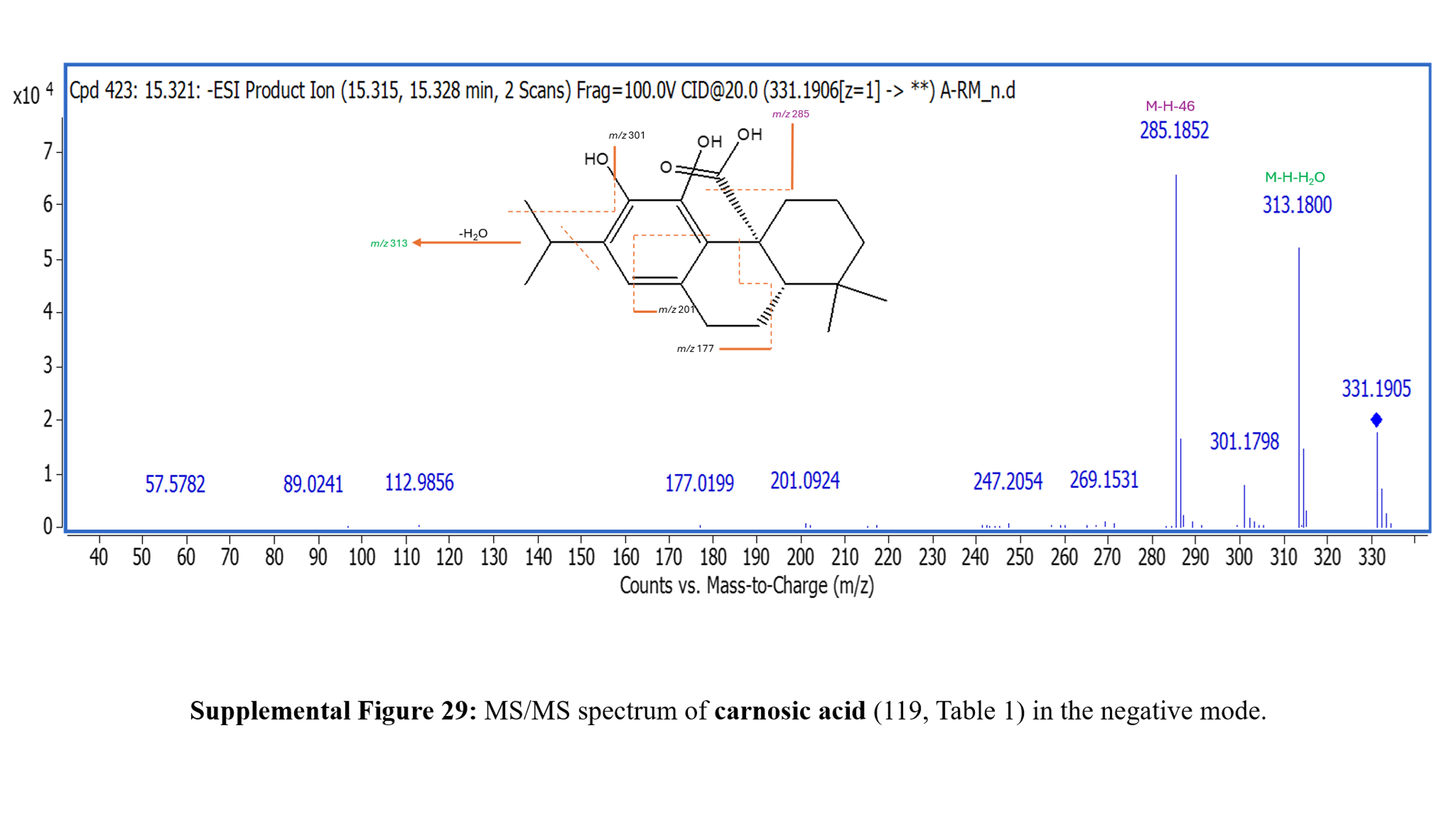


**Figure 28S:** MS/MS spectrum of carnosic acid (137, **Table 1**) in the negative mode.


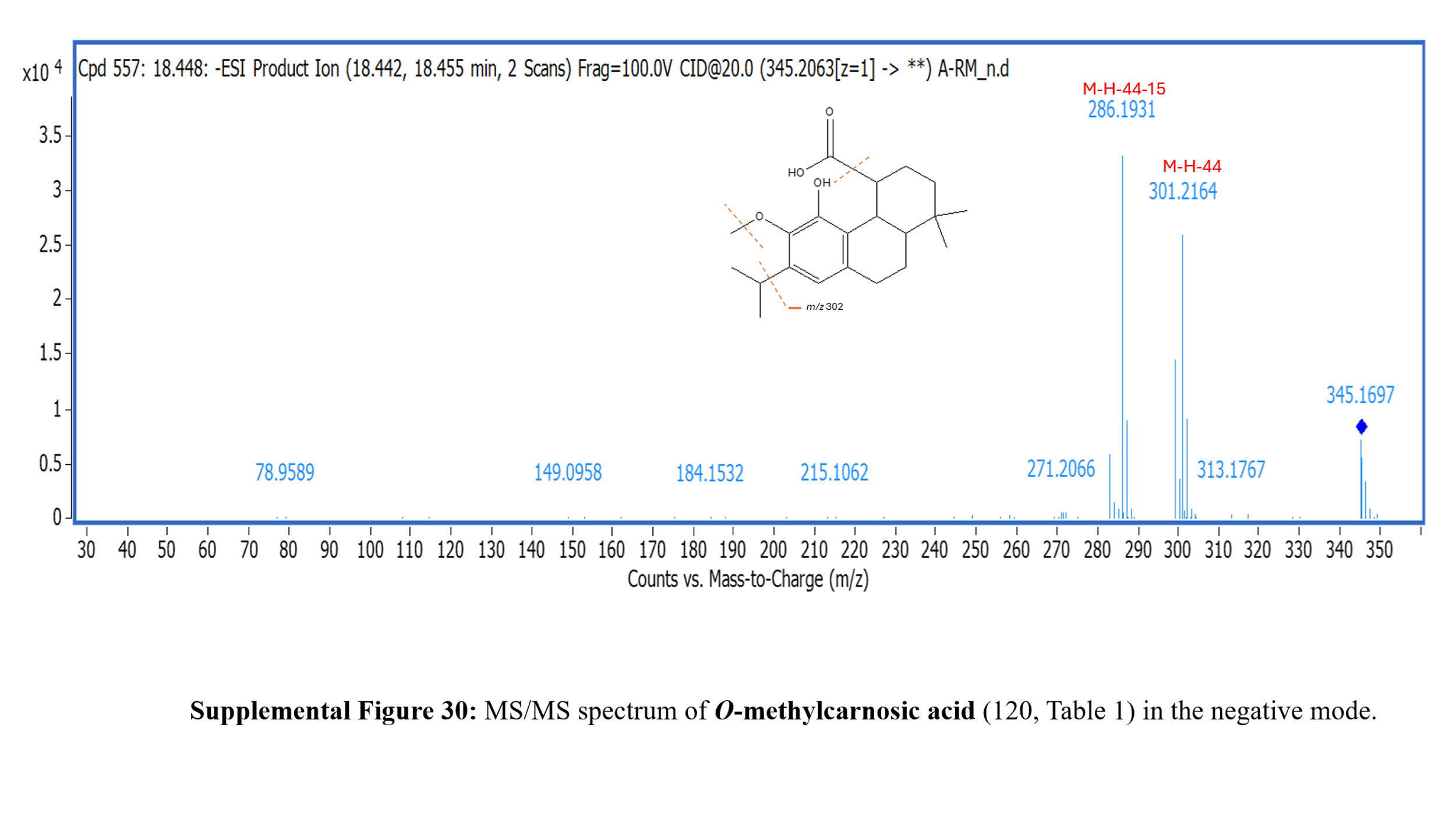


**Figure 29S:** MS/MS spectrum of O-methylcarnosic acid (138, **Table 1**) in the negative mode.


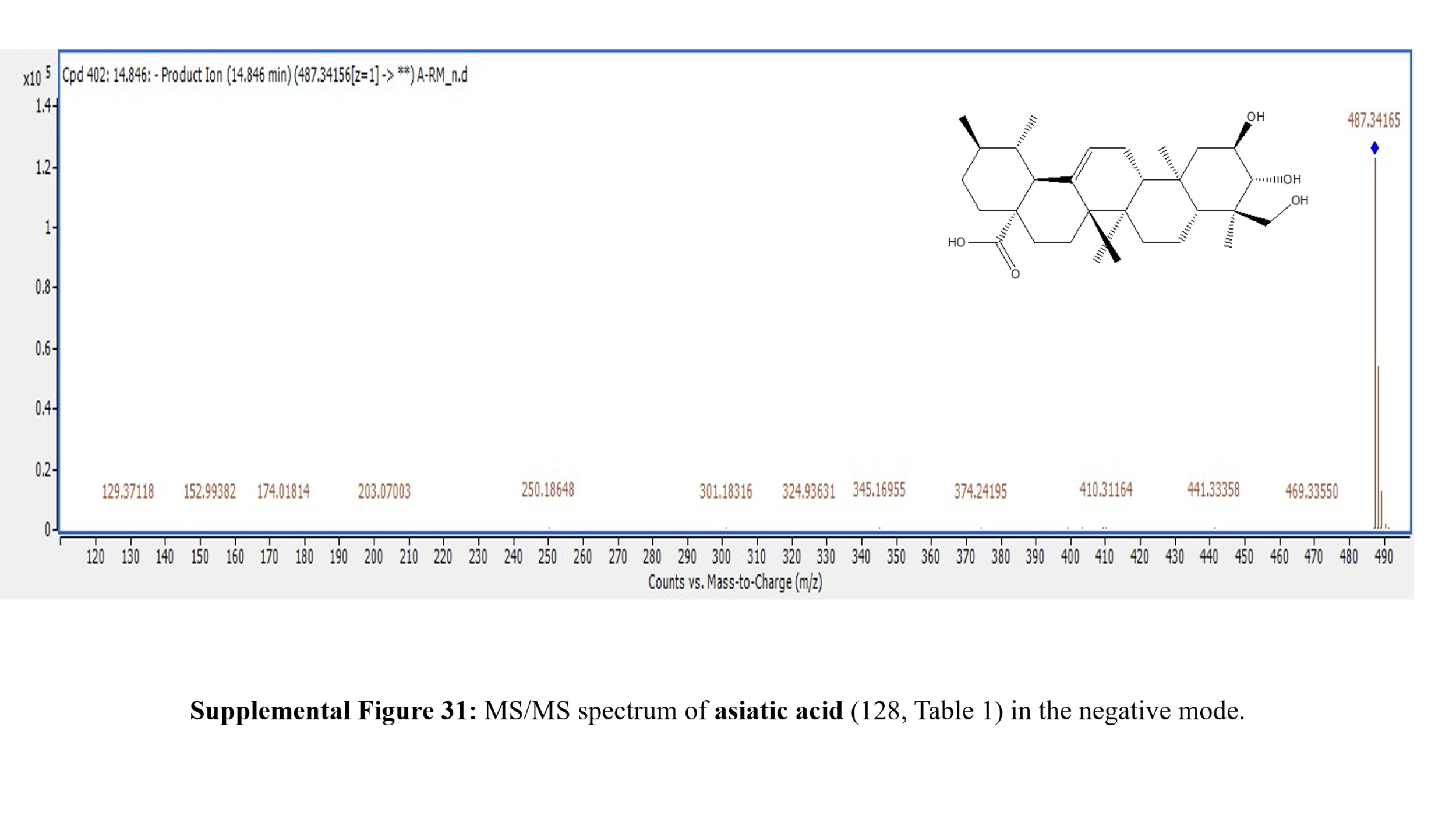


**Figure 30S:** MS/MS spectrum of asiatic acid (143, **Table 1**) in the negative mode.
